# HMGB1 facilitates repair of mitochondrial DNA damage and extends the lifespan of mutant ataxin-1 knock-in mice

**DOI:** 10.15252/emmm.201404392

**Published:** 2014-12-15

**Authors:** Hikaru Ito, Kyota Fujita, Kazuhiko Tagawa, Xigui Chen, Hidenori Homma, Toshikazu Sasabe, Jun Shimizu, Shigeomi Shimizu, Takuya Tamura, Shin-ichi Muramatsu, Hitoshi Okazawa

**Affiliations:** 1 Department of Neuropathology, Medical Research Institute, Tokyo Medical and Dental UniversityBunkyo-ku, Tokyo, Japan; 2 Department of Neurology, The University of TokyoBunkyo-ku, Tokyo, Japan; 3 Department of Pathological Cell Biology, Medical Research Institute, Tokyo Medical and Dental UniversityBunkyo-ku, Tokyo, Japan; 4 Department of Neurology, Jichi Medical UniversityShimotsuke, Tochigi, Japan; 5 Center for Brain Integration Research, Tokyo Medical and Dental UniversityBunkyo-ku, Tokyo, Japan

**Keywords:** AAV, DNA damage repair, HMGB1, mitochondria, SCA1

## Abstract

Mutant ataxin-1 (Atxn1), which causes spinocerebellar ataxia type 1 (SCA1), binds to and impairs the function of high-mobility group box 1 (HMGB1), a crucial nuclear protein that regulates DNA architectural changes essential for DNA damage repair and transcription. In this study, we established that transgenic or virus vector-mediated complementation with HMGB1 ameliorates motor dysfunction and prolongs lifespan in mutant Atxn1 knock-in (Atxn1-KI) mice. We identified mitochondrial DNA damage repair by HMGB1 as a novel molecular basis for this effect, in addition to the mechanisms already associated with HMGB1 function, such as nuclear DNA damage repair and nuclear transcription. The dysfunction and the improvement of mitochondrial DNA damage repair functions are tightly associated with the exacerbation and rescue, respectively, of symptoms, supporting the involvement of mitochondrial DNA quality control by HMGB1 in SCA1 pathology. Moreover, we show that the rescue of Purkinje cell dendrites and dendritic spines by HMGB1 could be downstream effects. Although extracellular HMGB1 triggers inflammation mediated by Toll-like receptor and receptor for advanced glycation end products, upregulation of intracellular HMGB1 does not induce such side effects. Thus, viral delivery of HMGB1 is a candidate approach by which to modify the disease progression of SCA1 even after the onset.

## Introduction

Spinocerebellar ataxia type 1 (SCA1) is one of the major groups of autosomal dominant hereditary cerebellar ataxia. The pathology shows dysfunctions and cell death of Purkinje cells in the cerebellum and motor neurons in the spinal cord. Correspondingly, patients show slowly progressive cerebellar ataxia, sometimes accompanied by neurogenic muscular atrophy mimicking motor neuron disease, and slow eye movement. The molecular mechanisms underlying the pathology have been gradually unravelled by extensive analyses by many groups. Importantly, overexpression of the normal form of the disease protein, ataxin-1 (Atxn1), has similar toxic effects on cells (Skinner *et al*, 1997), suggesting that an activity or interaction of mutant Atxn1, which normal Atxn1 also possesses, in excess causes neuronal dysfunction.

In this regard, the reciprocal interactions of Atxn1 with Capicua (CIC) and RNA binding motif protein 17 (RBM17) are of importance. Zoghbi's group and Orr's group found that mutant Atxn1 must be in its large native complexes to cause neurodegeneration and that its interactions with RBM17 are enhanced at the expense of interactions with CIC (Lam *et al*, 2006; Lim *et al*, 2008). Given that RBM17, also called splicing factor 45 (SPF45), is an RNA binding protein involved in splicing and that CIC/Capicua is a Sox2-like high-mobility group (HMG) protein involved in transcriptional repression, the shifted balance between splicing and transcription could broadly affect gene and protein expression profiles. We also found that polyglutamine binding protein 1 (PQBP1), a splicing factor involved in the U5 complex (Waragai *et al*, 2000; Zhang *et al*, 2000) at the step of exon–intron junction recognition by the spliceosome (Makarov *et al*, 2002; Makarova *et al*, 2004). It also binds to C-terminus of RNA polymerase II and similarly forms larger nuclear bodies through binding with Atxn1 (Okazawa *et al*, 2002), supporting the hypothesis that mutant Atxn1 shifted the balance of transcription and splicing to an abnormal state.

A current goal, therefore, is to find efficient methods to rescue the imbalance of gene expression and to determine the molecular mechanism that underlies the rescue. We previously searched for nuclear proteins quantitatively affected by mutant Atxn1 in neurons and found a significant decrease in high-mobility group box (HMGB) 1/2 proteins in the soluble nuclear fraction. The decrease was mainly attributed to proteasomal degradation and sequestration to inclusion bodies of the mutant Atxn1–HMGB1 complex (Qi *et al*, 2007). HMGB supplementation actually ameliorates eye degeneration in an SCA1 fly model and restores impaired DNA damage repair (Qi *et al*, 2007).

HMGB1/2 are architectural proteins that regulate the higher structure of genomic DNA. Consequently, they influence a broad range of nuclear functions, including transcription and DNA damage repair (Muller *et al*, 2001; Travers, 2003). HMGB1/2 possess HMG boxes like another Atxn1 binding partner, HMG-box transcription factor 1 (HBP1), which includes one Atxn1-HBP1 shared (AXH) domain (de Chiara *et al*, 2003) and one HMG box. The AXH domain may contribute to the self-association (Burright *et al*, 1997) or RNA binding (Yue *et al*, 2001) of Atxn1. These possibilities suggest that, in addition to architectural change of DNA, HMGB1 might influence the functional link between the HMG-box proteins (HBP1, Capicua, HMGB1/2) related to transcription and the AXH-domain proteins (Atxn1, HBP1) related to RNA metabolism.

In this study, we extend our previous findings in primary neurons and *Drosophila* and show that HMGB1 has a strong therapeutic effect on multiple phenotypes of mutant Atxn1-KI mice. In addition to the therapeutic effect of transgenic expression of HMGB1, we show that a single injection of an adeno-associated virus type 1 (AAV1) vector carrying HMGB1 is effective *in vivo* even after the onset of symptoms in mutant Atxn1-KI mice and that the therapeutic effect persists for more than 8 weeks. Moreover, we reveal mitochondrial DNA damage repair by HMGB1 as a new mechanism to rescue the pathology of SCA1.

## Results

### HMGB1 restores SCA1-KI mouse symptoms without affecting aggregation

We generated an HMGB1 transgenic mouse model on a C57BL/6 background expressing HMGB1 under the control of a 1.9-kb rat neuron-specific enhancer/promoter (Supplementary Fig S1A). Genome integration of the full-length construct was confirmed by PCR with genomic DNA from HMGB1-Tg mice (Supplementary Fig S1B). HMGB1 protein tagged with a 3× FLAG sequence was detected in total brain tissue by Western blot analysis (Supplementary Fig S1C) and in neurons of various brain regions including cerebral cortex, brain stem, hypothalamus and cerebellum by immunohistochemistry (Supplementary Fig S1D). In the cerebellum, expression of HMGB1-FLAG was prominent in Purkinje cells (Supplementary Fig S1E), which was confirmed by immunohistochemistry with anti-calbindin and anti-NeuN antibodies (Supplementary Fig S1F). HMGB1-Tg mice did not show pathological changes, such as decreased Purkinje cell number or decreased thickness of the molecular layer (Supplementary Fig S1G).

The major concern in the overexpression is that HMGB1 may trigger inflammatory responses via Toll-like receptor 2/4 (TLR2/4) or receptor for advanced glycation end products (RAGE) when released into the extracellular space (Park *et al*, 2006; Yu *et al*, 2006). Therefore, we tested inflammatory responses, including lymphocyte infiltration and microglia/macrophage activation, in the brains of HMGB1-Tg and non-Tg littermate mice (Supplementary Fig S2A). Peritoneal injection of lipopolysaccharide (LPS) into background mice (C57BL/6) was performed to make a positive control for brain inflammation. Immunohistochemistry of the cerebellum with antibodies against multiple inflammation marker proteins, such as CD4 (a helper T-cell marker), CD8 (a cytotoxic T-cell marker), CD11c (a dendritic cell marker) and microglial response factor-1 (a microglial marker), did not reveal any inflammation response in HMGB1-Tg mice (Supplementary Fig S2A). The cerebral cortex showed similar findings (data not shown). These results suggest that HMGB1 does not trigger brain inflammation in our mouse model (Supplementary Fig S1).

**Figure 1:**
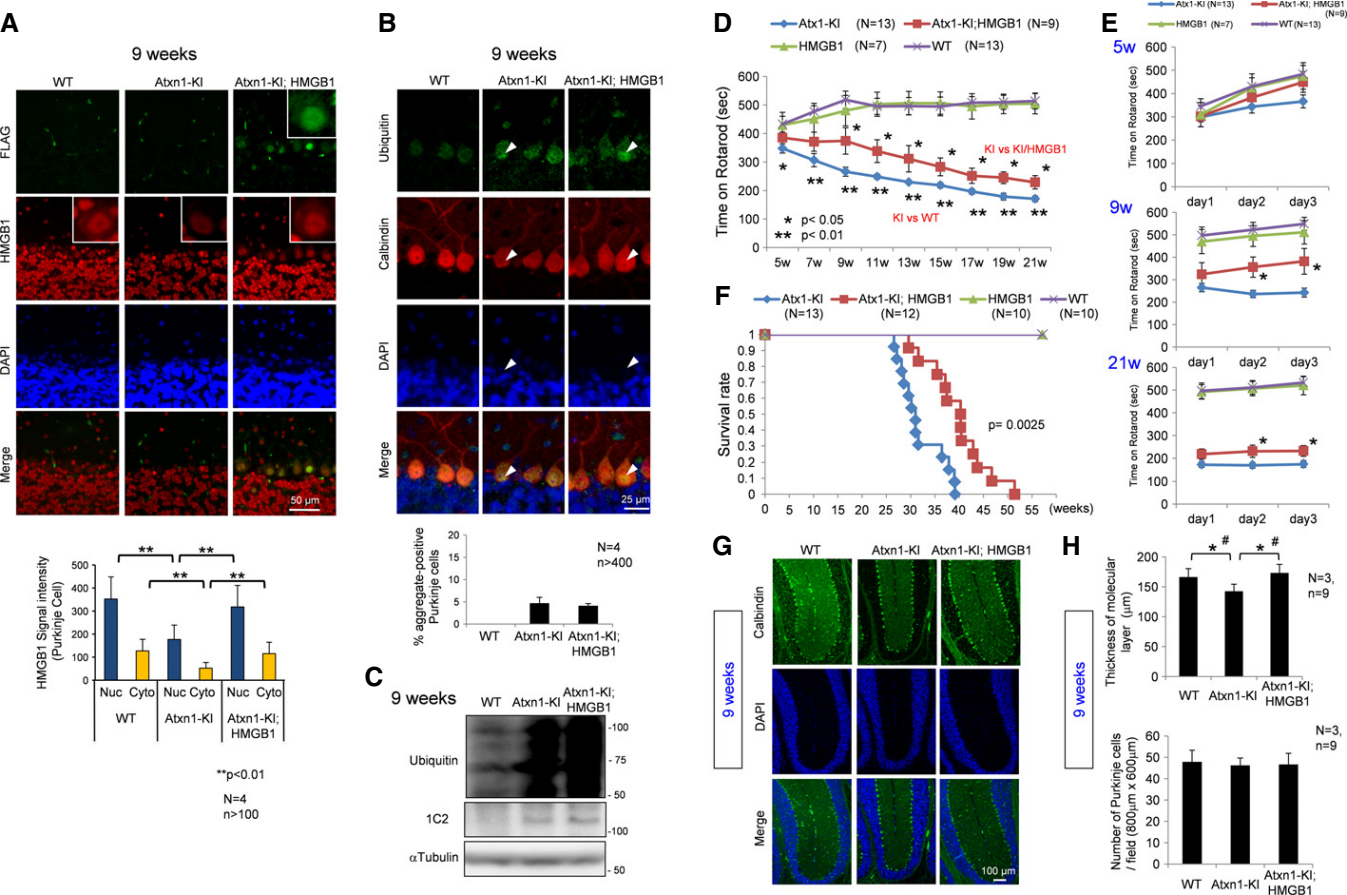
Symptomatic rescue of double-transgenic mice without decreased protein aggregation The expression of exogenous HMGB1-FLAG (detected with an anti-FLAG antibody) and endogenous+exogenous HMGB1 (detected with an anti-HMGB1 antibody) was tested by immunohistochemical analysis. Nuclear and cytoplasmic signals were reduced in Atxn1-KI mice but restored in double-transgenic mice. The mean ± SD are shown in the lower graph.Aggregate formation was tested by immunohistochemical analysis of cerebellar tissues of background C57BL/6 mice, mutant Atxn1-KI mice and double-transgenic mice. Ubiquitin-positive aggregates were observed in mutant Atxn1-KI mice and double-transgenic mice (upper panels); the ratio of aggregate-positive Purkinje cells did not differ between the four mutant Atxn1-KI mice and the four double-transgenic mice (lower graph). The data are presented as mean ± SD.Western blot analysis with anti-ubiquitin antibody confirmed that aggregate formation was similar in mutant Atxn1-KI mice and double-transgenic mice.The rotarod performance test revealed improvement in the motor activity of double-transgenic mice over that of mutant Atxn1-KI mice. The time on rotarod was the mean value of the times from day 1 to day 3. The symptomatic onset in mutant Atxn1-KI mice was 7 weeks; improvement in double-transgenic mice lasted from 9 to 21 weeks. The data are presented as mean ± SD. Statistical analysis was performed with the Bonferroni–Dunn test and Student's *t*-test.Details of the rotarod performance of the three genotypes of mice (WT, Atxn1-KI and double-transgenic) from day 1 to day 3 at 5, 9 and 21 weeks. The data are presented as mean ± SD.The survival rates of the four genotypes of mice. The mean and maximum lifespans of double-transgenic mice were nearly 30% longer than those of mutant Atxn1-KI mice. The 50% survival duration was extended from 217 days (Atxn1-KI) to 282 days (Atxn1-KI;HMGB1), and the maximum survival duration was extended from 274 days (Atxn1-KI) to 360 days (Atxn1-KI;HMGB1). The effect of HMGB1 on the lifespan was assessed by Kaplan–Meier analysis, and the end-point postponement was significant in the log-rank test (*P* = 0.0025).Histological evaluation of the double-transgenic mice.Quantitative analysis of histological parameters in the three groups of mice showed that reduction in the molecular layer thickness was reversed in double-transgenic mice (upper graph; Student's *t*-test, *P* < 0.05). The mean thickness was quantified in more than 10 visual fields per mouse, and the mean ± SD was calculated for nine mice in each group. The number of Purkinje cells, similarly calculated, was not changed in mutant Atxn1-KI mice or in double-transgenic mice (lower graph). The data are presented as mean ± SD. *P < 0.05 in Student's *t*-test, ^#^*P* < 0.05 in one-way ANOVA followed by *post hoc* Tukey's HSD (honestly significant difference) test. Source data are available online for this figure.

We next mated HMGB1-Tg mice with Atxn1-154Q knock-in mice (Atxn1-KI mice) of the same background C57BL/6 and generated double-transgenic mice (Atxn1-KI;HMGB1 mice). As reported previously (Qi *et al*, 2007), HMGB1 was decreased in the nucleus of Purkinje cells of Atxn1-KI mice (Fig1A). In addition, HMGB1 was decreased in the cytoplasm of Purkinje cells (Fig1A). In Atxn1-KI;HMGB1 mice, the expression of HMGB1 was restored to the level of background C57BL/6 mice in the nucleus and cytoplasm of Purkinje cells at 9 weeks (Fig1A). The ubiquitin-positive aggregation of mutant Atxn1 was not largely affected by expression of exogenous HMGB1 when assessed by immunohistochemistry and Western blot analysis (Fig1B and C), even though HMGB1 is a binding partner of mutant Atxn1 (Qi *et al*, 2007), probably because exogenous expression of HMGB1 was far smaller than endogenous expression (Supplementary Fig S1C). Meanwhile, immunostaining showed recovery of HMGB1 in the Purkinje cells of Atxn1-KI;HMGB1 mice (Fig1A and B), reflecting an increase in Atxn1-unbound HMGB1 reactive to antibody.

Despite continued aggregation of mutant Atxn1, the Atxn1-KI;HMGB1 mice showed a remarkable improvement of motor activity in the rotarod test (Fig1D and E). The improvement was obvious in comparison of multiple genotypes including Ku70-Tg mice and Atxn1-KI;Ku70 double-transgenic mice (Supplementary Fig S1H). Transgenic co-expression of Ku70 was effective for a Huntington's disease (HD) mouse model (R6/2 mice) (Enokido *et al*, 2010) but not effective for Atxn1-KI (Supplementary Fig S1H).

Moreover, the lifespan was extended dramatically (Fig1F). The 50% and maximum survival durations were elongated from 217 days (Atxn1-KI) to 282 days (Atxn1-KI;HMGB1) and from 274 days (Atxn1-KI) to 360 days (Atxn1-KI;HMGB1), respectively. This elongation rate is one of the best results reported with Atxn1-KI mice (Watase *et al*, 2007). In our analysis, the thickness of the molecular layer was decreased at the onset of symptoms (9 weeks). Consistent with the improved motor activity, pathological examination revealed recovery of the molecular layer thickness in Atxn1-KI;HMGB1 mice at 9 weeks (Fig1G and H).

### HMGB1 expression restores nuclear DNA damage *in vivo*

Given that HMGB1 promotes DNA damage repair via architectural changes in genomic DNA (Bianchi & Beltrame, 1998; Agresti & Bianchi, 2003; Travers, 2003), we evaluated double-strand breaks (DSB) in genomic DNA by immunostaining and Western blot of phosphorylated histone H2AX (γH2AX) or p53 binding protein 1 (53BP1) (Supplementary Fig S3). γH2AX signals were increased in Purkinje cells of mutant Atxn1-KI mice and recovered in those of Atxn1-KI;HMGB1 mice in immunohistochemistry (Supplementary Fig S3A), which was confirmed quantitatively (Supplementary Fig S3B). The results were reproduced by 53BP1 in both methods (Supplementary Fig S3A and C). Western blot analyses with γH2AX and 53BP1 antibodies also revealed the increase of DSB in Atxn1-KI mice and the recovery in Atxn1-KI;HMGB1 mice (Supplementary Fig S3D and E). Moreover, a negative relationship was observed between the γH2AX signal and HMGB1 signal in the Purkinje cells of Atxn1-KI mice (Supplementary Fig S3F and G). These results indicate that HMGB1 supplementation rescues DNA damage in Atxn1-KI mice, as expected from our previous results with primary neuronal cultures (Qi *et al*, 2007).

### Mutant Atxn1 reduces mitochondrial HMGB1

**Figure 2:**
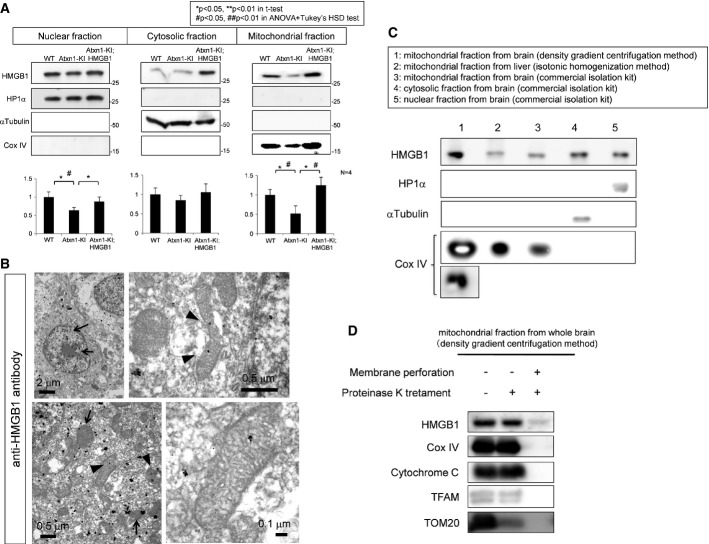
Mitochondrial HMGB1 is linked to mitochondrial functions Western blot with separated cellular components from the cerebellar tissues of the three genotypes of mice showed that HMGB1 was decreased not only in the nucleus but also in the cytosol and mitochondria of mutant Atxn1-KI mice. The downregulation in the three subcellular fractions was reversed in the double-transgenic mice. The band intensity was quantified and is shown in the lower graphs. The data are presented as mean ± SD. Statistical analysis involved Student's *t*-test and one-way ANOVA followed by *post hoc* Tukey's HSD (honestly significant difference) test.Immunoelectron microscopy of the cerebellar cortex of control mice, with staining using an anti-HMGB1 antibody. Nucleus-dominant distribution of the gold–silver complex particles was found (arrows in the upper left panel), although the particles were also found in the cytoplasm. In the cytoplasm (lower left panel), the mitochondrial membrane (arrow) and matrix (arrowhead) were stained. Higher-magnification images of the mitochondrial matrix staining are shown in the upper and lower right panels.Western blot detection of HMGB1 in mitochondria purified by multiple methods. Lane 1: the mitochondrial fraction purified from the brain by the Percoll density gradient centrifugation method (Sims & Anderson, 2008), Lane 2: the mitochondrial fraction purified from the mouse liver by the other centrifugation method (Shimizu *et al*, 1998), Lane 3: mitochondrial fraction purified from the mouse brain using a commercial isolation kit), Lane 4: the cytosol fraction from the mouse brain isolated using the Mitochondria Isolation Kit (Thermo Scientific, IL, USA) and Lane 5: a nuclear fraction from the mouse brain isolated using the same kit. All the methods revealed the presence of HMGB1 in mitochondria.Proteinase K was added to the mitochondrial fraction prepared by the Percoll density gradient centrifugation method, to exclude the possibility of contamination with the nuclear or cytosolic fraction. Addition of proteinase K before membrane perforation did not affect HMGB1 dramatically decreased HMGB1 after membrane perforation, indicating that HMGB1 is located in the mitochondria. Other mitochondrial proteins including Cox IV and cytochrome c at the inner mitochondrial membrane or TFAM that binds to mitochondrial DNA inside the inner mitochondrial membrane showed a similar pattern of changes, whereas Tom20 at the outer mitochondrial membrane was digested before membrane perforation. Source data are available online for this figure.

HMGB1 is reported to exist in the cytoplasm and to regulate autophagy (Tang *et al*, 2010, 2011). Cytoplasmic HMGB1 binds to phosphorylated Beclin1 and deprives it from Bcl2. The HMGB1–Beclin1 complex accelerates autophagy and contributes to mitochondrial quality control (Tang *et al*, 2011). However, it is not yet clear exactly where in the cytoplasm HMG1 is located. Unexpectedly, our Western blot analysis with fractionated cellular compartments from mouse cerebellar tissues revealed mitochondrial HMGB1 in addition to the nuclear and cytosolic HMGB1 (Fig2A). Moreover, HMGB1 in the mitochondria fraction was decreased in Atxn1-KI mice and recovered in Atxn1-KI;HMGB1 mice (Fig2A). Consistently, overlapped signals between HMGB1 and Cox IV were decreased in Atxn1-KI mice in comparison with the background mice and restored in Atxn1-KI;HMGB1 mice (Supplementary Fig S4A).

To examine mitochondrial localization of HMGB1, we performed immunoelectron microscopy of cerebellar tissues from wild-type mice with anti-HMGB1 antibody. Silver staining, which enlarges gold particles by the complex, was used to enhance reaction (Fig2B). As expected, we found gold particles in the matrix of mitochondria (Fig2B, arrowhead). Peri-mitochondrial deposition of gold particles (Fig2B, arrow) might be consistent with autophagy-related function of HMGB1. The three mouse genotypes were further examined by immunoelectron microscopy (Supplementary Fig S4B). Grain-positive mitochondria was decreased in Atxn1-KI mice but recovered in Atxn1-KI;HMGB1 mice (Supplementary Fig S4B). Immunostaining with normal IgG did not reveal grains in electron microscopy (Supplementary Fig S4C).

In order to confirm existence of HMGB1 in mitochondria, we purified mitochondrial fraction by Percoll density gradient centrifugation method (Sims & Anderson, 2008) and by isotonic homogenization method (Shimizu *et al*, 1998), in addition to mitochondrial preparation using commercial kit. In mitochondrial fractions prepared by both methods, we detected the band of HMGB1 by Western blot (Fig2C). When mitochondrial fraction by Percoll density gradient centrifugation method was treated with proteinase K before membrane perforation, HMGB1 was not digested just like Cox IV, cytochrome c and TFAM, a transcription factor for mitochondrial genome that are localized at or inside of inner mitochondrial membrane (Fig2D) (Parisi & Clayton, 1991). Tom20 at outer mitochondrial membrane was digested by proteinase K under the similar condition (Fig2D), supporting the digestion was sufficient for proteins anchored or attached to the mitochondrial surface. After membrane perforation, all these mitochondrial proteins were completely degraded by proteinase K (Fig2D). These results supported that HMGB1 exists inside of mitochondrial membrane.

### HMGB1 may restore mitochondrial function through non-autophagic mechanism

As HMGB1 is implicated in autophagy (Tang *et al*, 2010, 2011), we performed electron-microscopic analyses expecting to detect changes of autophagy in Purkinje cells. However, we could not find any evidence to support an increase of autophagic vacuoles or of mitophagy in the cell bodies (Supplementary Fig S4D and E) and synapses (Supplementary Fig S4F) of Atxn1-KI mice although we found that the mitochondrial electron density was obviously decreased in Atxn1-KI mice (Supplementary Fig S4D). The interaction of HMGB1 and Beclin1, which was increased in the autophagy cellular model (Tang *et al*, 2010), was not also confirmed in our immunoprecipitation experiments with brain samples (Supplementary Fig S4G).

**Figure 3:**
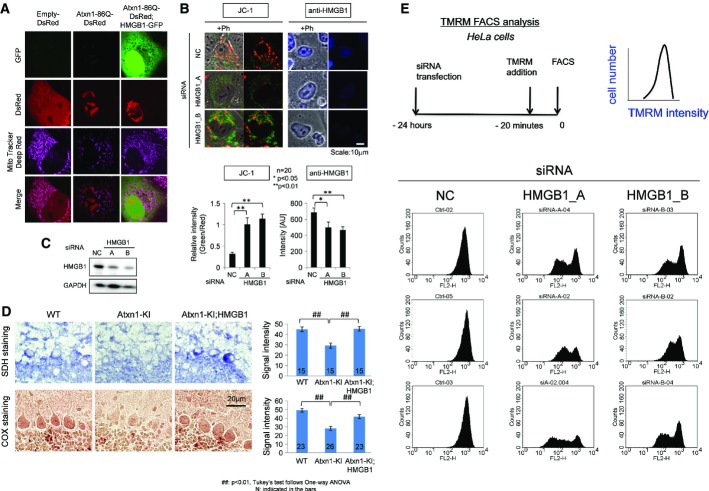
HMGB1 restores mitochondrial functions The accumulation of MitoTracker Deep Red, a far red-fluorescent dye indicator of the mitochondrial membrane potential, in the mitochondria of mutant Atxn1(86Q)-DsRed-transfected HeLa cells was reduced by co-expression of HMGB1-GFP.A knock-down of HMGB1 by two types of HMGB1-siRNA (HMGB1_A, HMGB1_B) decreased the number of mitochondria with the normal membrane potential (stained red with JC-1) and increased the number of mitochondria with an abnormal membrane potential (stained green with JC-1). NC: negative control siRNA. Right panels show HMGB1 signals in the transfected cells.Expression levels of HMGB1 in the transfected cells used in (B) were confirmed by Western blot analysis.Mitochondrial enzyme histochemical analysis revealed reduction of succinate dehydrogenase (SDH) and cytochrome oxidase (COX) activity in Purkinje cells of Atxn1-KI mice. The reduced activity of these enzymes was restored in Atxn1-KI;HMGB1 mice.FACS analysis with the well-characterized potentiometric fluorescent dye tetramethylrhodamine methyl ester (TMRM) to quantify the changes of the mitochondrial membrane potential and mitochondrial permeability transition induced by HMGB1. The upper left panel shows experimental procedure, and the upper right panel shows parameters in the following graphs. Non-specific negative control siRNA did not affect TMRM signals, whereas siRNA-A and siRNA-B against HMGB1 substantially reduced a part of transfected HeLa cells. The siRNAs that were used for this analysis were similar to those in Supplementary Fig S8, where suppression of HMGB1 by these siRNAs was confirmed. The results from three sets of independent transfection experiments indicated that the mitochondrial membrane potential and mitochondrial permeability transition were changed by the deficiency in HMGB1. Source data are available online for this figure.

However, we found several findings to suggest functional impairment of mitochondria by mutant Atxn1. First, we found that mutant Atxn1 impaired mitochondrial dynamics. Mitochondrial fission and fusion were evaluated by live imaging (Jendrach *et al*, 2005) in HeLa cells (Supplementary Videos S1, S2 and S3). Compared with HeLa cells expressing DsRed (Supplementary Video S1), fission and fusion frequencies were decreased in HeLa cells expressing mutant Atxn1-DsRed (Supplementary Video S2), and the decrease was rescued by co-expression of HMGB1-EGFP (Supplementary Fig S3H, Supplementary Video S3). Second, analysis of membrane potential by MitoTracker Deep Red revealed reduced mitochondrial membrane potential in HeLa cells expressing mutant Atxn1 and the recovery by co-expression of HMGB1-GFP (Fig3A). The change of mitochondrial membrane potential by HMGB1 was further tested with JC-1 (5,5′,6,6′-tetrachloro-1,1′,3,3′-tetraethylbenzimidazolylcarbocyanine iodide), a more direct indicator of in the mitochondrial membrane potential (ΔΨm) and siRNA against HMGB1 (Fig3B). As expected, transfection of two types of siRNAs changed the colour of JC-1 from red to green (Fig3B) and suppressed HMGB1 protein (Fig3C) in siRNA-transfected cells. Third, enzyme histochemistry revealed reduced activities of succinate dehydrogenase (SDH) and cytochrome c oxidase (CCO) activities in Purkinje cells of Atxn1-KI mice and their recovery in Atxn1-KI;HMGB1 mice (Fig3D). Fourth, HMGB1 knock-down decreased TMRM (tetramethylrhodamine methyl ester) intensity in FACS analysis of HeLa cells by transiently expressing HMGB1-siRNA (Fig3E).

These results collectively suggested that mutant Atxn1 damaged mitochondria via intrinsic mitochondrial function(s) related to HMGB1. Therefore, we next sought another hypothesis to explain the effect of mutant Atxn1 on mitochondria.

### HMGB1 contributes to mitochondrial DNA quality control

**Figure 4:**
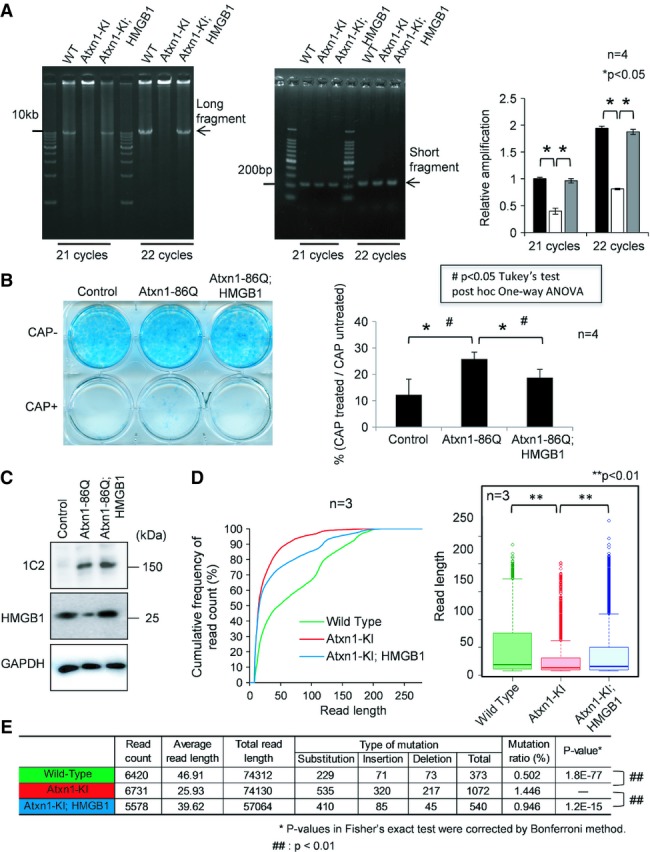
HMGB1 reverses mitochondrial DNA damage caused by mutant Atxn1 The mitochondrial DNA amplification assay with cerebellar tissue revealed that mitochondrial DNA damage was enhanced in mutant Atxn1-KI mice but reversed in the double-transgenic mice. The number of long cDNA fragments decreases when multiple DNA breaks occur between the primers, while short fragments are more easily amplified. The ratio of the long fragment to the short fragment was decreased in mutant Atxn1-KI mice (right panel). The data are presented as mean ± SD. Statistical analysis involved Student's *t*-test.The chloramphenicol (CAP) resistance assay of HeLa cells transfected with a control vector (pDsRed), Atxn1-86Q expression vector (pDsRed-Atxn1-86Q) or Atxn1-86Q expression vector + HMGB1 expression vector (pCI-HMGB1). The results showed an increase in mitochondrial DNA damage in mutant Atxn1-expressing HeLa cells. The increase was abrogated by co-expression of HMGB1. The data are presented as mean ± SD. Statistical analysis involved Student's *t*-test; *P < 0.05.Expression levels of Atxn1 and HMGB1 in transiently transfected HeLa cells used for the CAP assay in (B). Atxn1-86Q was detected with an anti-1C2 antibody.Analysis of mitochondrial genomic DNA using a next-generation sequencer. The cumulative percentages of read counts are plotted against the read length (left panel). The read lengths in the mitochondrial genome are also presented with box plots (right panel). Both analyses indicate that the read length was disproportionally shifted to the shorter fraction in Atxn1-KI mice. The shortening of the read length was reversed in Atxn1-KI;HMGB1 mice. The shift of distribution was analysed statistically using Friedman's test (*P* = 2.19 × 10^−31^) followed by Wilcoxon rank-sum test. *P*-values were corrected using the Bonferroni method. The *P*-value was 3.24 × 10^−7^ in the comparison between Atxn1-KI mice and background mice (wild-type) and 1.84 × 10^−3^ between Atxn1-KI;HMGB1 mice and Atxn1-KI mice.Frequency of mutation in the mitochondrial genome of the three genotypes of mice. The changes of mutation frequency were assessed statistically by Fisher's exact test and the *post hoc* Bonferroni correction. Mutation frequency was increased in Atxn1-KI mice compared to background mice (wild-type; *P* = 3.24 × 10^−7^) but normalized in Atxn1-KI;HMGB1 mice compared to Atxn1-KI mice (*P* = 1.84 × 10^−3^). Source data are available online for this figure.

From the localization of HMGB1 in the mitochondria matrix (Fig2B) and deprivation of HMGB1 from mitochondria by mutant Atxn1 (Fig2A and Supplementary Fig S4A and B), we speculated that HMGB1 might be relevant to the architectural control of mitochondria DNA. If this is the case, mitochondrial DNA damage repair should be affected. To address this question, we first investigated the ratio of long and short cDNA amplification from mitochondrial DNA, which is commonly used for the quantitative analysis of mitochondrial DNA damage (Das *et al*, 2010) based on the fact that nicks in mitochondrial DNA disturb cDNA extension. As expected, mitochondrial DNA damage in the cerebellar cortex was increased in Atxn1-KI mice, but not in Atxn1-KI;HMGB1 mice (Fig4A). We also performed the chloramphenicol (CAP) resistance assay (Aamann *et al*, 2010) based on the fact that HeLa cells with mitochondrial DNA damage become resistant to CAP. Again, the number of chloramphenicol-resistant colonies that possessed mitochondrial DNA damage was increased in HeLa cells expressing mutant Atxn1, but the increase was rescued by co-expression of HMGB1 (Fig4B). Expression levels of mutant Atxn1 and HMGB1 were equal in Western blot analysis (Fig4C).

Next, we evaluated mitochondrial DNA damage by direct sequencing of mitochondrial DNA (Fig4D). The assay was based on the fact that length of read becomes shorter when the template DNA has a nick or a single-/double-strand break. We first separated mitochondrial fraction from cerebellar tissues of WT mice (background C57BL/6 mice), Atxn1-KI mice and Atxn1-KI;HMGB1 mice and extracted DNA. Using the samples as template, we performed direct DNA sequencing using next-generation sequencer (NGS). Each sample produced 5,000–6,000 reads. The read sequences were referenced to mitochondrial genome sequence database, and only the reads that matched to the mitochondrial genome sequence were selected for further analysis, and the reads matched to nuclear genome were excluded from the next analysis. We made histograms from the results of read frequency and read length in three genotypes of mice. The distribution of read length was shifted to the shorter range in Atxn1-KI mice, while the shift was recovered in Atxn1-KI;HMGB1 mice (Fig4D). The shift and recovery was statistically confirmed using Friedman test with *post hoc* Wilcoxon rank-sum test. The difference was definite even with additional Bonferroni correction. Finally, NGS analysis revealed various types of mutations were actually increased in mitochondrial genome of the cerebellar tissues from Atxn1-KI mice, and they were recovered in Atxn1-KI;HMGB1 mice (Fig4E). These results directly indicated that mitochondrial DNA damage was increased in the cerebellum of Atxn1-KI mice *in vivo*, and it was rescued by transgenic co-expression of HMGB1.

**Figure 5:**
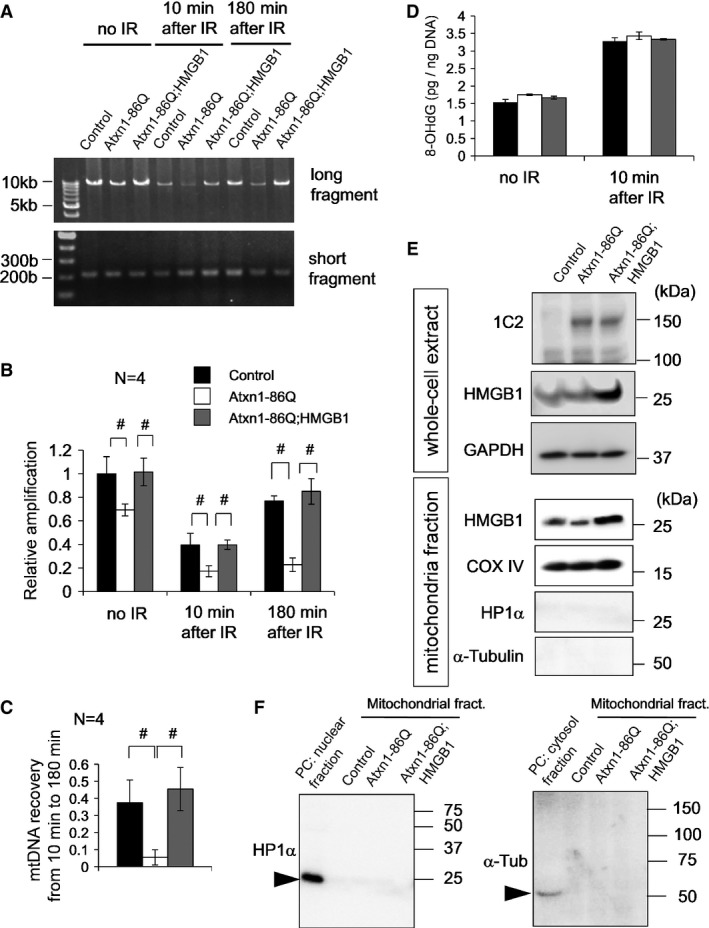
HMGB1 restores mitochondrial DNA repair after irradiation The mitochondrial DNA amplification assay with L929 cells at two time points (10 and 180 min) after X-ray irradiation to induce DNA damage. Amplification of the long and short fragments from the mitochondrial genome was performed at each time point after irradiation in mock-transfected, Atxn1-86Q-expressing and Atxn1-86Q-HMGB1-coexpressing L929 cells that had been transfected 48 h before irradiation. The control cells were transfected with the same amount of empty plasmids.Quantitative analysis of DNA damage at each time point using the ratio between short and long PCR fragments (long/short). Reduction of the ratio indicates enhanced DNA damage. At any time point, DNA damage was induced by mutant Atxn1 and attenuated by HMGB1. The data are presented as mean ± SD. ^#^*P* < 0.05 in one-way ANOVA followed by *post hoc* Tukey's HSD test.Reversal of DNA damage between minutes 10 and 180 was evaluated by subtraction of the values. Recovery was very small in Atxn1-expressing cells, but it was normalized by co-expression of HMGB1. The data are presented as mean ± SD. ^#^*P* < 0.05 in one-way ANOVA followed by *post hoc* Tukey's HSD test.The extent of DNA damage evaluated using 8-OHdG was equivalent among the three types of transfection.Increased levels of mutant Atxn1 and HMGB1 after transfection were tested in whole-cell extracts and in the mitochondrial fraction. Mutant Atxn1 was equally expressed after two types of transfection, and HMGB1 was upregulated in cells and mitochondria.To verify the results from the mitochondrial fraction, blots with anti-HP1α and α-tubulin antibodies were performed with a positive control. Source data are available online for this figure.

To evaluate the DNA damage repair activity in control, mutant Atxn1-expressing and mutant Atxn1/HMGB1-co-expressing L929 cells, the ratio of long and short cDNA amplification from mitochondrial DNA by PCR was calculated before and after X-ray irradiation (8 Gy) (Fig5A) according to the reported method (Zhou *et al*, 2012). At 10 min after X-ray irradiation, DNA damage still remained in three types of cells (Fig5B). From 10 to 180 min after X-ray irradiation, amplification of long fragment was improved in control cells and mutant Atxn1/HMGB1-co-expressing cells but not in mutant Atxn1-expressing cells (Fig5B). Subtraction of the 10-min values from the 180-min values (Fig5C) made the difference among three types of cells obvious in the recovery of mitochondrial DNA damage. HMGB1 clearly promoted the recovery from mitochondrial DNA damage (Fig5C). The extents of DNA damage induced by X-ray irradiation at the initial time point were similar in three types of cells (Fig5D), judging from quantification of 8-hydroxydeoxyguanosine (8-OHdG). In addition, expression levels of mutant Atxn1 were similar in mutant Atxn1-expressing cells and mutant Atxn1/HMGB1-co-expressing cells (Fig5E). HMGB1 was increased in mitochondrial fraction of Atxn1/HMGB1 co-expressing cells (Fig5E) without contamination of nuclear or cytosol fractions (Fig5F).

The essential role of HMGB1 in mitochondrial DNA damage repair was further tested by siRNA-mediated knock-down experiments. In the CAP resistance assay performed in parallel with JC-1 experiment (Fig3B) using the same siRNAs against HMGB1 (Supplementary Fig S4I), deficiency of HMGB1 (Supplementary Fig S4K) led directly to an increase in mitochondrial DNA damage. Consistently, shRNA-mediated knock-down of HMGB1 reduced the frequency of mitochondrial fission/fusion in HeLa cells (Supplementary Fig S4J). Together, these data support that HMGB1 contributes to mitochondrial DNA quality control and homeostasis.

### HMGB1 directly interacts with and repairs damaged mitochondria DNA

**Figure 6:**
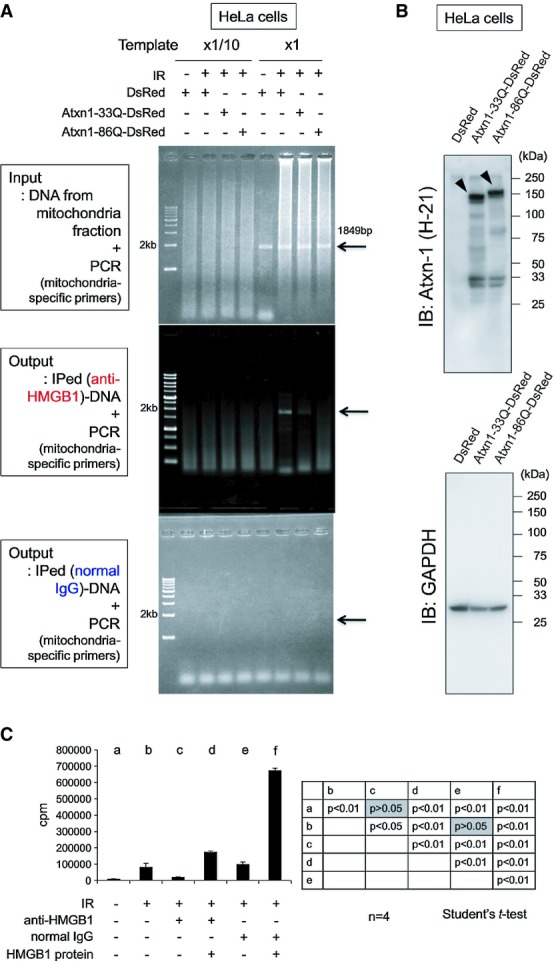
HMGB1 binds to damaged mitochondria and enhances DNA repair after irradiation Binding of HMGB1 to mitochondrial DNA was evaluated by the chromatin immunoprecipitation (ChIP) assay. The mitochondrial fraction was prepared from HeLa cells expressing pDsRed, pDsRed-Atxn1-33Q or pDsRed-Atxn1-86Q using the Mitochondria Isolation Kit (Thermo Fisher Scientific, IL, USA). Before or after immunoprecipitation with anti-HMGB1 antibody, a mitochondrial DNA fragment (1,849 bp) that bound to HMGB1 was amplified by PCR with the mitochondrial DNA-specific primers. The expected size of the PCR product (1,849 bp) was amplified almost equally from three samples before immunoprecipitation (Input, upper panel). In the samples subjected to immunoprecipitation with an anti-HMGB1 antibody (Output, middle panel), the PCR product was upregulated after the irradiation, indicating that a larger amount of HMGB1 protein binds to mitochondrial DNA after irradiation. The enhanced interaction between HMGB1 and the mitochondrial genome after irradiation was inhibited by Atxn1, especially by Atxn1-86Q. Normal IgG did not co-precipitate mitochondrial DNA (Output, lower panel).Expression levels of DsRed, Atxn1-33Q-DsRed and Atxn1-86Q-DsRed from the experiment in (A) were analysed by Western blotting.Quantitative analysis of mitochondrial DNA damage repair after irradiation. In the mitochondrial fraction prepared from HeLa cells after DNA damage by X-rays (8 Gy), we quantified incorporation of radioisotope-labelled nucleotides into damaged mitochondrial DNA during DNA damage repair. DNA repair in mitochondria was suppressed by addition of an anti-HMGB1 antibody but restored by recombinant HMGB1. Source data are available online for this figure.

To test direct involvement of HMGB1 in mitochondrial DNA repair, we performed several lines of experiments. First, we performed ChIP analysis with anti-HMGB1 antibody for mitochondrial DNA (Fig6A). A DNA fragment of 1,849 bp was amplified with mitochondria-specific primers from purified mitochondrial fraction of HeLa cells expressing DsRed, Atxn1-33Q-DsRed or Atxn1-86Q-DsRed. The specific band amplified from mitochondrial DNA co-precipitated with HMGB1 was obviously increased after irradiation (Fig6A, middle panel). Interestingly, the band was decreased in the presence of Atxn1 especially by mutant form expression. Expression levels of Atxn1-33Q-DsRed or Atxn1-86Q-DsRed were almost equivalent (Fig6B).

Second, to evaluate the amount of mitochondrial DNA repair, we quantified incorporation of radioactive nucleotide into DNA in mitochondrial fraction of HeLa cells after X-ray irradiation (8 Gy) (Fig6C). Damaged mitochondria was pretreated with anti-HMGB1 antibody or added with recombinant HMGB1 protein to test the effect of endogenous and exogenous HMGB1 protein on mitochondrial DNA damage repair. As expected, anti-HMGB1 antibody but not normal IgG inhibited mitochondrial DNA damage repair. HMGB1 recombinant protein recovered the inhibition by the antibody, and it also substantially enhanced mitochondrial DNA repair in the absence of anti-HMGB1 antibody (Fig6C). All these results indicated that HMGB1 protein directly interacts with and promotes repair of mitochondrial DNA.

### HMGB1 contributes to mitochondrial DNA repair *in vivo*

**Figure 7:**
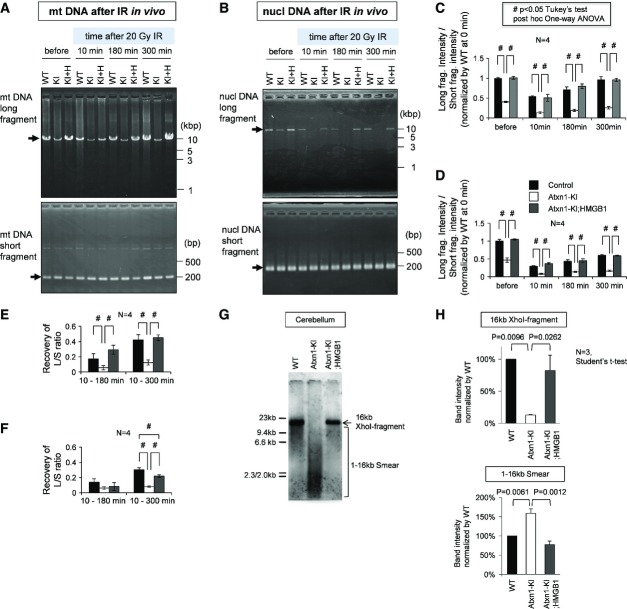
*In vivo* repair of mitochondrial DNA damage by HMGB1 Mitochondrial DNA damage repair was evaluated *in vivo* in mice subjected to X-ray irradiation (20 Gy) under anaesthesia. Cerebellar tissues were collected 10, 180 and 300 min after irradiation. The mitochondrial DNA amplification assay in the cerebellar tissue obtained from wild-type (WT), Atxn1-KI (KI) and Atxn1-KI;HMGB1-Tg double-transgenic (KI+H) mice. Amplification of the long and short fragments from the mitochondrial genome was performed at each time point after irradiation.Quantitative analysis of mitochondrial DNA damage at each time point using the ratio between short and long PCR fragments (long/short). Reduction of the ratio indicates enhanced DNA damage. The data are presented as mean ± SD. ^#^*P* < 0.05 in one-way ANOVA followed by *post hoc* Tukey's test.Reversal of mitochondrial DNA damage from 10 min to 180 min or 300 min was evaluated by subtraction of the values. The data are presented as mean ± SD. ^#^*P* < 0.05 in one-way ANOVA followed by *post hoc* Tukey's test.The nuclear DNA amplification assay in the cerebellar tissue obtained from wild-type (WT), Atxn1-KI (KI) and Atxn1-KI;HMGB1-Tg double-transgenic (KI+H) mice. Amplification of the long and short fragments from the nuclear genome was performed at each time point after the irradiation; ^#^*P* < 0.05 in one-way ANOVA followed by *post hoc* Tukey's test.Quantitative analysis of nuclear DNA damage at each time point using the ratio between short and long PCR fragments (long/short). A reduction in the ratio indicates enhanced DNA damage. The data are presented as mean ± SD. ^#^*P* < 0.05 in one-way ANOVA followed by *post hoc* Tukey's test.Reversal of nuclear DNA damage from 10 to 180 min or 300 min was evaluated by subtraction of the values. The data are presented as mean ± SD. ^#^*P* < 0.05 in one-way ANOVA followed by *post hoc* Tukey's test.Southern blot analysis of mitochondrial genomic DNA prepared from wild-type (WT), Atxn1-KI or Atxn1-KI;HMGB1-Tg double-transgenic mice. Mitochondrial DNA copy numbers were largely similar among the genotypes, although quality of the mitochondrial DNA was remarkably different.Signal intensity of a 16-kb band corresponding to the intact mitochondrial genome and those of a DNA smear (< 16 kb) corresponding to a degraded mitochondrial genome were quantified using ImageQuant LAS500 (GE Healthcare Life Sciences, Little Chalfont, UK) with ImageQuant TL software. Source data are available online for this figure.

To confirm that HMGB1 actually contributes to repair of mitochondrial DNA damage *in vivo*, we analysed reversal of mitochondrial DNA damage in three genotypes of mice subjected to X-ray irradiation (20 Gy) under anaesthesia. Simultaneously, we compared the extent of impairment in the DNA damage repair function between the mitochondria and nucleus. Mitochondrial and nuclear DNA amplification assays were performed in parallel using the cerebellar tissue obtained from wild-type, Atxn1-KI and Atxn1-KI;HMGB1 mice before irradiation and 10, 180 and 300 min after irradiation (Fig7A and B). Quantitative analysis of the ratio of long/short PCR fragments reconfirmed the increased DNA damage in both mitochondrial and nuclear genomes (Fig7C and D). The recovery of the long/short PCR fragments ratio was remarkably retarded in both mitochondrial and nuclear genomes in Atxn1-KI mice from 10 to 300 min (Fig7C and D). Subtraction of the ratio value at 10 min from that at 180 or 300 min further supported the delay of recovery in Atxn1-KI mice and the normalization in Atxn1-KI;HMGB1 mice (Fig7E and F). A minor difference was that the rescue of DNA damage repair was significant in mitochondria but not in the nucleus from 10 to 180 min (Fig7E and F).

Moreover, Southern blot analysis of mitochondrial genome DNA confirmed the difference in DNA damage in the default state of wild-type, Atxn1-KI and Atxn1-KI;HMGB1 mice at 13 weeks of age. Mitochondrial DNA copy numbers were largely similar across genotypes, while mitochondrial DNA was damaged substantially in Atxn1-KI mice, but not in Atxn1-KI;HMGB1 mice (Fig7G). Quantification of signal intensity of the 16-kb band corresponding to the intact mitochondrial genome and of the DNA smear (1–16 kb) by ImageQuant LAS500 confirmed the difference among three genotypes of mice (Fig7H).

### HMGB1 supplementation improves gene expression profiles

Although the direct role of HMGB1 in mitochondrial DNA damage repair was strongly suggested, the indirect role through transcriptional regulation might be possible. Therefore, we decided to investigate changes of gene expression profile by mutant Atxn1 and HMGB1. First, Purkinje cells were purely isolated from frozen tissue sections using laser dissection (Supplementary Fig S5A). RNA was extracted from dissected Purkinje cells, amplified by PCR and submitted to microarray analysis. The gene expression profiles in wild-type, Atxn1-KI and Atxn1-KI;HMGB1 mice (*N* = 3 for each mouse model) revealed that 2.8% of the genes were significantly decreased and 3.1% of genes were significantly increased in Atxn1-KI mice in comparison with background C57BL/6 mice (Supplementary Fig S5B). Among the decreased and increased genes, 18.2 and 24.3% of the genes, respectively, were normalized in Atxn1-KI;HMGB1 mice (Supplementary Fig S5B). The recovery ratios by HMGB1 were disproportionately high, suggesting another critical role of HMGB1 in SCA1 pathology. The regulated genes (Supplementary Table S1) were partially different from the genes obtained previously using a homologous approach (Crespo-Barreto *et al*, 2010). The discrepancy might be due to sample differences: the previous study used whole cerebellar tissues for RNA extraction.

We analysed the gene expression profiles using the PANTHER (Protein ANalysis THrough Evolutionary Relationships) Classification System (http://www.pantherdb.org/tools/genexAnalysis.jsp). The categorization of changed genes in Atxn1-KI mice and rescued genes in Atxn1-KI;HMGB1 mice suggested only that “metabolic process” and “primary metabolic process” genes were dominant (Supplementary Fig S5C). However, genes in these categories (Supplementary Table S2) were actually heterogeneous and the survey did not identify a specific function that explained the impact of mutant Atxn1 or HMGB1. Moreover, we performed gene ontology annotation enrichment analysis. The analysis selected catabolic genes rather than metabolic genes (Supplementary Table S3) because categorization and included genes were different from PANTHER, supporting that these analyses did not identify a specific function.

### HMGB1 improves the expression of some mitochondrial DNA repair genes

Because PATHER and ontology annotation enrichment analysis were uninformative, we sought to identify candidate genes mediating the effect of HMGB1 on the SCA1 pathology from expression patterns correlated with the worsening and rescue of mouse phenotypes. Initially, we surveyed mitochondrial genome-encoded genes changed in the SCA1 pathology. Some genes, such as NADH dehydrogenase 4, 5 and 6, were suppressed in Atxn1-KI mice, but not in Atxn1-KI;HMGB1 mice (Supplementary Fig S6A). Other mitochondrial genes, such as Cox1 and cytochrome b, changed in a similar manner, though the extent of change was not significant. These results, together with the DNA repair function of HMGB1 (Figs4 and 5, Supplementary Fig S4), might be relevant to the mitochondrial membrane potential abnormality (Fig3A and B).

Among the nuclear genome-encoded genes whose expression was rescued in Atxn1-KI;HMGB1 mice, many genes that contribute to DNA damage repair were included, such as frataxin (FXN), apurinic/apyrimidinic endonuclease (Apex1), ataxia telangiectasia mutated (ATM), xeroderma pigmentosum complementation group C (XPC) and replication protein A1 (RpA1) (Supplementary Fig S6A). Interestingly, FXN and Apex1 were decreased in Atxn1-KI mice but recovered in Atxn1-KI;HMGB1 mice, while ATM, XPC and RpA1 showed a reversed pattern. Importantly, base excision repair (BER) and mismatch repair (MMR), in which these genes are involved, contribute to the maintenance of mitochondrial genome (Kazak *et al*, 2012).

It is of note that RpA1 and BRCA1 were found to be relevant to DNA double-strand break repair in SCA1 from *Drosophila* genetic screening (Barclay *et al*, 2014). RpA1 and BRCA1 were also identified as the targets of ATM (Matsuoka *et al*, 2007). The recovery of these genes (Supplementary Fig S6A and B) might indirectly rescue the impaired DNA damage repair in Atxn1-KI mice.

### HMGB1 affects expression of some synapse-related genes

Screening of synapse-related genes revealed that cerebellin-1 (Cbl1), disabled-1 (Dab1) and talin-2 (Tln2) were decreased in Atxn1-KI mice, but not in Atxn1-KI;HMGB1 mice (Supplementary Fig S6B). Cbln1 is a secretory protein necessary for dendritic spine integrity and maturation (Kusnoor *et al*, 2010), and Tln2 is a critical molecule that controls spine morphology and maturation through the actin network (Terry-Lorenzo *et al*, 2005). Dab1, a protein that binds to the intracellular domains of Reelin receptors, mediates Reelin signalling (Hiesberger *et al*, 1999), which regulates neuronal migration (Franco *et al*, 2011; Jossin & Cooper, 2011; Sekine *et al*, 2011).

Therefore, their reduction might be related to the decreased thickness of the molecular layer in Atxn1-KI mice and the recovery of Atxn1-KI;HMGB1 mice. Consistent with this idea, using two-photon microscopy, we found abnormally short and less branched dendrites in the Purkinje cells of Atxn1-KI mice; the dendrites were rescued in Atxn1-KI;HMGB1 mice (Supplementary Fig S7A and B). On the other hand, some Purkinje cells in Atxn1-KI mice showed numerous fine and short dendrites reminiscent of a loss of pruning or abnormal sprouting; such changes were not found in Atxn1-KI;HMGB1 mice (Supplementary Fig S7A and B). Similar findings from immunohistochemistry of mutant Atxn1-transgenic mice with anti-calbindin antibody were previously reported (Clark *et al*, 1997). Further analysis with two-photon microscopy revealed spines of abnormally high density and increased length in the Purkinje cells of Atxn1-KI mice (Supplementary Fig S7C and D). The spine volume was increased, but the diameter was not changed. In addition, the ratio among the three subtypes was not changed (Supplementary Fig S7C and D). The phenotype of abnormally long and dense spines was rescued in Atxn1-KI;HMGB1 mice (Supplementary Fig S7C and D).

Screening of autophagy-related genes revealed that Atg10 and Pik3cb showed the decrease–increase pattern (Supplementary Fig S6C) although macroautophagy was not activated in Purkinje cells (Supplementary Fig S4). Collectively, synaptic dysfunction based on the transcriptional dysregulation in Purkinje cells could be the third mechanism dependent on HMGB1, in addition to nuclear and mitochondrial DNA damage repair.

### AAV vector expressing HMGB1 rescues SCA1 model mouse symptoms

**Figure 8:**
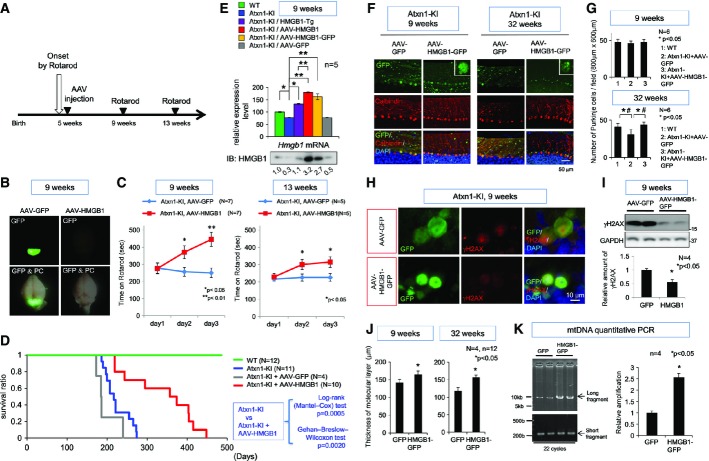
AAV-HMGB1 reverses the pathological phenotypes and DNA damage of mutant Atxn1-KI mice *in vivo* The experimental protocol of the AAV injection.Injection of AAV-GFP to the cerebellar surface (at 5 weeks) efficiently transfected cerebellar tissues with AAV. Fluorescence was detected directly by illumination under a fluorescence stereoscopic microscope at 4 weeks after the injection. The AAV vector for the expression of untagged HMGB1 (AAV-HMGB1) was similarly injected at 5 weeks (*n* = 12). GFP expression was not detected because HMGB1 was not tagged.The rotarod performance at 9 weeks (4 weeks after injection) and at 13 weeks (8 weeks after injection) revealed improvement of motor function by AAV-HMGB1 in Atxn1-KI mice after the onset of symptoms. The data from days 1 to 3 are presented as mean ± SD. Statistical analysis involved Student's *t*-test.Survival curves of Atxn1-KI mice (blue line), AAV-HMGB1-injected Atxn1-KI mice (red line), AAV-GFP-injected Atxn1-KI mice (grey line) and wild-type animals (green line).Relative expression levels of HMGB1 mRNA at 9 weeks were evaluated by quantitative PCR with mRNA prepared from cerebellar tissues of Atxn1-KI mice or AAV-HMGB1-injected Atxn1-KI mice (injected at 5 weeks); *P < 0.05, **P < 0.01.Expression of HMGB1-GFP and GFP, which had been delivered by intrathecal injection of AAV at 5 weeks, was detected in Purkinje cells at 9 and 32 weeks. The GFP signals were directly acquired using fluorescence microscopy without immunostaining (upper panels). Meanwhile, Purkinje cell numbers were evaluated by immunostaining with an anti-calbindin antibody (lower panels). The decrease in the Purkinje cell number in Atxn1-KI mice was reversed by AAV-HMGB1-GFP even at 32 weeks. Dendrites of Purkinje cells in the molecular cell layer were also normalized morphologically at 9 and 32 weeks.The graphs show quantitative analyses of Purkinje cells of AAV-HMGB1-GFP- or AAV-GFP-injected Atxn1-KI mice at 9 and 32 weeks (mean ± SD, *P < 0.05 both in Student's *t*-test; ^#^*P* < 0.05 in one-way ANOVA followed by *post hoc* Tukey's HSD test).Immunohistochemical analysis of the cerebellum of Atxn1-KI mice injected by AAV-HMGB1-GFP revealed that HMGB1 reduced nuclear DNA damage in Purkinje cells. GFP signals were detected directly without immunostaining. The γH2AX signals were visualized with an anti-γH2AX antibody. The γH2AX signals were reduced in the Purkinje cells of Atxn1-KI mice injected with AAV-HMGB1-GFP compared to the signals in Purkinje cells of AAV-GFP-injected Atxn1-KI mice.Western blotting of the cerebellar tissues also showed that γH2AX was decreased in mutant Atxn1-KI mice injected with AAV-HMGB1-GFP. Quantitative analysis of the bands (mean ± SD) confirmed statistical significance of the difference (Student's *t*-test, *P* < 0.05).Infection of AAV-HMGB1-GFP restored the thickness of the molecular layer in mutant Atxn1-KI mice at 9 and 32 weeks. The mean thickness was quantified from 12 visual fields, and the means ± SD were calculated for four mice in the AAV-GFP-injected (GFP) or AAV-HMGB1-GFP-injected (HMGB1) group; *P < 0.05 in Student's *t*-test.Infection with AAV-HMGB1-GFP reduced mitochondrial DNA damage. The increased proportion of the long PCR fragment amplified from mitochondrial DNA revealed AAV-HMGB1-GFP-induced amelioration of mitochondrial DNA damage. The ratio of the short and long fragments was calculated from their band intensity after amplification in 21 PCR cycles. The data are presented as mean ± SD; *P < 0.05 in Student's *t*-test (*n* = 4). Source data are available online for this figure.

Finally, we tested whether virus-mediated gene delivery of HMGB1 ameliorates the symptoms. For this purpose, we used adeno-associated virus type 1 (AAV1), which can be delivered into the brain directly (Iwamoto *et al*, 2009) or indirectly by peripheral vein injection (our unpublished result). We constructed an AAV1 vector with a cytomegalovirus (CMV) enhancer/promoter to express HMGB1 or HMGB1-GFP, in which expression cassette of AAV3 between ITRs was packaged into capsid proteins of AAV1 (Supplementary Fig S8A). Given that construction of the vector was complex, we simply called our vector as AAV. As a control, we used AAV-GFP. The time point of injection was set at 5 weeks when Atxn1-KI mice already show deterioration in motor activity with rotarod (Fig1D). We observed the symptoms at 4 and 8 weeks after injection (9 and 13 weeks of age) (Fig8A).

We first confirmed that cerebellar surface injection of AAV-GFP induced a high level of gene expression in the cerebellum after 4 weeks of infection when the whole brain was illuminated by UV light (Fig8B). Under these conditions, Atxn1-KI mice infected with AAV-HMGB1 showed greater improvement of motor activity than AAV-GFP-infected Atxn1-KI mice (Fig8C). Although GFP-fusion protein might have toxicity, we also performed the similar experiment with AAV-HMGB1-GFP. Infection of AAV-HMGB1-GFP also induced a similar level of expression in the cerebellum (Supplementary Fig S8B). Remarkable improvement in motor activity was reproduced by infection of AAV1-HMGB1-GFP (Supplementary Fig S8C) when we similarly injected AAV-HMGB1-GFP at 5 weeks of age and tested the motor function of Atxn1-KI mice from 9 to 12 weeks of age. Moreover, we found a surprising effect on the lifespan both by AAV and HMGB1 (Fig8D). The mean lifespan was elongated from 217 days in Atxn1-KI mice to 365.5 days, and the maximum lifespan was increased from 274 to 448 days (Fig8D). We confirmed that AAV-HMGB1-GFP also elongated the lifespan of Atxn1-KI mice (Supplementary Fig S8D) while the effect was smaller than AAV-HMGB1 probably due to an unfavourable effect of GFP-fusion. Expression of GFP alone by AAV-GFP seemed toxic from the slightly shortened lifespan (Fig8D and Supplementary Fig S8D). The effect of AAV-HMGB1 was far larger than that of the transgenic HMGB1 expression (Fig1F). One reason could be that mRNA expression level of HMGB1 in the cerebellum of AAV1-injected mice was higher than that in Atxn1-KI;HMGB1 mice (Fig8E).

An encouraging result for possible human application in the future was that the expression of HMGB1-GFP sustained at least until 32 weeks (27 weeks after a single injection) and the reduction of Purkinje cells in Atxn1-KI mice was obviously rescued at 32 weeks (Fig8F and G). Interestingly, HMGB1-GFP was located dominantly in the cytoplasm at 32 weeks, suggesting that cytoplasmic function of HMGB1 might dominantly contribute to the recovery in the phenotype and lifespan of Atxn1-KI mice. As shown in Supplementary Fig S2A and B, we did not observe inflammatory responses in the cerebellum of Atxn1-KI mice infected by AAV-HMGB1 (Supplementary Fig S2A and B).

Accordingly, histological examination revealed improvement in the Purkinje cell dendrites in the molecular cell layer (Fig8F). Consistently, nuclear DNA damage was remarkably improved when estimated by immunohistochemistry and Western blot of γH2AX (Fig8H and I). The thickness of the molecular layer was obviously increased by AAV-HMGB1-GFP infection at 32 weeks (Fig8J) but even at 9 weeks increased statistically (Fig8J), consistent with the morphological improvement of Purkinje cell dendrites stained by calbindin (Fig8F). Mitochondrial DNA damage was also improved by HMGB1 when assessed by the ratio of the long and short DNA fragments amplified from mitochondrial genome DNA (Fig8K). These data collectively suggested that AAV-mediated delivery of HMGB1 could ameliorate both of the nuclear and mitochondrial pathologies in SCA1 and thereby could rescue the clinical symptom and lifespan.

Taken together, our results suggest that AAV-mediated delivery of HMGB1 is a promising candidate method to treat human SCA1 patients in the future.

## Discussion

Our previous study using a proteomics approach identified molecules in the nuclear soluble fraction that changed with SCA1 pathology (Qi *et al*, 2007). Of these molecules, HMGB1 was most consistently decreased in vulnerable neurons. In this study, we first showed that HMGB1 supplementation is effective in an SCA1 mouse model (Fig1, Supplementary Figs S1 and S2) and that the therapeutic effect by AAV-HMGB1 was expected even after the onset of symptoms (Fig8, Supplementary Fig S8). In addition, results in this study indicated that HMGB1 exists in mitochondrial membrane (Fig2); HMGB1 binds to damaged mitochondrial DNA (Fig6A); HMGB1 enhances damage repair of mitochondrial DNA (Figs4, 5, 6 and 7); HMGB1 reduction or inhibition increased mitochondrial DNA damage (Fig6D and Supplementary Fig S4I). Expression of mutant Atxn1 reduces HMGB1 (Figs2A, 4 and 5, Supplementary Fig S4A and B) and inhibits HMGB1 for damage repair (Figs4, 5, 6 and 7). The relationship between HMGB1 and neurodegeneration has not been implicated previously from the standpoint of mitochondrial DNA damage repair: this is the first report to provide a new mechanistic hypothesis for the neurodegeneration of Purkinje cells in the pathology of SCA1. Simultaneously, this is the first study to elucidate a role for HMGB1 in mitochondrial DNA damage repair. However, since many molecular mechanisms are known to be involved in the SCA1 pathology, relative contribution of the mitochondrial DNA repair to the SCA1 pathology should be further investigated in the future.

It was hypothesized that mitochondrial DNA damage and repair contribute to neurodegeneration (Yang *et al*, 2008). One study suggested that base excision repair was impaired in the brain tissues of Alzheimer's disease patients via dysfunction of DNA glycosylases and DNA polymerase beta (Weissman *et al*, 2007). However, the experiment used human postmortem brain tissues that may have been subjected to various stresses that induce DNA damage, and the molecular pathways that link causative proteins to neurodegeneration via mitochondrial DNA damage repair are still unknown in many neurodegenerative diseases. Moreover, the involvement of a DNA architectural protein such as HMGB1 in mitochondrial DNA damage repair has not been reported as a neurodegenerative mechanism. On the other hand, recent results have begun to disclose the landscape of mitochondrial DNA damage repair systems (Kazak *et al*, 2012), including base excision repair, single-strand break repair, mismatch repair and possibly homologous recombination. Together with such knowledge, the findings obtained in this study will help us understand how mitochondrial DNA damage and repair contribute to neurodegeneration. In addition, this study revealed that a therapy targeting mitochondrial DNA damage repair works *in vivo*.

HMGB1 is a well-known regulator of nuclear DNA architecture, and it has been shown to participate in multiple types of nuclear DNA damage repair including mismatch repair (Yuan *et al*, 2004; Zhang *et al*, 2005), nucleotide excision repair (Lange *et al*, 2008, 2009), base excision repair (Prasad *et al*, 2007; Lange & Vasquez, 2009). HMGB1 also regulates the other types of DNA damage repair such as non-homologous end joining (Lange & Vasquez, 2009). HMGB1 conducted these repair mechanisms through binding to damaged DNA, alteration of DNA structure, interaction with repair enzymes or cofactors and chromatin remodelling (Liu *et al*, 2010).

In contrast, mitochondrial DNA damage repair by HMGB1 is a new finding in this study (Figs2, 3, 4, 5, 6 and 7, Supplementary Fig S4), and it added another component to the complex functions of HMGB1 that are dependent on its intracellular and extracellular localization (Supplementary Fig S9). It is mitochondrial quality control based on mitochondrial DNA damage repair and distinct from that based on autophagy (Supplementary Fig S9). Our ultrastructural analyses with mouse brain tissues and our previous *in vitro* experiments, in which we observed LC3-positive vacuoles when mutant Atxn1 was expressed, did not support a change in macroautophagy (Fujita *et al*, 2013). It might suggest that the DNA damage repair-based quality control is more important in SCA1.

From the aspect that HMGB1 is involved both nuclear and mitochondrial DNA damage repair, it might be possible that the two DNA repair systems compete for HMGB1. However, our results revealed reduction of HMGB1 in both fractions (Fig2A), indicating that impairment of DNA damage repair occurred in both organelles in parallel. Our data from *in vivo* irradiation suggested both mitochondrial and nuclear DNA damage repairs were similarly affected (Fig7A–F). However, despite of our results in SCA1 pathology, the possible competition between the two repair systems for HMGB1 under the physiological state (instead of pathological state under the SCA1 pathology) should be further examined in the future.

Expecting therapeutic effects, we performed two types of functional complementation of HMGB1 by transgenic expression and AAV-mediated expression. In transgenic expression of HMGB1, we detected multiple effects of HMGB1 on the phenotypes and pathology of Atxn1-KI mice. In particular, lifespan was elongated to 130% and improvement of motor activity sustained even after a single injection. More surprisingly, AAV-mediated expression of HMGB1 elongated the mean lifespan from 217 days in Atxn1-KI mice to 365.5 days (168%) and the maximum lifespan from 274 to 448 days (163%). These lifespan elongations are the best results to date. Motor functions are improved in both types of HMGB1 delivery. The therapeutic effect of HMGB1 was dose dependent judging from the larger effect of AAV1-mediated expression than that of transgenic expression.

The reason was not clear why a limited upregulation of HMGB1 in Atxn1-KI; HMGB1 mice was also effective. As discussed already, one possible explanation would be that HMGB1 is a master gene regulating multiple types of DNA damage repair. The second reason could be that HMGB1 also improves transcription of multiple genes related to various neuronal functions as shown in microarray results (Supplementary Fig S5A–C). Integrated beneficial effects of HMGB1 on multiple cellular functions (Supplementary Fig S9) might have recovered the phenotypes more than expected.

Meanwhile, it is also true that HMGB1 could not completely normalize the phenotypes or lifespan of Atxn1-KI mice. The insufficient upregulation of HMGB1 in Atxn1-KI;HMGB1 mice and the cerebellum-restrictive expression in AAV1-HMGB1-infected mice might be partially responsible. However, another reason is that other mechanisms also contribute to the SCA1 pathology. Certainly, the reciprocal interaction of Atxn1 with CIC/Capicua and RBM17 is important (Lam *et al*, 2006; Lim *et al*, 2008). Gene manipulation of CIC/Capicua elongated life span by nearly 30 days (at the maximum survival duration) in the same Atxn1-KI mice (Fryer *et al*, 2011). In this aspect, combining multiple therapeutic approaches based on multiple target molecules might have a larger therapeutic effect on the SCA1 phenotypes.

A concern for the therapeutic usage of HMGB1 is the induction of inflammation (Supplementary Fig S9): extracellular HMGB1 triggers inflammation by binding to TLR2/4 or RAGE (Park *et al*, 2006; Yu *et al*, 2006). However, transgenic expression of HMGB1 did not damage neurons morphologically from 9 to 32 weeks or lead to an abnormal phenotype until 55 weeks (Fig1D and F). Moreover, infection with AAV1-HMGB1 vector also ameliorated symptoms for at least for 8 weeks after injection. Since the binding of HMGB1 to TLR4 at the plasma membrane induces immediate inflammatory responses in multiple types of brain cells (Bianchi & Manfredi, 2007), 4 weeks of observation is sufficient to exclude such a side effect. Although our data excluded the harmful effects of an inflammatory response triggered by HMGB1, the possible side effects should better be re-evaluated with mammals, such as monkeys, before the clinical application of HMGB1 therapy to human patients.

Another possible side effect might occur specifically in cancer treatment. Inhibition of DNA damage repair is a mechanism used for anticancer therapeutics (Helleday *et al*, 2008). This in turn suggests that enhanced DNA damage repair activity might make cancer cells more resistant to treatment. However, the issue can be solved by restricting HMGB1 expression to neurons that need to be rescued. In addition, we did not find any cancer in pathological examination of Atxn1-KI;HMGB1 mice (*n* = 12) or AAV1-HMGB1-infected mice (*n* = 10). The final concern is HMGB1's broad effect on gene expression. Unexpected effects might occur in cells overexpressing HMGB1. This issue is analogous to iPS cells in which the gene expression pattern in cells differentiated from iPS cells is not exactly similar to that in expected cells. We are continuing to observe HMGB1-Tg mice during ageing. Our long-term follow-up of HMGB1-Tg mice over 1.5 years did not detect cancer or obvious neurological phenotypes (data not shown). Naturally, these concerns should be further investigated and addressed as part of the process towards the clinical application of AAV1-HMGB1 for SCA1 patients.

Finally, when these concerns are overcome, we might be able to consider combination therapy with the other types of DNA repair proteins. We previously reported that overexpression of Ku70, a critical DNA repair protein for non-homologous end joining, was effective for a mouse model of HD (Enokido *et al*, 2010). Although we found in a preliminary experiment that Ku70 was not effective for Atxn1-KI mice (Supplementary Fig S1H), Ku70 might be useful for the other types of neurodegeneration. Recently, we evaluated relative contributions of various DNA damage repair factors related to the SCA1 pathology by *Drosophila in vivo* screen combined with informatics and found that RpA1 and some other factors were effective for lifespan elongation in *Drosophila* SCA1 model (Barclay *et al*, 2014). These factors could be candidates for a combination therapy with HMGB1 for SCA1 and possibly for the other neurodegenerative disorders. As discussed already, combination therapy targeting on the other pathological domain together with DNA damage repair might be also effective.

In conclusion, we propose in this study a novel therapeutic approach targeting HMGB1 against the SCA1 pathology.

## Materials and Methods

### Generation of the HMGB1 transgenic mouse

To generate transgenic mice expressing HMGB1, a 1.9-kb sequence upstream of the rat neuron-specific enolase (NSE) gene (from *Rattus norvegicus*, chromosome 4, nucleotides 146320892 to 146318938), which was identified as the NSE enhancer/promoter (Forss-Petter *et al*, 1990), was subcloned from the genomic DNA of Brown Norway and Sprague Dawley rats and inserted into the pIRES-hrGFPII vector (Stratagene) by replacing the CMV promoter at *Spe*I and *Eco*RI sites. The full-length rat HMGB1 cDNA (BC_081839) was inserted downstream of the NSE enhancer/promoter in pIRES-hrGFPII, which also included an internal ribosome entry site (IRES) and humanized recombinant GFPII. To distinguish endogenous and transgenic HMGB1, a 3× FLAG tag sequence was added to the C-terminus of HMGB1 cDNA.

The plasmid was digested with *Asc*I and *Ssp*I, and the resultant 4.4-kb fragment was injected into fertilized mouse oocytes of C57BL/6 mice (Japan SLC, Hamamatsu, Japan). HMGB1 transgenic mice were crossed with mutant ataxin-1 KI mice to generate HMGB1-overexpressing mutant ataxin-1 KI mice. Littermate mice were used as a control. Genomic DNA was extracted from the tails, and genotyping was performed by PCR with the primers 5′-AGTCTGCAGTCCTCGAGGAA-3′ (forward) and 5′-GTCTTCCACCTCTCTGAGCA-3′ (reverse; *Rattus norvegicus*, NSE, chromosome 4, nt 146318954–146318938 and *Rattus norvegicus*, HMGB1, chromosome 12, nt 7085873–7085724), which amplify the sequence at the junction of the NSE promoter and the HMGB1 gene. Amplification was performed using Ex-Taq (Takara). The conditions were as follows: 35 cycles of 94°C for 30 s (denaturation), 60°C for 30 s (annealing) and 72°C for 30 s (extension). The size of the product was 220 bp. Protein expression from the transgene was confirmed by Western blotting using antibodies against anti-FLAG (dilution 1:3,000, cat. #F3165, Sigma-Aldrich, MI, USA).

### Western blot analysis

For Western blotting, brain tissues from mutant Atxn1 KI mice or littermate control mice were washed three times with ice-cold PBS and dissolved in lysis buffer containing 62.5 mM Tris–HCl pH 6.8, 2% (w/v) SDS, 2.5% (v/v) 2-mercaptoethanol and 5% (v/v) glycerol. Samples from cultured cells were prepared similarly. The protein concentration was quantified using the BCA method (Micro BCA Protein Assay Reagent Kit; Pierce Chemical, Rockford).

Primary and secondary antibodies were diluted for immunoblotting as follows: rabbit anti-HMGB1, 1:1,000 (ab18256, Abcam, Cambridge, UK); mouse anti-HMGB1, 1:1,000 (AHM0915, ATGen, Seongnam-si, Korea); rabbit anti-FLAG, 1:2,000 (F7425, Sigma, MI, USA); mouse anti-phospho-H2AX (γH2AX), 1:500 (Ser139, #05-636, Millipore, MA, USA); mouse anti-1C2, 1:1,000 (MAB1574, Millipore, MA, USA); rabbit anti-ubiquitin, 1:1,000 (Z0458, DAKO, Glostrup, Denmark); rabbit anti-HP1α, 1:1,000 (2623S, Millipore, MA, USA); rabbit anti-Cox IV, 1:1,000 (ab16056, Abcam, Cambridge, UK); mouse anti-α-tubulin, 1:1,000 (T6199, Sigma, MI, USA); mouse anti-GAPDH, 1:5,000 (MAB374, Millipore, MA, USA); and HRP-conjugated anti-mouse IgG and anti-rabbit IgG, 1:3,000 (NA931VS (mouse) and NA934VS (rabbit), GE Healthcare, NJ, USA). Antibodies were diluted in TBST with 5% skim milk.

### Immunohistochemical analysis

Mouse brains were fixed in 4% paraformaldehyde for 12–16 h. The paraffin-embedded mouse sections were deparaffinised, rehydrated and microwaved in 10 mM of citrate buffer, pH 6.0, at 120°C for 15 min. These sections were incubated sequentially with 1% skim milk for 30 min, with primary antibodies overnight at 4°C and finally with Alexa Fluor 488- and 594-labelled anti-IgGs (A21202 and A21206, Invitrogen, CA, USA) for 1 h at room temperature. The primary antibodies were diluted as follows: mouse anti-γH2AX antibody, 1:500 (Ser139, #05-636, Millipore, MA, USA); rabbit anti-53BP1, 1:5,000 (NB100-304, Novus Biologicals, CO, USA); rabbit anti-calbindin D-28K antibody, 1:200 (C2724, Sigma, MI, USA); mouse anti-calbindin D-28K antibody, 1:200 (C9848, Sigma, MI, USA); rabbit anti-HMGB1, 1:200 (ab18256, Abcam, Cambridge, UK); mouse anti-HMGB1, 1:100 (AHM0915, ATGen, Seongnam-si, Korea); mouse anti-FLAG, 1:500 (F3165, Sigma, MI, USA); rabbit anti-GFP, 1:100 (A6455, Invitrogen, CA, USA); rabbit anti-Cox IV, 1:1,000 (ab16056, Abcam, CA, USA); and a rabbit anti-ubiquitin antibody, 1:1,000 (Z0458, DAKO, Glostrup, Denmark).

Co-localization of HMGB1 and Cox IV was evaluated by calculating the area containing HMGB1 and Cox IV signals in Purkinje cells. Images were obtained by confocal microscopy and transferred to Adobe Photoshop. The areas of yellow colour (HMGB1^+^/Cox IV^+^) and red colour (Cox IV^+^) were selected in Photoshop and transferred to ImageJ for calculation of the merged area. The ratio between the yellow and red was used as the co-localization ratio.

### Acquisition of signal intensity

In Western blot analysis, LAS images were analysed with ImageJ (http://imagej.nih.gov/ij/), and the signal intensity levels were calculated. For agarose gel electrophoresis, images of ethidium bromide-stained gels placed on a UV transilluminator were taken with a digital camera (Print Graph, ATTO) and analysed in a similar fashion in ImageJ.

In immunohistochemical analysis, the signal intensity per cell was calculated as described previously (Qi *et al*, 2007) using fluorescence microscopy (Olympus IX-71) with MetaMorph software (Universal Imaging Corporation, Downingtown). Stained cells were selected at random, and the fluorescence signal intensity was recorded from more than 100 cells in at least 3 wells. To determine subcellular signal intensity levels, more than 10 regions of interest (ROI, 1 μm^2^) were selected at random in the nucleus or the cytoplasm of a single cell, and the mean value was used for the next step. Purkinje cell signals were obtained from more than 10 regions (1 μm^2^/each ROI), and the nuclear and cytoplasmic signals were compared among background C57BL/6 mice (WT), mutant Atxn1-KI mice (Atxn1-KI) and double-transgenic mice (Atxn1-KI;HMGB1; lower graph). More than 100 Purkinje cells were analysed. To subtract the background fluorescence intensity, we measured the fluorescence of 10 randomly selected non-cellular visual fields in each sample, and their mean value was subtracted from the fluorescence of the cells or subcellular regions on the same slide.

### Mouse behavioural tests

Mice were segregated by sex, housed at 2–5 per cage, provided with water and rodent chow and maintained in a 12-h/12-h light/dark cycle. All experiments were performed during the light phase (10:00–19:00 h) using male mice between 5 and 25 weeks of age. In the rotarod test, mice were placed on a rotating rod (3.5 rpm), and the rotating speed was linearly increased to 35 rpm in 300-s intervals and maintained at 35 rpm for 600 s (for 5- to 21-week-old mice). Mice were tested four times a day for three consecutive days. The mean latency to falling off the rotarod was recorded and used for subsequent analyses. Survival curves were analysed using the Kaplan–Meier method and log-rank test. Behavioural tests were performed with the investigator performing the test blinded to genotypes and treatments. A different researcher performed genotyping and mice were supplied at random to the investigator.

### Isolation of the mitochondrial fraction

The mitochondrial fraction was isolated using three methods. First, the mitochondrial fraction was prepared from HeLa cells (ATCC, VA, USA) or mouse brain tissues using the Mitochondria Isolation kit (Thermo Fisher Scientific, IL, USA).

Second, we performed isotonic homogenization and isolated the mitochondrial fraction by centrifugation from the mouse liver as described previously (Shimizu *et al*, 1998). In brief, mouse liver tissues were homogenized with a glass–Teflon Potter homogenizer in buffer consisting of 0.3 M mannitol, 10 mM potassium HEPES buffer pH 7.4, 0.2 mM EDTA pH 8.0 and 0.1% fatty acid-free BSA. After centrifugation at 2,500 × *g* for 10 min, mitochondria were isolated from the supernatant by centrifugation at 5,000 × *g* for 8 min and 10,000 × *g* for 5 min. The mitochondria were washed twice with this buffer without EDTA to which 5 mM potassium phosphate was added and then were resuspended in it.

Third, we isolated mitochondria using discontinuous Percoll density gradient centrifugation. Preparation of the mitochondrial fraction was performed using Nature Protocols (Method B) (Sims & Anderson, 2008) with minor modifications. Briefly, we dissected and obtained a half of the whole brain from mice (200–250 mg) and washed it with cold isolation buffer (10 mM Tris, 320 mM sucrose, 1 mM EDTA, pH 7.4). Brain tissue was minced into small pieces (∼1 mm^3^) and was rinsed by cold isolation buffer. The minced brain was mixed with 10× volume (vol/wt) of cold isolation buffer and homogenized by means of a 7-ml Dounce homogenizer using 4 strokes with the loose glass pestle and eight strokes with the tight pestle on ice. The homogenate was mixed with 1× volume of 24% Percoll in cold isolation buffer. We prepared discontinuous Percoll gradients consisting of 1.6 ml of 26% Percoll layered above 1.6 ml of 40% Percoll and then slowly layered 1.6 ml of the homogenate directly on Percoll gradients in tubes on ice. We centrifuged them in a fixed-angle rotor (TLA-110, Beckman Coulter) at 30,700 *g* at 4°C for 5 min and monitored three bands after centrifugation. We collected the third lower band and removed Percoll.

### Proteinase K resistance of the mitochondrial HMGB1 protein

The mitochondrial fraction isolated by Percoll density gradient centrifugation (3 μg) was digested by 5 μg/ml proteinase K for 60 min at 37°C in 10 mM Tris–HCl (pH 7.5) with 1 mM EDTA and loaded onto SDS–PAGE for Western blot analysis. Mitochondrial membrane was perforated by six cycles of freeze-thaw. Anti-HMGB1 (ab18256, Abcam, Cambridge, UK, 1:1,000), anti-Cox IV (ab16056, Abcam, Cambridge, UK, 1:1,000), anti-cytochrome c (SC-13156, Santa Cruz Biotechnology, TX, USA, 1:500), anti-TFAM (ab47517, Abcam, Cambridge, UK, 1:500) or anti-Tom20 antibody (SC-11415, Santa Cruz Biotechnology, TX, USA, 1:500) were used for Western blot analysis as a primary antibody. The secondary antibody was anti-rabbit IgG horseradish peroxidase (HRP)-conjugated antibody (NA934, GE, NJ, USA, 1:3,000).

### Plasmid construction

To construct pHMGB1-EGFP, rat HMGB1 cDNA (673 bp, *Rattus norvegicus*, high-mobility group box 1, NM_012963, nt 29–702) was amplified from the RNA of rat cortical neurons using primers 5′-CCGCTCGAGCTGTGCCTCGCGGAGGAA-3′ and 5′-GGAATTCGTTCATCATCATCATCTTC-3′ containing *Xho*I or *Eco*RI sites and was subcloned into pEGFP-N1 (Clontech), which possesses a CAG enhancer and CMV promoter. pDsRed-Atxn1-86Q was constructed by subcloning the full-length Atxn1 cDNA fragment, amplified from pCI-Atxn1-82Q (Okazawa *et al*, 2002) with primers 5′-TCATCTCGAGCTATGAAATCCAACCAAGAGCGGAG-3′ and 5′-TCATGAATTCCTACTTGCCTACATTAGACCGGC-3′, between the *Xho*I and *Eco*RI sites of pDsRed-monomer-C1 (Clontech) downstream of the CMV immediate-early enhancer/promoter. During subcloning, the CAG repeat number was changed from 82 to 86, but in the final plasmid, no other sequence was changed. The pAAV1-GFP and pAAV1-HMGB1-GFP vectors were constructed by inserting HMGB1 cDNA between the *Xba*I and *Eco*RV sites of the pAAV1 vector.

### AAV vector construction

The AAV vector plasmids contained an expression cassette, consisting of a human cytomegalovirus immediate-early promoter (CMV promoter) and the human growth hormone first intron, followed by cDNA encoding either rHMGB1, rHMGB1-GFP (fusion protein), or GFP; woodchuck hepatitis virus posttranscriptional regulatory element (WPRE); and a simian virus 40 polyadenylation signal sequence (SV40 poly[A]) between the inverted terminal repeats of the AAV3 genome. Transfection and purification methods were described previously (Li *et al*, 2006). Briefly, the vector, an AAV2 *rep* and AAV1 *vp* expression plasmid, and an adenoviral helper plasmid, pHelper (Agilent Technologies) were co-transfected into HEK293 cells by the calcium phosphate co-precipitation method. The recombinant viruses were purified by isolation from two sequential continuous CsCl gradients, and the viral titres were determined by qRT–PCR as follows: 40 cycles of 95°C/15 s, 60°C/30 s, 72°C/1 min 30 s, 75°C/15 s with WPRE forward primer (5′-ATTGCTTCCCGTATGGCTTTCA-3′) and WPRE reverse primer (5′-TCAGCAAACACAGTGCACACCA-3′) to amplify nt 1319–1201 of woodchuck hepatitis virus 2. We used AAV3 ITRs (Muramatsu *et al*, 1996) that are recognized by the AAV2 Rep protein and are available for encapsulation of other AAV serotypes. ITRs of AAV3 are compatible with Rep of AAV1, AAV2, AAV8 or AAV9 and can be used to produce pseudo-types of AAV1/3, AAV2/3, AAV8/3 or AAV9/3 (Iwata *et al*, 2013; Miyazaki *et al*, 2012; and our unpublished observations). Among various AAV vectors, AAV2/3 drives strong and relatively specific expression in Purkinje cells as shown by immunohistochemical analysis (Fig8F).

### Cerebellar surface injection of the virus vector

Virus vector injections into the cerebellar surface were performed on 5-week-old mice that were anesthetized with Nembutal (intraperitoneally) and mounted on a stereotaxic apparatus (Narishige). The forehead was tilt down at 20°. A hole of 1 mm diameter was made using ELA steel bar (Shofu, Japan) at −9.2 mm from bregma, ± 0 mm lateral to the midline. A glass syringe was inserted 3.5 mm from the outer surface of the bone hole along the internal surface of the occipital bone. Eight microlitres of an AAV1-GFP, AAV1-HMGB1 or AAV1-HMGB1-GFP virus solution (∼1 × 10^12^ particles) was injected in four orientations (60, 90, 270 and 330° clockwise rotation from the posterior to anterior line). At each orientation, 2 μl was injected by means of a micropump (Narishige) at the rate of 0.5 μl/min. This method reproducibly yields an efficient supply of virus vectors to the surface of the cerebellum. The fluorescent images of whole brains were obtained using a digital camera attached to a fluorescence stereoscopic microscope (Olympus, SZX10). We divided Atxn1-KI mice by a simple randomization method for injection of virus vector.

### The mitochondrial membrane potential

ShRNA knock-down experiments were performed with two types of HMGB1-shRNA-RFP (Cat. No: TF316576): HMGB1-shRNA1 (FI363513, AGTGCTCAGAGAGGTGGAAGACCATGTCT), HMGB1-shRNA2 (FI363516, CTTCAGTTGTCTCTGATGCAGCTTATACG) and an ineffective shRNA-RFP (TR30015) as a negative control purchased from OriGene. HeLa cells were transfected using Lipofectamine 2000 (Invitrogen) following the manufacturer's instructions, and the cells were cultured for another 2 days before analysis. Mitochondria were visualized by staining with MitoTracker Deep Red (Molecular Probes) at final concentration of 200 nM for 40 min. Micrographs were taken under a FluoView FV10i-w confocal microscope (Olympus). For siRNA-mediated knock-down, HeLa cells were transfected with siRNA (NC, SR30004: universal scrambled negative control; HMGB1-A, SR302140A: AGCAUGGGAUUAUUAGAAUCAAACA; HMGB1-B, SR302140B: GGGAGGCAAUUUAGAUAAGUGUAAA) using RNAiMAX (Invitrogen). 48 h after transfection, JC-1 (5,5′,6,6′-tetrachloro-1,1′,3,3′-tetraethylbenzimidazolylcarbocyanine iodide; Invitrogen), a more direct indicator of in the mitochondrial membrane potential (ΔΨm), was added to the culture medium (at 10 μg/ml). After incubation for 10 min and removal of JC-1, fluorescent images were acquired using a FluoView FV10i-w confocal microscope (Olympus) at 527 nm and 581 nm.

### Live imaging of mitochondria

Live cell imaging was performed on HeLa cells co-transfected with HMGB1-EGFP and DsRed or Atxn1-86Q-DsRed and cultured at 37°C and 5% CO_2_. Images were acquired every 2 s for 10 min with a FluoView FV10i-w confocal microscope (Olympus). DsRed at 558 nm and MitoTracker Deep Red at 644 nm were discriminated by a Cy5 filter (635 nm). The number of fission and fusion events during 10 min in 100-μm^2^ mitochondrial area was quantified. The expression of Atxn1 and HMGB1 was tested using DsRed and EGFP as shown in Fig3A.

### CAP resistance assay

The chloramphenicol (CAP) resistance assay was performed according to the method described previously (Aamann *et al*, 2010). In brief, pDsRed-monomer-C1 (control), pDsRed-Atxn1-86Q (Atxn1-86Q) or pDsRed-Atxn1-86Q + pCMV-HMGB1-EGFP (Atxn1-86Q-HMGB1) are transfected using the Lipofectamine 2000 reagent (Invitrogen, USA) into HeLa cells cultured at 300 cells/well in 6-well dishes in DMEM supplemented with 10% foetal bovine serum, 2 μg/ml puromycin and 1 mM sodium pyruvate. Chloramphenicol (200 μg/ml) was added to the medium 24 h after transfection. In parallel, similarly transfected cells were incubated without CAP. The cultures were maintained until colonies formed, then fixed with methanol and stained with 0.5% methylene blue. The ratio between the colony numbers in CAP+/CAP− conditions was calculated.

### Mitochondrial DNA damage assay

As shown in Fig4A, DNA was extracted from cerebellar tissues of Atxn1-KI mice (Atxn1-KI), double-transgenic mice (Atxn1-KI;HMGB1) and the background C57BL/6 mice. As shown in Fig5A, DNA damage was induced in L929 cells (ECACC, England, UK) by 8 Gy (130KPv, 13 min 42 s, X-ray Cabinet system, Faxitron Bioptics), and the cells were collected at 10 and 180 min after irradiation and used for DNA extraction. Transient transfection of the same plasmids as in CAP resistance assay was performed with Lipofectamine 2000 (Invitrogen, USA) 48 h before irradiation. As shown in Fig7A–G, DNA damage was induced in Atxn1-KI mice (Atxn1-KI), double-transgenic mice (Atxn1-KI;HMGB1) and the background C57BL/6 mice at 13 weeks of age by 20 Gy X-ray (130KPv, 26 min 38 s, X-ray Cabinet system), and the cerebellar tissues were sampled at 10, 180 and 300 min after irradiation. From the cerebellar tissues or the transfected cells, total DNA samples including nuclear and mitochondrial DNA were extracted. Briefly, tissues or cell pellets were minced in extraction buffer (10 mM Tris–HCl pH 8.0, 50 mM KCl, 1.5 mM MgCl_2_, 0.45% Tween-20, 0.45% NP-40) and centrifuged, and the pellet was resuspended in extraction buffer with 40 μg/ml proteinase K, incubated at 50°C for 8 h and centrifuged at 10,000 × *g*, and to enrich mitochondrial DNA, low-molecular-weight DNA (50 bp to 30 kb) in the supernatant was isolated using a PCR clean-up kit (Axygen, MA, USA).

The assay is based on the assumption that PCR amplification of longer cDNA becomes difficult when mitochondrial DNA contains more breaks (Jendrach *et al*, 2005). Total DNA was isolated from the mouse cerebellum. A long fragment of mtDNA (10.1 kb, *Mus musculus*, mitochondrion, 3278–13367) was amplified by semiquantitative PCR with the primers 5′-GCCAGCCTGACCCATAGCCATAATAT-3′ and 5′-GAGAGATTTTATGGGTGTAATGCGG-3′. A short fragment of mtDNA (241 bp, *Mus musculus*, mitochondrion, 14665–14906) was amplified with the primers 5′-CCTCCCATTCATTATCGCCGCCCTTGC-3′ and 5′-GTCTGGGTCTCCTAGTAGGTCTGGGAA-3′. Amplification was performed using LA-Taq (Takara). The conditions for long-fragment amplification from cerebellar tissues was 94°C for 30 s (denaturation), 60°C for 30 s (annealing) and 72°C for 1,200 s (extension; 21 or 22 cycles), and that for L929 cells was 94°C for 20 s, 68°C for 1,200 s and extension (23 cycles), respectively. The conditions for long-fragment amplification were as follows: 94°C for 20 s for denaturing, 55°C for 20 s for annealing and 72°C for 60 s for extension (28 cycles).

A long fragment of nuclear DNA (10.4 kb; hypoxanthine phosphoribosyltransferase, chromosome X, 53013838–53024221) was amplified with the primers 5′-CCACCAGGCGTCACCCTT-3′ and 5′-GCTAATGAATGCTTCAGAGAGGC-3′. A short fragment of nuclear DNA (180 bp; glyceraldehyde 3-phosphate dehydrogenase, chromosome 6, 125163189–125163369) was amplified with the primers 5′-AGCCCAGAACATCATCCCTG-3′ and 5′-GATGACATCAAGAAGGTGGTG-3′. PCR was performed with LA-Taq (Takara), and the conditions for the long or short nuclear DNA fragment were similar to those for mitochondrial DNA.

### 8-OHdG measurements

L929 cells were transfected with DsRed, Atxn1-86Q-DsRed or Atxn1-86Q-DsRed+HMGB1-EGFP for 48 h. DNA damage was induced in L929 cells by 8 Gy X-ray irradiation (130 KPv, 13 min 42 s, X-ray Cabinet system). Ten minutes after X-ray irradiation, cells were harvested and DNA was extracted using the DNA Extractor TIS kit (Wako, Japan). After preparation with 8-OHdG Assay Preparation Reagent Set (Wako, Japan), the amount of 8-OHdG was measured using a highly sensitive 8-OHdG ELISA kit (Japan Institute for the Control of Aging [JaICA], Japan).

### Mitochondrial enzyme histochemical analysis

For the succinate dehydrogenase (SDH) enzyme histochemical analysis, 10-μm fresh frozen sections were made and incubated for 120 min at 37°C in the medium for SDH activity (0.1 M Tris buffer 30 ml, pH 7.0, containing 300 mg sodium succinate, 0.75 mg phenazine methosulphate and 30 mg nitroblue tetrazolium). Following the reaction, the slices were rinsed in physiological saline, extracted with acetone, 60, 90 and 60% in that sequence, rinsed in distilled water and mounted on a glycerine jelly.

For the cytochrome oxidase (COX) histochemical analysis, 10-μm fresh frozen sections were cut and incubated for 120 min at 37°C in the medium for COX activity (0.1 M Tris-maleate buffer 27 ml, pH 7.0, mixed with 1% MnCl_2_ 3 ml, DAB (3,3′-diaminobenzidine tetrahydrochloride) 60 mg and 0.1% H_2_O_2_ 0.3 ml). Then, the slices were rinsed in distilled water and incubated in 1% CuSO_4_ for 5 min. After the rinse in distilled water, the slices were dehydrated and mounted on a synthetic resin.

### Electron microscopy

Mouse brains were fixed overnight at 4°C with a mixture of 2% paraformaldehyde and 2% glutaraldehyde in 0.1 M phosphate buffer. In the case of immunoelectron microscopy, 30-μm-thick sections were made from mouse brains fixed with a mixture of 2% paraformaldehyde and 0.1% glutaraldehyde in 0.1 M phosphate buffer. These sections were incubated sequentially with 1% BSA for 20 min, with an anti-HMGB1 antibody (AHM0915, dilution 1:100; ATGen, Seongnam-si, Korea) overnight at 4°C and with mouse anti-IgG antibodies conjugated with 1.4-nm gold particles (#2002, dilution 1:50; Nanoprobes, NY, USA) for 1 h at room temperature. The immunogold-labelled sections were fixed for 10 min in 2% glutaraldehyde and silver-enhanced with the fresh mixture of 0.2% w/v silver acetate in water and 0.5% w/v hydroquinone in citrate buffer (2.55% w/v citric acid and 2.35% w/v trisodium citrate in water) for 10 min at room temperature. Then, the slices were incubated with 0.05% sodium acetate for 1 min and 0.05% gold chloride for 2 min.

The samples were postfixed in 1% osmium tetroxide (OsO4), embedded in Epon, sliced into 1-μm-thick sections, stained with toluidine blue and viewed under a light microscope to select suitable areas for investigation. Ultrathin 80-nm-thick sections were made from the selected areas and stained with uranyl acetate and lead citrate. Electron micrographs were acquired using the Hitachi 7000 apparatus.

### Mitochondrial genome DNA sequencing

The mitochondrial fraction was prepared using the Mitochondria Isolation Kit for Tissue (Thermo Scientific, IL, USA) from cerebellar tissues of Atxn1-KI, Atxn1-KI;HMGB1 and wild-type (C57BL/6; *n* = 3). First, cerebellar tissues were homogenized using a Dounce homogenizer with five strokes on ice and centrifuged at 1,000 × *g* for 3 min at 4°C. The pellets were resuspended in BSA/Reagent A solution and incubated on ice for 2 min. Then, Mitochondria Isolation Reagent B was added and mixed, and the mixture was incubated on ice for 5 min. Next, Mitochondria Isolation Reagent C was added to the solution and the mixture was centrifuged at 700 × *g* for 10 min at 4°C. The supernatant was centrifuged at 12,000 × *g* for 15 min at 4°C. The resultant mitochondrial pellet was washed with wash buffer and centrifuged at 12,000 × *g* for 5 min at 4°C. The mitochondrial pellet was dissolved in extraction buffer (10 mM Tris–HCl pH 8.0, 50 mM KCl, 1.5 mM MgCl_2_, 0.45% Tween-20, 0.45% NP-40, 40 μg/ml proteinase K), from which mitochondrial DNA was isolated by phenol–chloroform extraction and ethanol precipitation. Library preparation was performed using the Ion Xpress Plus Fragment Library kit (Life Technologies, CA, USA) according to the protocol (Part 4471989 Rev. N) with minor modifications. Briefly, 100-ng samples of mitochondrial DNAs were sonicated by M220 Focused-ultrasonicator (Covaris, MA, USA) using 200-bp protocol and end-repaired. Following purification with AMPure beads (Beckman Coulter, MA, USA), Ion Torrent adapters P1 and one of the three types of Barcode (X) adapters were conjugated to purified mitochondrial DNA with DNA ligase. Nick repair was not performed in order to keep the damage in mitochondrial DNA.

Sequences of adapters were as follows: Ion P1 Adapter: 5′-CCACTACGCCTCCGCTTTCCTCTCTATGGGCAGTCGGTGAT-3′ and 5′-ATCACCGACTGCCCATAGAGAGGAAAGCGGAGGCGTAGTGGT*T*-3′ (asterisks indicate a phosphorothioate bond), Barcode (1) adapter: 5′-CTAAGGTAACGATCCACTACGCCTCCGCTTTCCTCTCTATGGGCAGTCGGTGAT-3′ and 5′-ATCACCGACTGCCCATAGAGAGGAAAGCGGAGGCGTAGTGGATCGTTACCTTAGT*T*-3′, Barcode (2) adapter: 5′-TAAGGAGAACGATCCACTACGCCTCCGCTTTCCTCTCTATGGGCAGTCGGTGAT-3′ and 5′-CTAAGGTAACGATCCACTACGCCTCCGCTTTCCTCTCTATGGGCAGTCGGTGATT*T*-3′, Barcode (3) adapter: 5′-AAGAGGATTCGATCCACTACGCCTCCGCTTTCCTCTCTATGGGCAGTCGGTGAT-3′ and 5′-ATCACCGACTGCCCATAGAGAGGAAAGCGGAGGCGTAGTGGATCGAATCCTCTTT*T*-3′.

Longer DNA fragments, which are not available for the sequencing reaction, were excluded with E-gel SizeSelect 2% Agarose Gel (Life Technologies), and DNA fragments from 100 to 350 bp were recovered. The fragments were amplified by PCR with the above primers (60 cycles). After purification with AMPure beads, concentration and size of the DNA library were determined using the Agilent BioAnalyzer 2100 with the High-Sensitivity DNA kit (Agilent Technologies, Waldbronn, Germany). Template-positive Ion Sphere Particles (ISPs) were prepared using Ion PGM Template OT2 200 kit (Cat. 4480974) and the Ion OneTouch2 system (composed of the Ion OneTouch2 instrument and Ion OneTouch ES instrument). Template-positive ISPs were sequenced by Ion Torrent PGM system using the Ion 318 Chip and Ion PGM Sequencing 200 kit v2 (Cat. #4482006). Base calling and alignment to the reference genome were performed using Torrent Suite 3.6 (Life Technologies).

The deduced sequences were analysed with reference to the sequence databases (http://www.ncbi.nlm.nih.gov/assembly/GCF_000001635.20/ and http://hgdownload.cse.ucsc.edu/goldenPath/mm10/chromosomes/), and only the reads from mitochondrial genome were selected. Contaminated reads containing nuclear genome were excluded.

### Laser-capture microdissection and gene expression profiling

To obtain purified Purkinje cells, cryosections (20 μm) were made from fresh cerebellar tissues of Atxn1-KI, Atxn1-KI;HMGB1 and WT littermate mice at 9 weeks of age and mounted on PEN-membrane glass slides (Leica). The slides were stained with toluidine blue, washed with PBS and quickly air-dried. Individual cell bodies of Purkinje cells were dissected from the cerebellar cortex using a laser-capture microdissection (LCM) instrument (Leica, LMD6500/7000). Purkinje cell areas of ∼3 mm^2^ were collected and dissolved with dissecting buffer. The RNeasy Micro kit (Qiagen) was used to extract total RNA from the LCM-isolated Purkinje cells. A Bioanalyzer (Agilent) was used to evaluate the quality and quantity of each RNA sample. The Ovation Pico RNA WTA system (NuGEN) was used to amplify cDNA for microarray (Agilent SurePrint G3 Mouse GE 8 × 60 K array).

The microarray analyses were repeated three times. All the array-spot signal intensity levels were normalized to the mean value of WT mice. Then, the values were compared using Student's *t*-test between WT mice and Atxn1-KI or between Atxn1-KI and Atxn1-KI;HMGB1 mice. The comparison was used to address the specific question whether a gene was changed in Atxn1-KI mice compared to the WT or the separate question whether the gene was changed in Atxn1-KI;HMGB1 mice compared to Atxn1-KI mice. The graph was integrated to save the space. In parallel, we performed one-way analysis of variance (ANOVA) followed by *post hoc* Tukey's HSD (honestly significant difference) test for comparison of the 3 genotypes without a specific interest in the combination. The microarray data from this study were submitted to the ArrayExpress database (http://www.ebi.ac.uk/arrayexpress/) and were assigned the identifier E-MTAB-2987.

### PANTHER analysis

Genes whose mRNA expression levels were significantly changed (*P* < 0.05, Student's *t*-test, *n* = 3) in the comparison of Atxn1-KI versus WT, Atxn1-KI;HMGB1 versus Atxn1-KI or genes whose expression was normalized in Atxn1-KI;HMGB1 compared to Atxn1-KI were functionally classified using the PANTHER classification system (Mi *et al*, 2013a). Relative upregulation or downregulation of a functional category was tested statistically by comparison of its proportion among the changed genes and the proportion among all genes of WT mice using Fisher's exact test in the PANTHER classification system (Mi *et al*, 2013b). In Supplementary Fig S4C, functional categories that were significantly upregulated or downregulated (*P* < 0.01, Fisher's exact test) are labelled by red and blue asterisks in pie charts or by red and blue arrows in the list, respectively.

### Gene ontology (GO) annotation and enrichment analysis

Genes whose mRNA expression levels were significantly changed in the comparison of Atxn1-KI with WT or Atxn1-KI;HMGB1 with Atxn1-KI were used to analyse GO annotation enrichment. The Tukey–Kramer test was employed to adjust *P*-values in multiple comparisons among the three groups. The Database for Annotation, Visualization and Integrated Discovery (DAVID Bioinformatics Resources 6.7; http://david.abcc.ncifcrf.gov/; Huang *et al*, 2009a,b) was used to test the enrichment of functional categories annotated by GO terms. The difference between the proportion of relative upregulation or downregulation of a functional category among the changed genes and the proportion among all genes of WT mice were tested statistically using Fisher's exact test. Functional categories were assumed significantly enriched at *P* < 0.05.

### Two-photon microscopy

Two-photon imaging of dendritic spines was performed using a laser-scanning microscope system, FV1000MPE2 (Olympus, Japan), equipped with an upright microscope (BX61WI, Olympus, Japan), a water-immersion objective lens (XLPlanN25xW; numerical aperture, 1.05) and a pulsed laser (Mai Tai HP DeepSee, Spectra Physics, USA). EGFP was excited at 890 nm and scanned at 500–550 nm. The scanning area used for three-dimensional imaging was 100 × 100 μm (1 μm Z steps, 1,024 × 1,024 pixels). Two weeks before imaging, AAV1-EGFP with the CMV enhancer/chicken β-actin promoter (titre 1 × 10^10^ vector genomes per millilitre, 1 μl) was injected into the cerebellar surface of mice under anaesthesia with 2.5% isoflurane. The brain was fixed with 4% paraformaldehyde and sliced into 100-μm-thick sections on a vibratome (Thermo Scientific, HM650V). Images obtained from the fourth and fifth cerebellar lobules were analysed for dendrite length, area and branching point number, or spine density, length, maximum diameter and protrusion subtypes using IMARIS 7.2.2 (Bitplane, Switzerland).

### Quantitative PCR analysis of Hmgb1, Cox-2 and Il-1β mRNA

Total RNA was isolated from cerebellar tissues of Atxn1-KI mice (Atxn1-KI), double-transgenic mice (Atxn1-KI;HMGB1-Tg), AAV1-HMGB1-GFP virus-injected Atxn1-KI mice (Atxn1-KI;HMGB1-AAV) and their background C57BL/6J mice at 9 weeks using the RNeasy mini kit (Qiagen, the Netherlands). Reverse transcription was performed using the SuperScript VILO cDNA Synthesis kit (Invitrogen, USA). *Hmgb1* cDNA (NM_010439, 174–367) was amplified with the primers 5′-TAAGAAGCCGAGAGGCAAAA-3′ (forward) and 5′-GCTGACAAGGCTCGTTATGA-3′ (reverse). For *Gapdh* (NM_008084, 651–830): 5′-AGCCCAGAACATCATCCCTG-3′ (forward) and 5′-GATGACATCAAGAAGGTGGTG-3′ (reverse), for *Cox2* (NM_011198, 1509–1702): 5′-AGAAGGAAATGGCTGCAGAA-3′ (forward) and 5′- GCTCGGCTTCCAGTATTGAG-3′ (reverse) and for *Il1b* (NM_008361, 298–497): 5′-CTGTGTCTTTCCCGTGGACC-3′ (forward) and 5′-CAGCTCATATGGGTCCGACA-3′ (reverse) were used. Amplification was performed using the THUNDERBIRD SYBR qPCR Mix (TOYOBO, Japan). The PCR conditions for amplification were 94°C for 60 s for initial denaturation, 95°C for 15 s for cyclic denaturation and 60°C for 30 s for extension (40 cycles). The expression level was normalized to *Gapdh* and calculated as a relative expression level.

### FACS analyses

HeLa cells treated with HMGB1-siRNA #1 (SR302140A; AGCAUGGGAUUAUUAGAAUCAAACA, Origene, USA), HMGB1-siRNA #2 (SR302140B; GGGAGGCAAUUUAGAUAAGUGUAAA, Origene, USA) or control siRNA (SR30004, Origene, USA) were used in FACS analysis. After 48 h of siRNA transfection, the cells were harvested by trypsinisation, and the resuspended cells were incubated with TMRM (10 nM; Molecular Probes, USA) for 30 min. For each analysis, 20,000 gated cells were processed by the FACSCalibur system, and TMRM fluorescence was analysed with CELLQuest (Becton Dickinson).

### Laser micro-irradiation and time-lapse imaging

Live cell imaging was performed on HeLa cells co-transfected with HMGB1-EGFP and DsRed, Atxn1-33Q-DsRed or Atxn1-86Q-DsRed. After culture at 37°C and 5% CO_2_ for 24 h, the cells were incubated with MitoTracker Deep Red (400 nM; Molecular Probes, USA) and Hoechst 33258 (4 μg/ml, Dojindo, Japan) for 30 min. Using the software (AIM4.2; Carl Zeiss, Germany) for microscopy (LSM510META, Carl Zeiss, Germany), MitoTracker-labelled mitochondria were irradiated with a UV laser (maximum power: 30 mW, laser output: 75%, wave length: 405 nm, iteration: 5, pixel time: 12 μs, objective: ×100, zoom: 2), and time-lapse images were acquired every 1 min.

### Quantitative analysis of mitochondrial DNA repair *in vitro*

DNA damage was induced in HeLa cells by 8 Gy of X-rays (130 KPv, 13 min 42 s, X-ray Cabinet system), and the mitochondrial fraction was prepared using the Mitochondria Isolation kit (Thermo Scientific, IL, USA). Mitochondrial membrane was disrupted by freeze-thaw cycles, and mitochondrial content was incubated with radioisotope-labelled nucleotides to quantify incorporation of nucleotides into damaged mitochondrial DNA during DNA damage repair. To test involvement of endogenous HMGB1 in mitochondrial DNA damage repair, we pretreated the mitochondrial fraction with an anti-HMGB1 antibody (ab18256, 1:20; Abcam, Cambridge, UK) for 2 h at 4°C, before DNA repair was induced by the addition of [α-^32^P]dCTP, [α-^32^P]dATP, [α-^32^P]dGTP and [α-^32^P]TTP (PerkinElmer) in reaction buffer from the Nick Translation kit (Roche) for 1 h at 37°C. We started the incubation with or without exogenous HMGB1 recombinant protein (0.0167 mg/ml; Sino Biological Inc.) also to evaluate participation HMGB1 in mitochondrial DNA damage repair. The amount of radioisotope-labelled DNA purified by phenol–chloroform extraction and ethanol precipitation was measured using a liquid scintillation counter (ALOKA LSC-5100).

### ChIP detection of HMGB1 binding to mitochondrial DNA

The mitochondrial fraction was prepared from HeLa cells transfected with pDsRed, pDsRed-Atxn1-33Q or pDsRed-Atxn1-86Q, using the Mitochondria Isolation kit (Thermo Fisher Scientific, IL, USA). The mitochondrial fraction was lysed in TNE buffer (10 mM Tris–HCl pH 7.5, 150 mM NaCl, 1 mM EDTA and 1% NP-40) and precipitated with an anti-HMGB1 antibody (0.02 mg/ml, Abcam). A mitochondrial DNA fragment (1,849 bp) bound to HMGB1 was amplified by PCR with the mitochondrial DNA-specific primers 5′-TAGCCATGCACTACTCACCAGA-3′ and 5′-GGATGAGGCAGGAATCAAAGAC-3′. The expected size of the amplified DNA fragment was 1,849 bp. The conditions for this amplification were 98°C for 10 s for denaturation, 55°C for 60 s for annealing and 72°C for 120 s for extension (40 cycles).

### Southern blot analysis of mitochondrial DNA

Two micrograms of total DNA samples including nuclear and mitochondrial DNA was extracted from cerebellar tissues of Atxn1-KI, Atxn1-KI;HMGB1-Tg and WT (C57BL/6) mice. Briefly, tissues were minced in extraction buffer (10 mM Tris–HCl pH 8.0, 50 mM KCl, 1.5 mM MgCl_2_, 0.45% Tween-20, 0.45% NP-40) and centrifuged, and the pellet was resuspended in extraction buffer with 40 μg/ml proteinase K, incubated at 50°C for 2 h, centrifuged at 10,000 × *g*, and to enrich mitochondrial DNA, low-molecular-weight DNA (50 bp to 30 kb) in the supernatant was isolated using a PCR clean-up kit (Axygen MA, USA). The DNA samples were digested with *Xho*I, separated by 0.7% agar gel electrophoresis and blotted using a nylon membrane (Hybond-N+, GE Healthcare Life Sciences) by the capillary transfer method. The DNA fragments were fixed to the membrane by UV irradiation (120 mJ/cm^2^). The mitochondrial DNA probe (10.5 kb) was generated by PCR from L929 cells as described in the section “Mitochondrial DNA damage assay” and digested by *Spe*I restriction enzyme before labelling with DIG-High Prime DNA Labelling kit II. The nylon membrane was hybridized with the mixture of labelled DNA fragments (3.3, 3.1, 2.0 and 1.7 kb), washed in 0.5× SSC containing 0.1% SDS at 65°C for 30 min two times and analysed using the DIG Detection kit II (Roche Diagnostics, Mannheim, Deutschland).

### Statistics

Survival curves were constructed using the Kaplan–Meier method, and survival rates were compared using log-rank (Mantel–Cox) test. Distributions of the read length of the mitochondrial genome from NGS were compared among the three mouse genotypes using Friedman's test followed by Wilcoxon rank-sum test. The mutation frequencies in the mitochondrial genome of the three mouse genotypes were compared using Fisher's exact test. Functional groups or categories from the PANTHER or GO analysis were evaluated using Fisher's exact test. For the above-mentioned experiments, a nonparametric distribution was assumed, while for the other experiments, the Gaussian distribution was hypothesized. In the latter case, unpaired Student's *t*-test was employed to compare two groups, and one-way ANOVA followed by the *post hoc* Tukey–Kramer test or Bonferroni correction was used for multiple testing of more than two groups. All the results were presented as mean ± SD. *P* < 0.01 were assumed to denote statistical significance in functional analysis. In the rest of statistical analyses, *P* < 0.05 assumed to denote statistical significance. For each experiment, sample size was estimated using power analysis based on preliminary experiments and/or prior studies. The number of samples or animals is specified in the respective figures, figure legends and/or supplementary figures. We provide exact *P*-values of all experiments in the *P*-value list as supplementary information (Supplementary Table S4).

### Ethics

This study was performed in strict compliance with the recommendations in the Guide for the Care and Use of Laboratory Animals of the National Institutes of Health (USA). This study was also approved by the Committees on Gene Recombination Experiments, Human Ethics and Animal Experiments of the Tokyo Medical and Dental University (document numbers 2010-215C3, 2011-22-3 and 0130225, respectively).

**The paper explained**

**Problem**

Spinocerebellar ataxia type 1 (SCA1) is an intractable neurodegenerative disease. The molecular changes caused by mutant Atxn1 have been one of the hot topics in the field, and a number of molecules have been suggested as mediators. However, there are no cases of successful clinical application of such candidate molecules when it comes to human patients.

We previously identified HMGB1 as a protein that is significantly decreased in the soluble nuclear fraction from primary cerebellar neurons expressing mutant Atxn1 according to mass spectrometry. However, detailed mechanism leading from the downregulation of HMGB1 to dysfunction of cerebellar neurons is not known. Moreover, the therapeutic effects have not been confirmed in mammalian models of the disease.

**Results**

In the present study, we tested the therapeutic effects of HMGB1 on mutant Atxn1-KI mice using transgenic and viral vector-mediated expression. The lifespan was remarkably prolonged by the add-back expression of HMGB1. Particularly, adeno-associated virus (AAV) vector-mediated delivery of HMGB1 into the cerebellum prolonged the lifespan of mutant Atxn1-KI mice from 217 to 365.5 days, and the maximum lifespan was increased from 274 to 448 days. Moreover, we found that HMGB1 might perform a function in DNA damage repair of the mitochondrial genome, in addition to the previously known functions of HMGB1 in nuclear gene transcription, nuclear DNA damage repair, cytosomal autophagy (mitophagy) and extracellular inflammation. In our transgenic and AAV-infected mice, no such side effects were observed (e.g. problems with inflammation or autophagy).

**Impact**

Our results may lead to the development of a novel treatment strategy against SCA1.

## For more information

http://omim.org/entry/164400

http://ghr.nlm.nih.gov/condition/spinocerebellar-ataxia-type-1

## Supporting Information

Source Data for Supplementary Figure S6

Review Process File
