# Supplementary figures and images for: HMGB1 facilitates repair of mitochondrial DNA damage and extends the lifespan of mutant ataxin-1 knock-in mice
Source: EMBO Mol Med. 2014 Dec 15;7(1):78–101. doi: 10.15252/emmm.201404392 (PMC4309669; doi:10.15252/emmm.201404392)

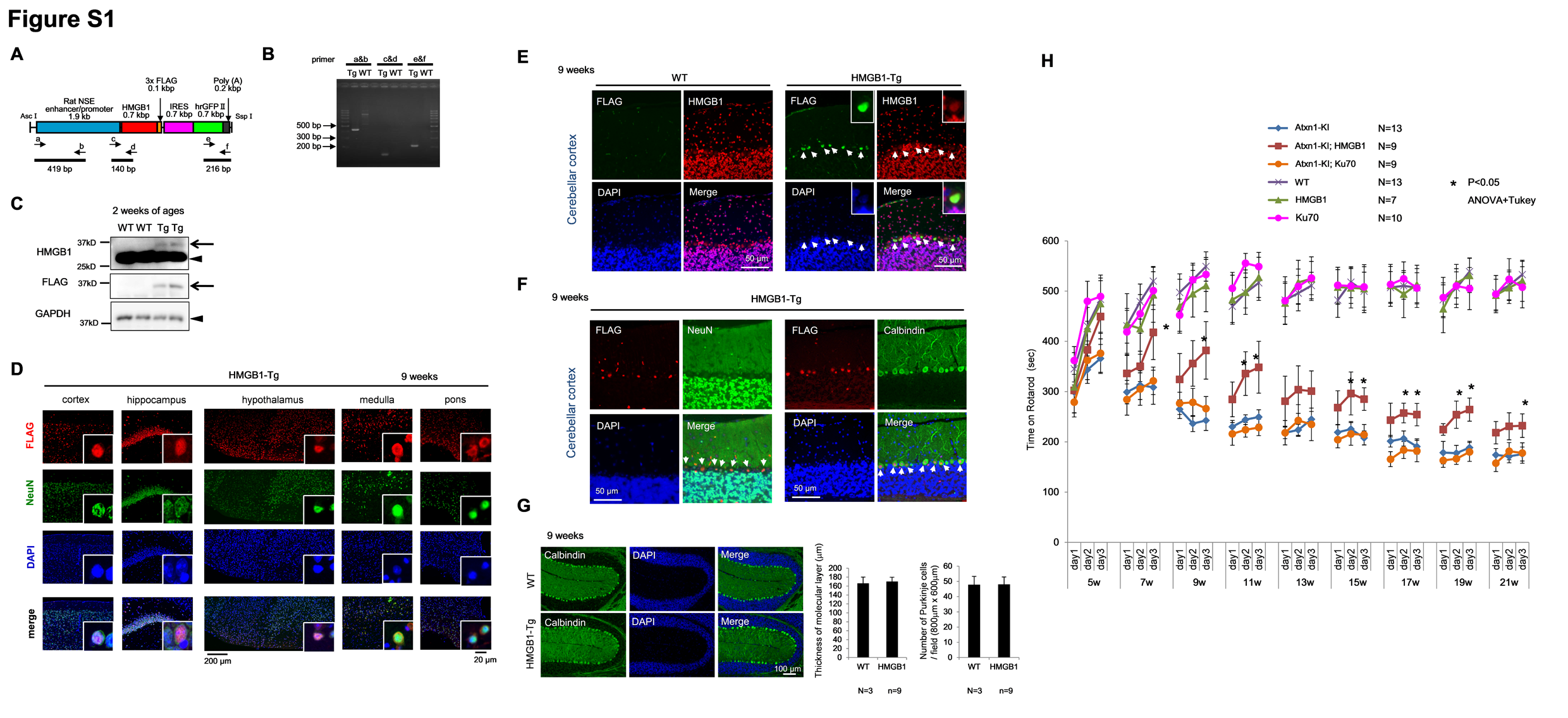

Supplement: Supplementary file 1 [file emmm0007-0078-sd1.tif]

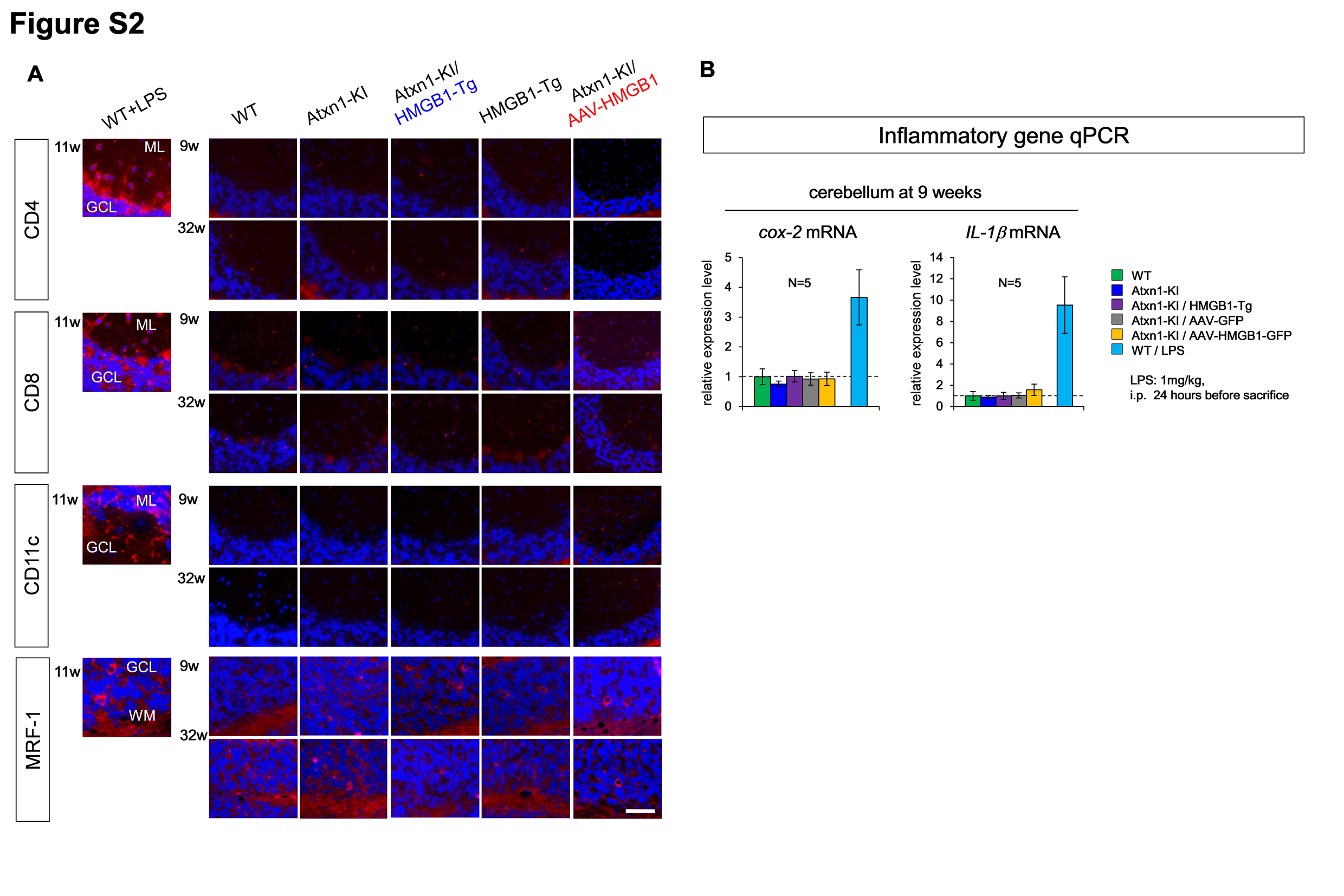

Supplement: Supplementary file 2 [file emmm0007-0078-sd2.tif]

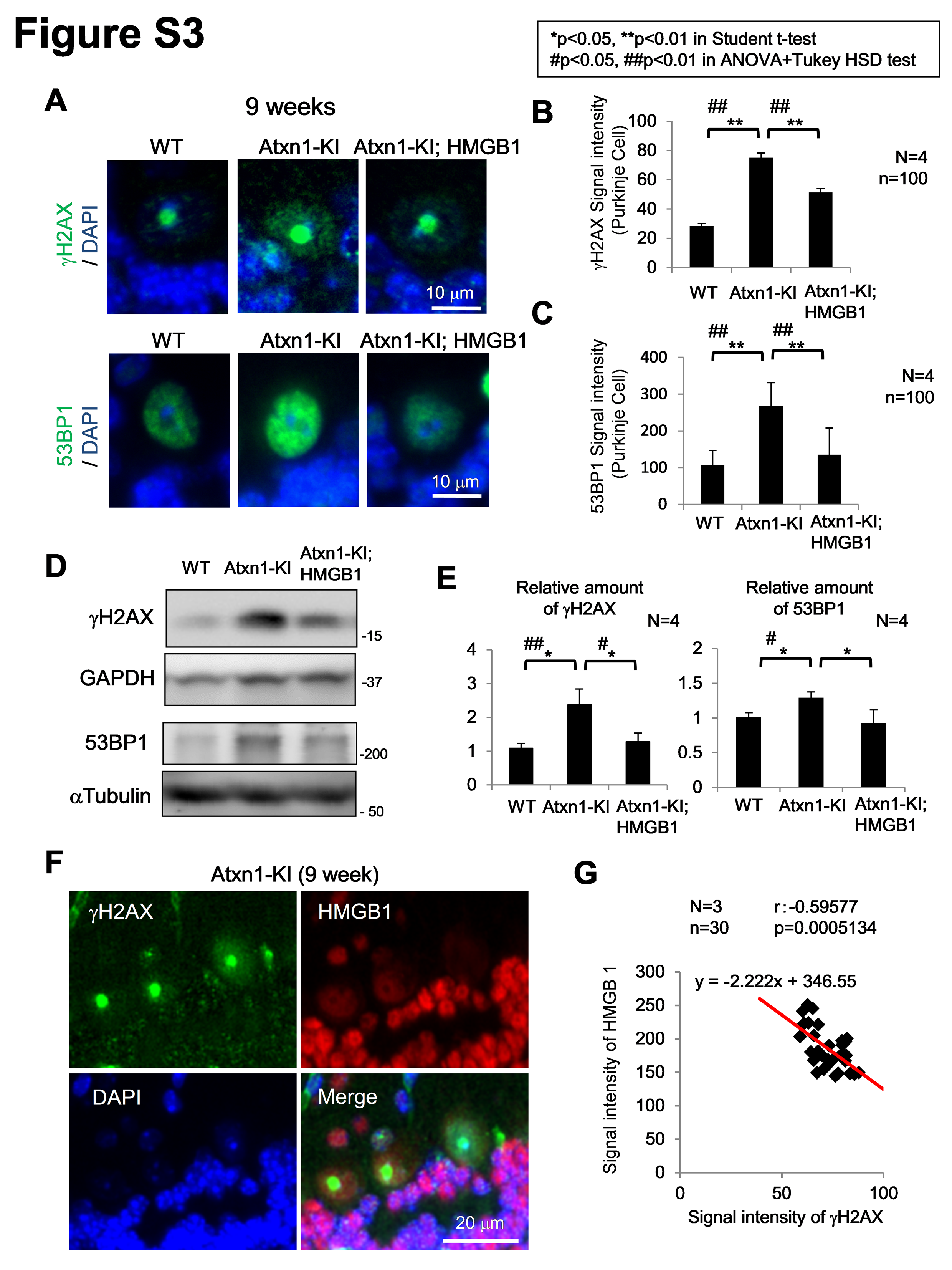

Supplement: Supplementary file 3 [file emmm0007-0078-sd3.tif]

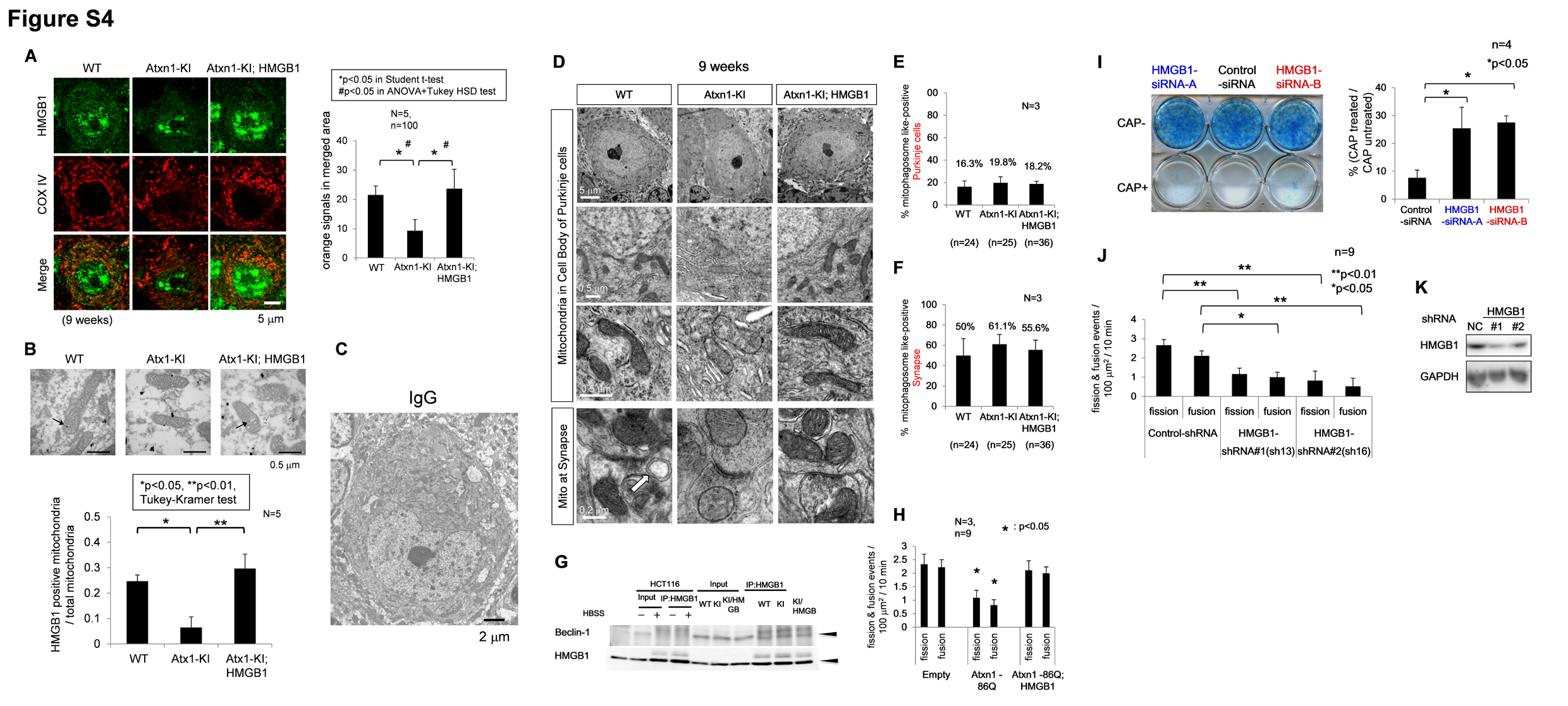

Supplement: Supplementary file 4 [file emmm0007-0078-sd4.tif]

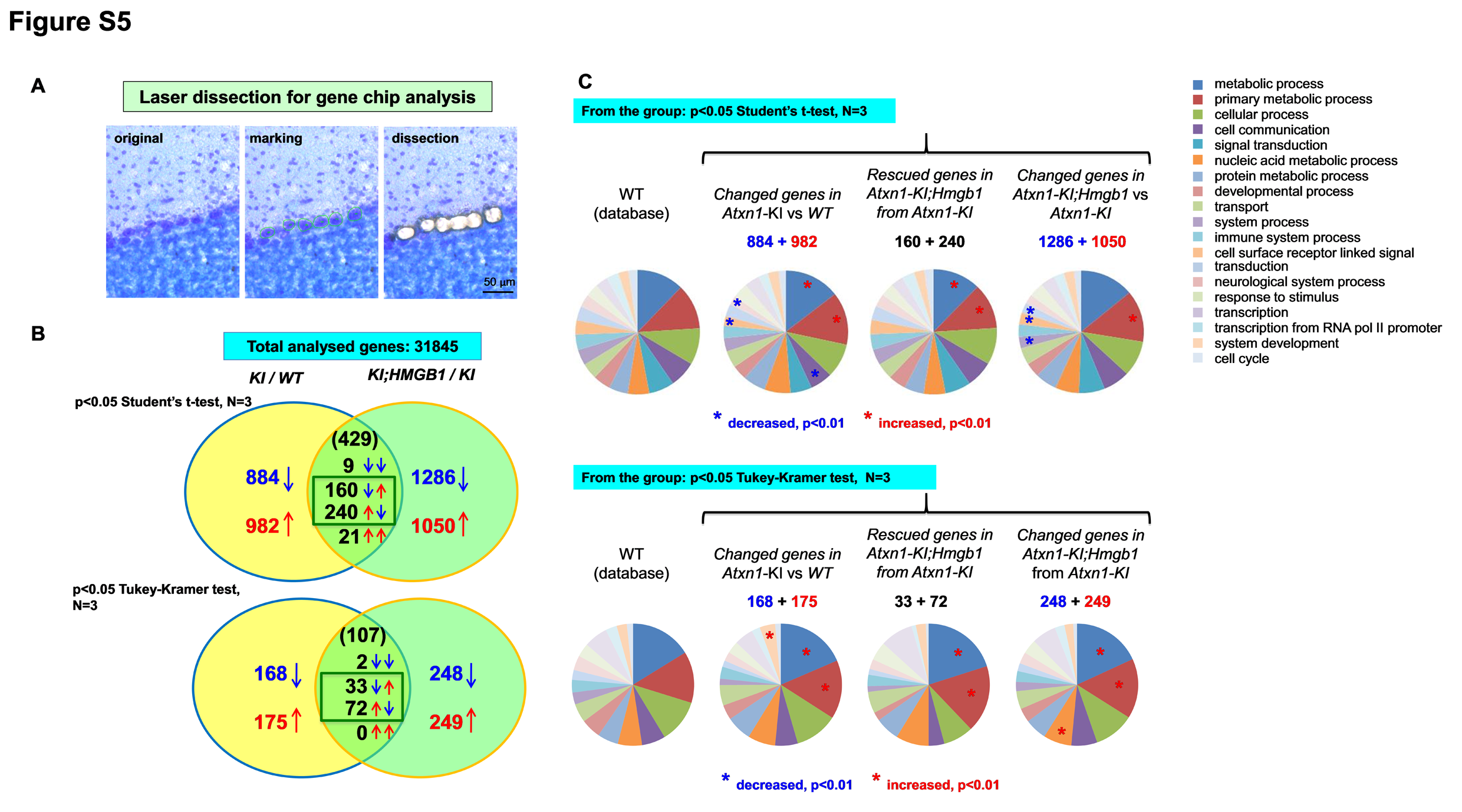

Supplement: Supplementary file 5 [file emmm0007-0078-sd5.tif]

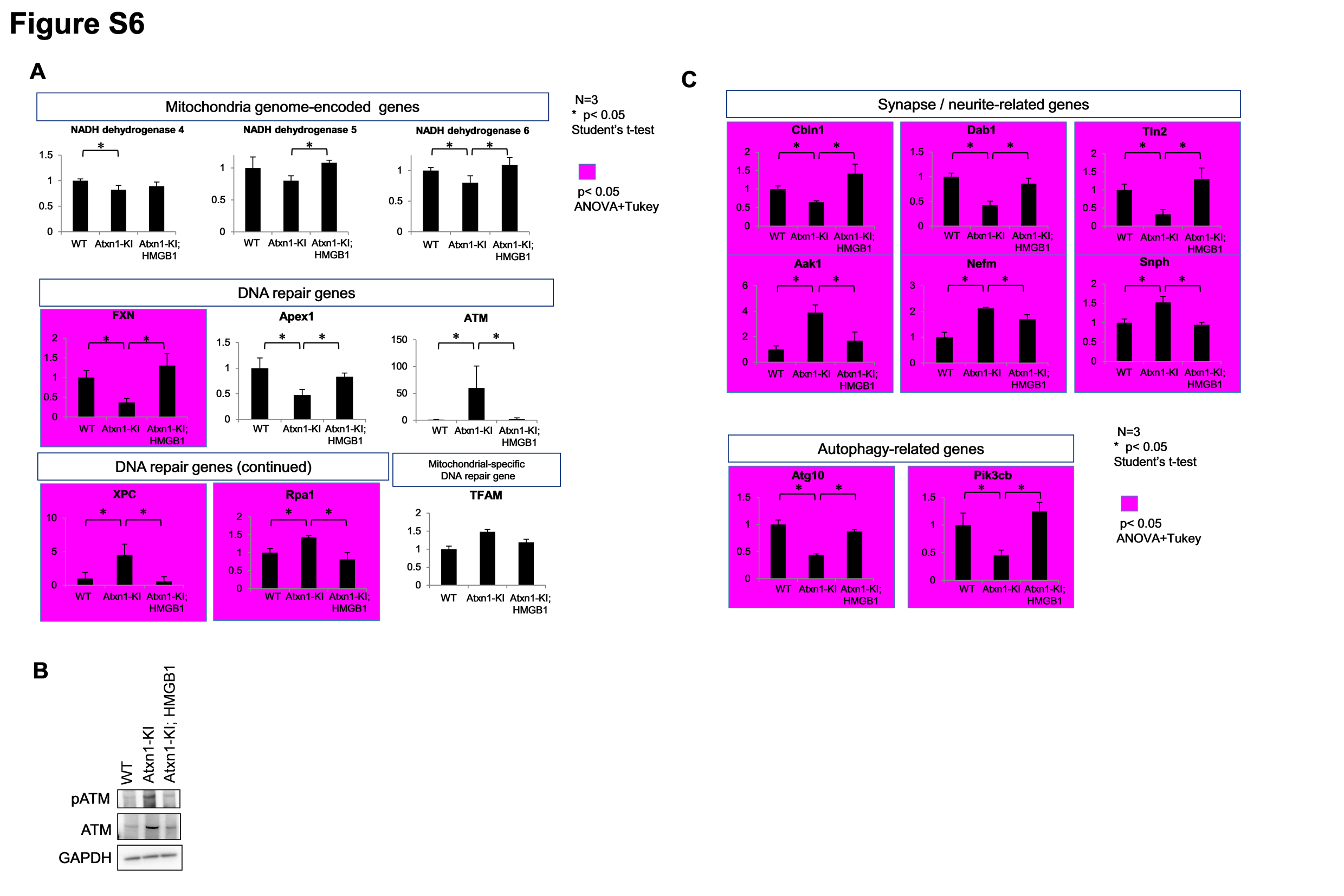

Supplement: Supplementary file 6 [file emmm0007-0078-sd6.tif]

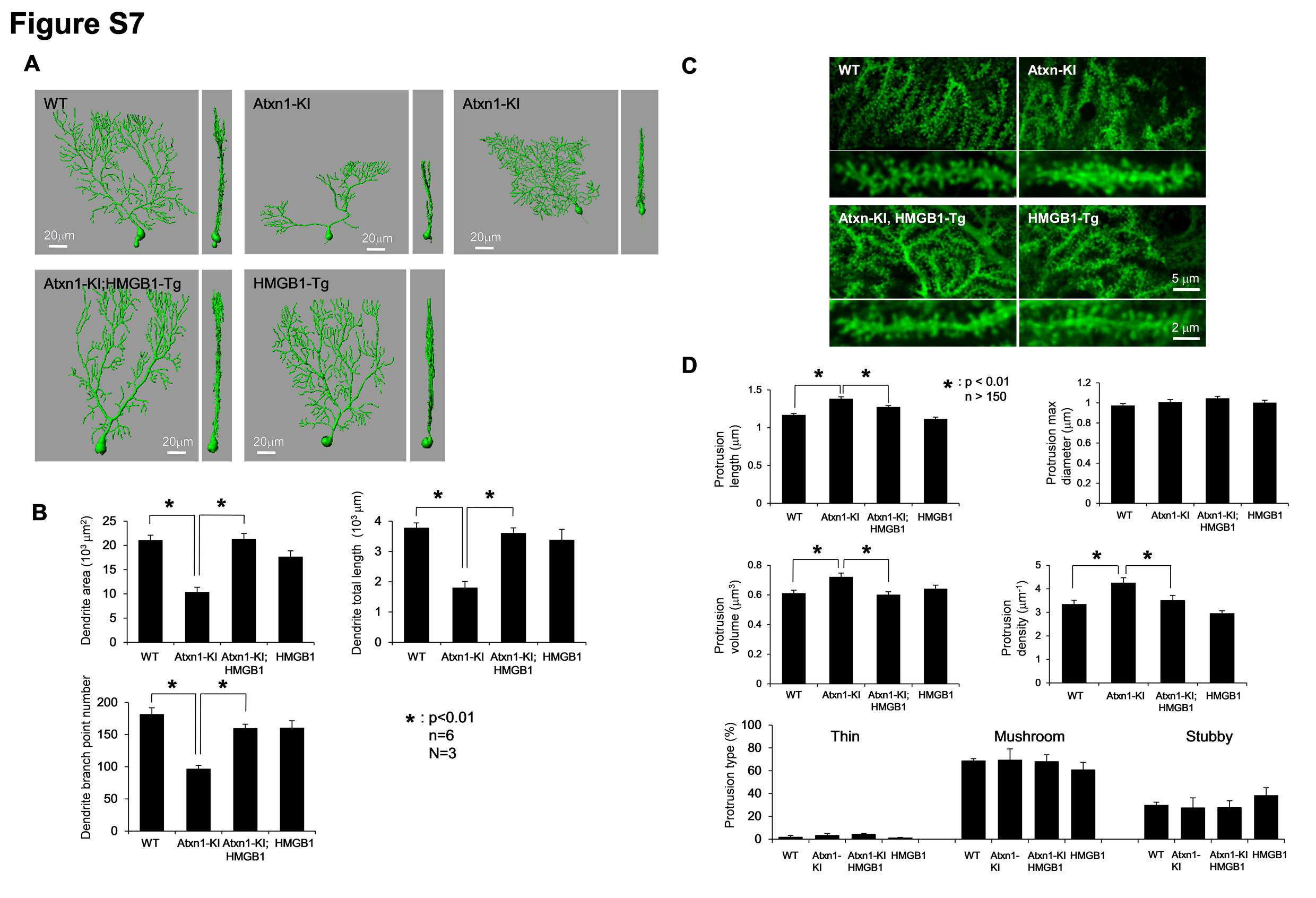

Supplement: Supplementary file 7 [file emmm0007-0078-sd7.tif]

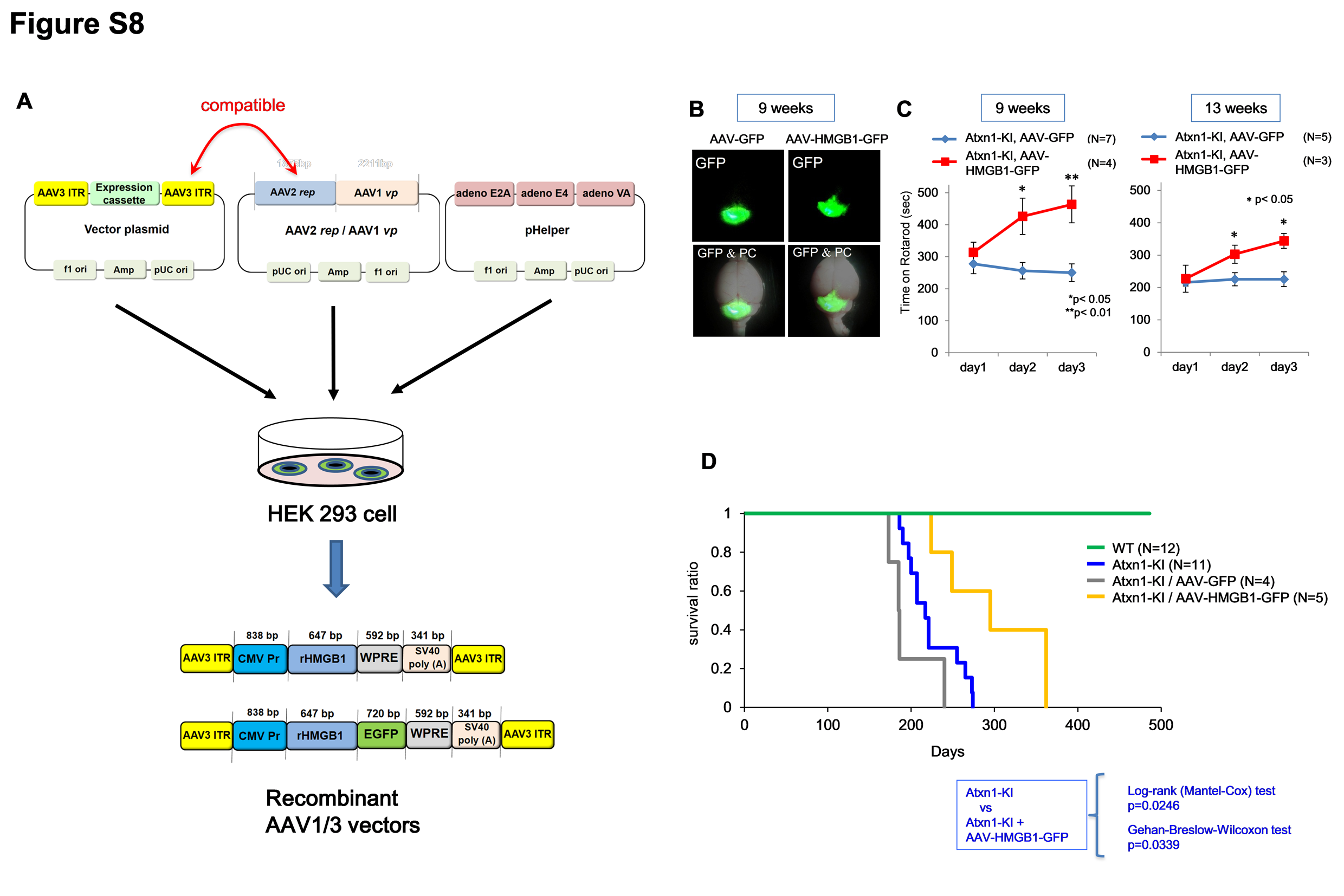

Supplement: Supplementary file 8 [file emmm0007-0078-sd8.tif]

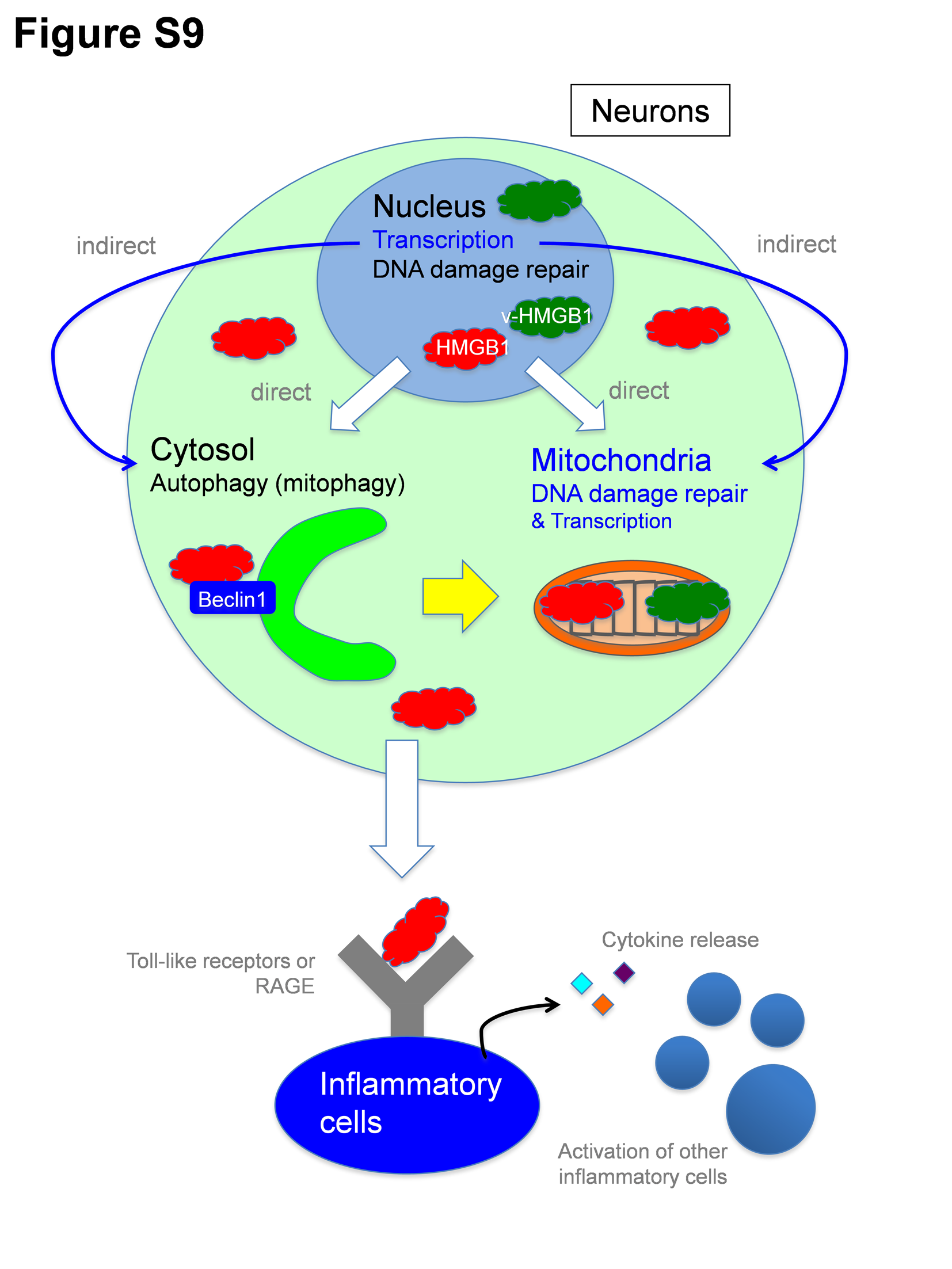

Supplement: Supplementary file 9 [file emmm0007-0078-sd9.tif]

Supplementary source data: original scans of gels

Sup.Fig.1C

HMGB1

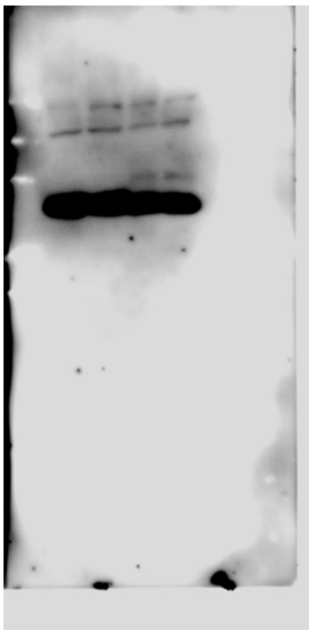

FLAG

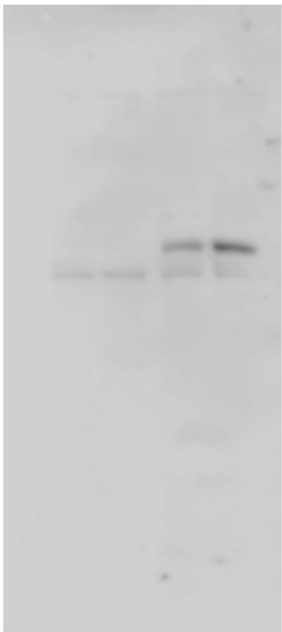

GAPDH

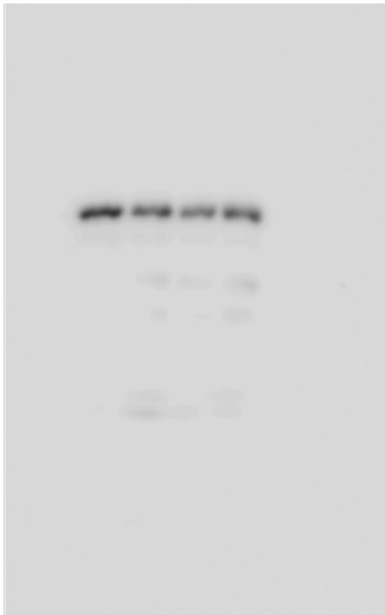

Supplement: Supplementary file 18 [file emmm0007-0078-sd18.pdf]

Supplementary source data: original scans of gels

Sup.Fig. 3D

$\gamma$ H2AX

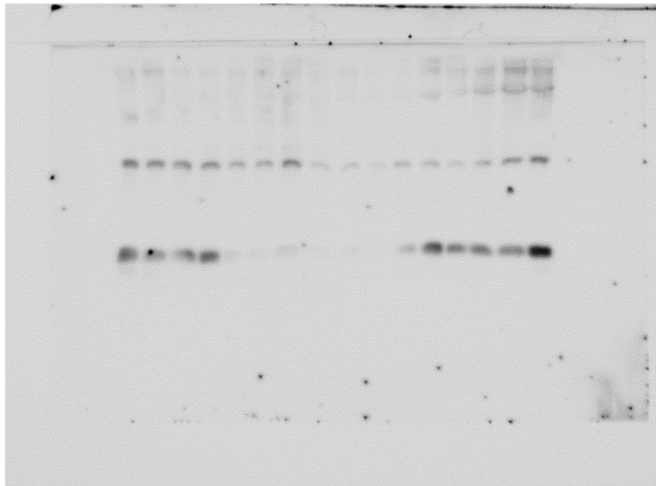

GAPDH

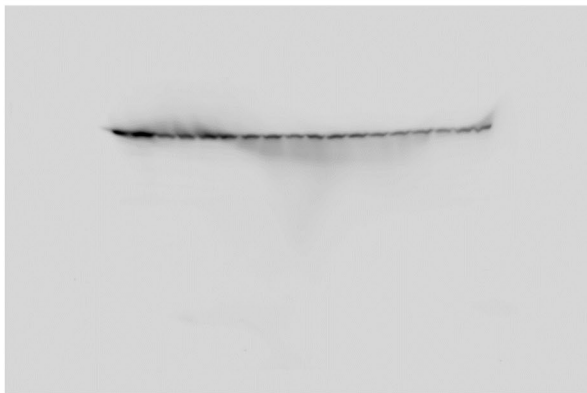

53BP1

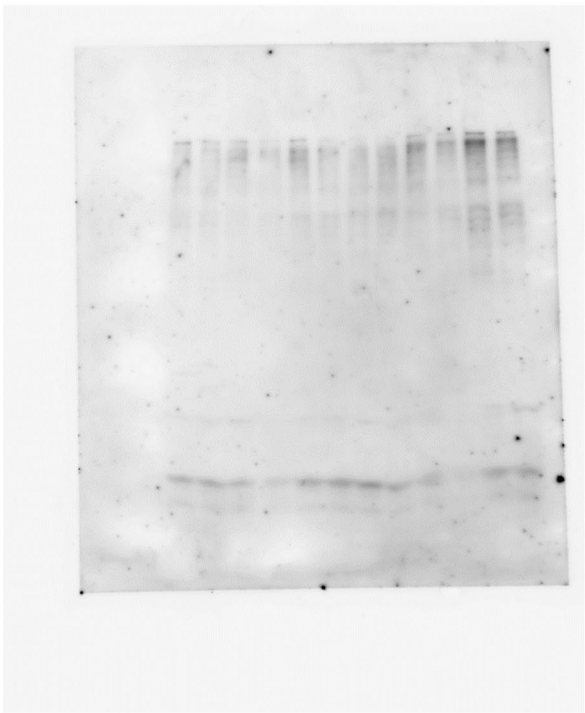

$\alpha$ -tubulin

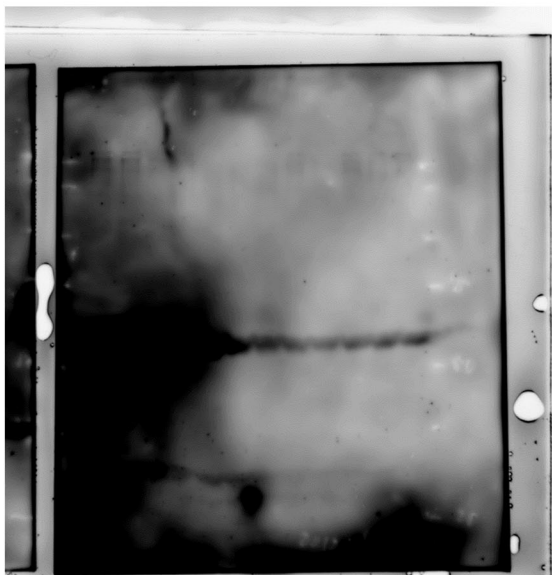

Supplement: Supplementary file 19 [file emmm0007-0078-sd19.pdf]

Supplementary source data: original scans of gels

Sup.Fig.4G

Beclin-1

HMGB1

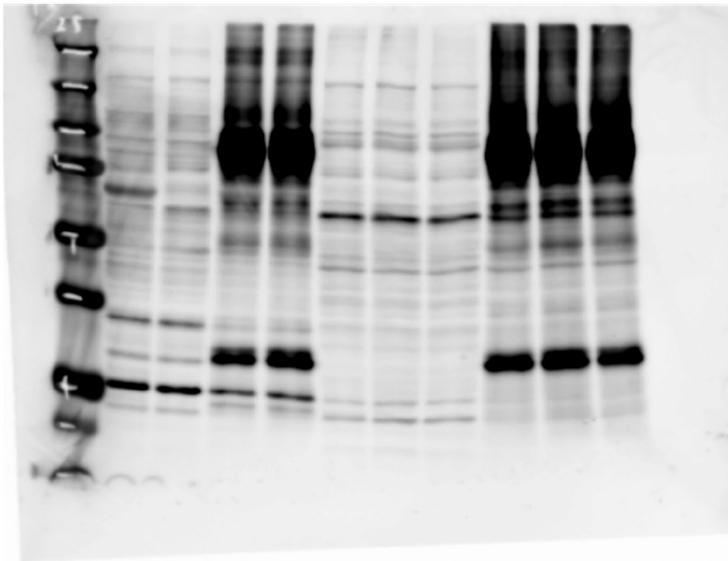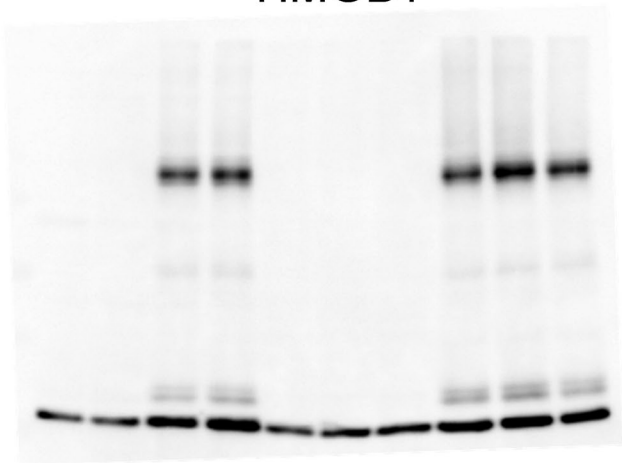

Sup.Fig.4K

HMGB1

GAPDH

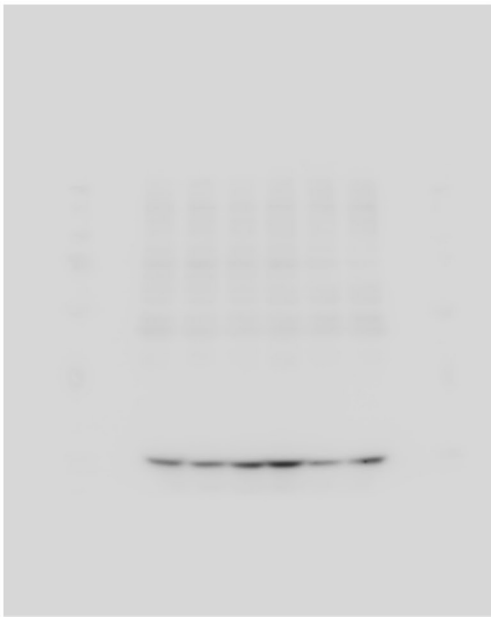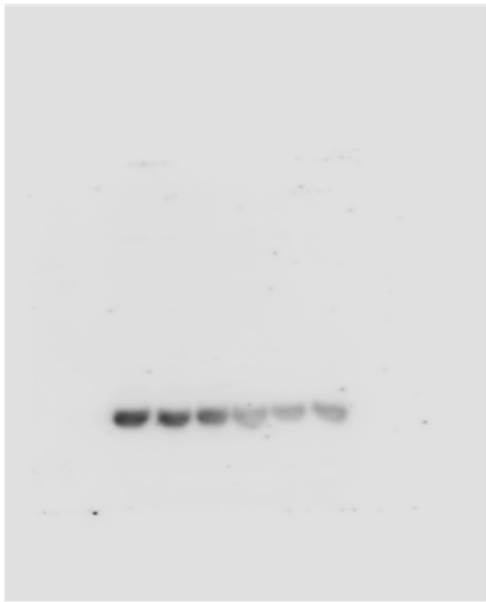

Supplement: Supplementary file 20 [file emmm0007-0078-sd20.pdf]

Supplementary source data: original scans of gels

Sup.Fig.6B

pATM

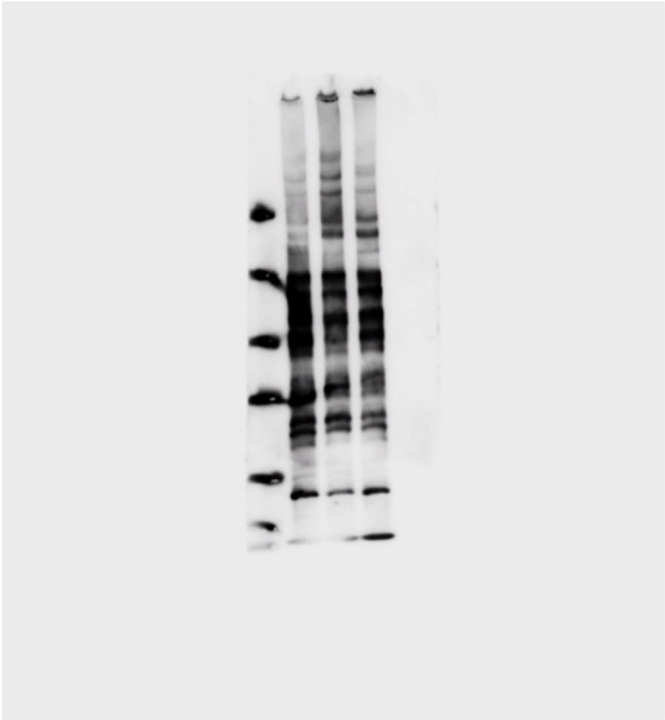

GAPDH

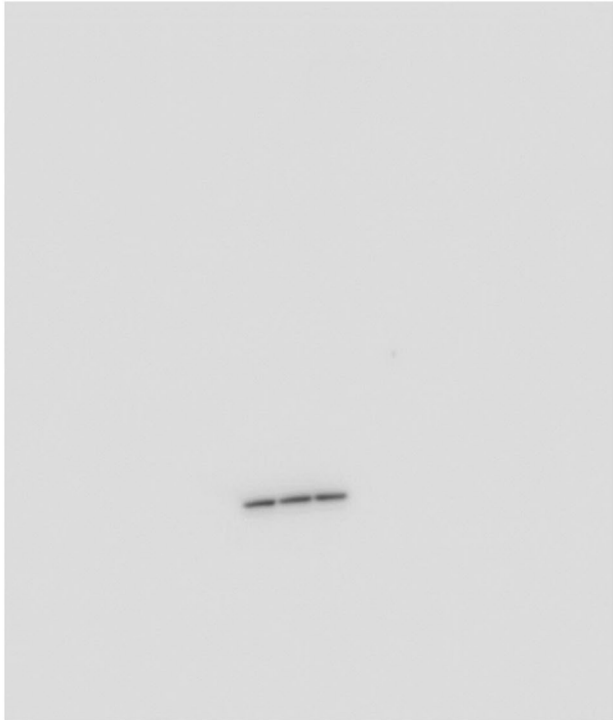

ATM

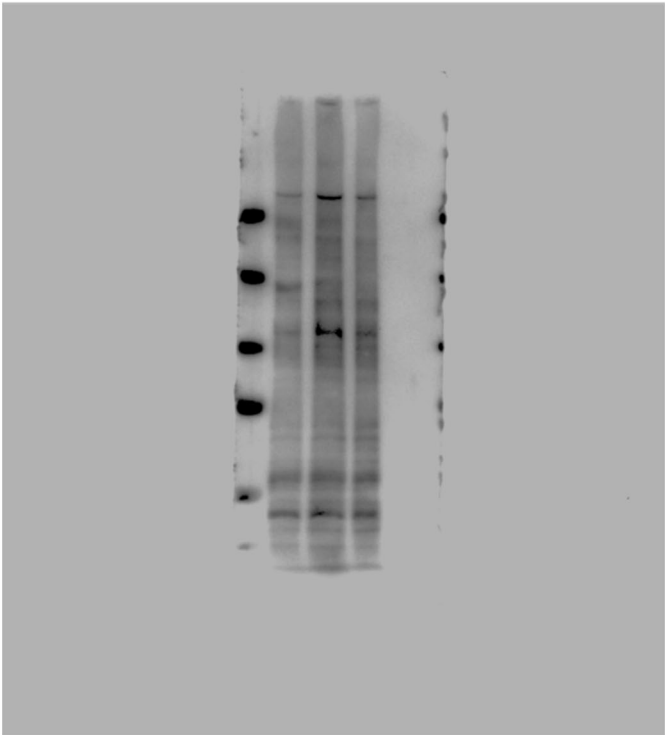

Supplement: Supplementary file 21 [file emmm0007-0078-sd21.pdf]

Fig. 1C

ubiquitin

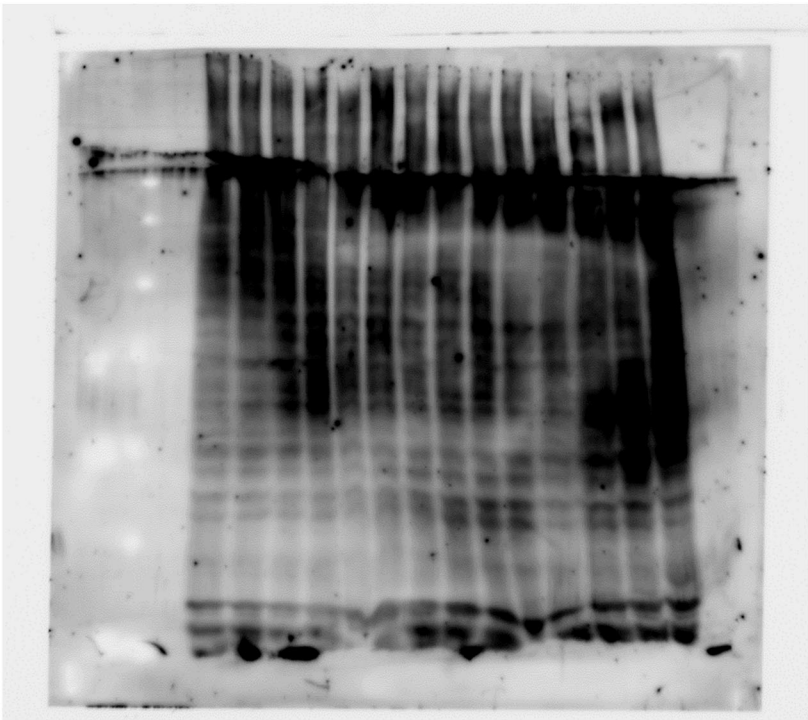

1C2

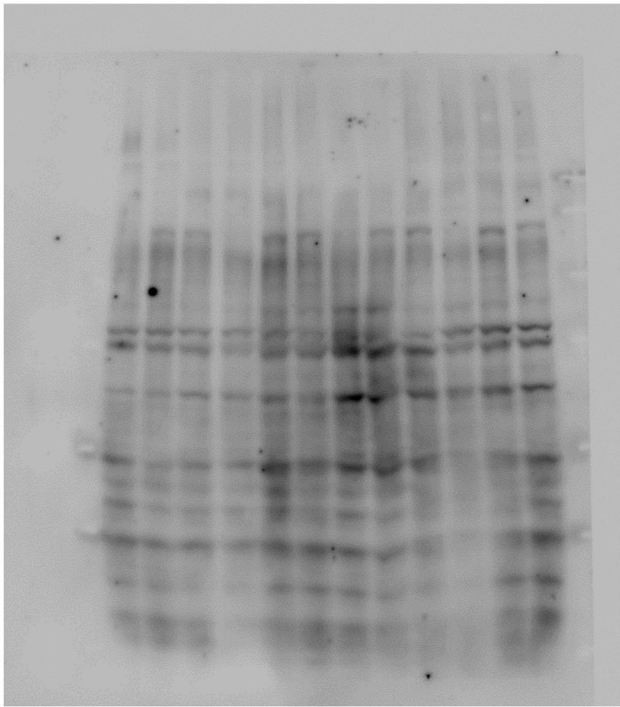

$\alpha$ Tubulin

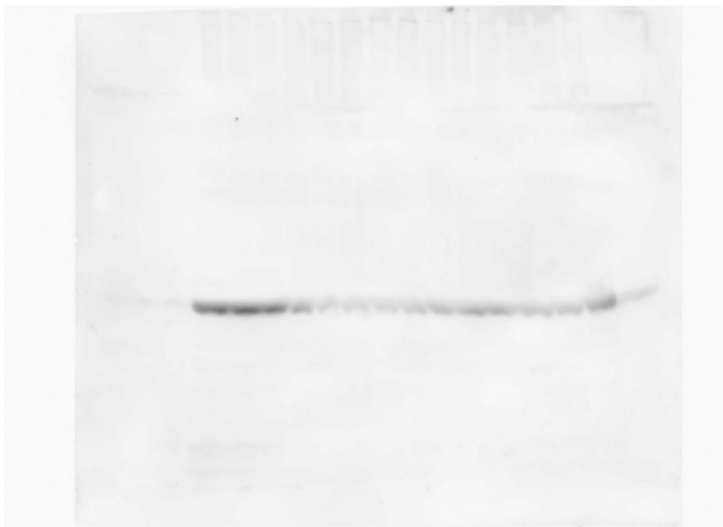

Supplement: Supplementary file 23 [file emmm0007-0078-sd23.pdf]

Fig. 3C

HMGB1

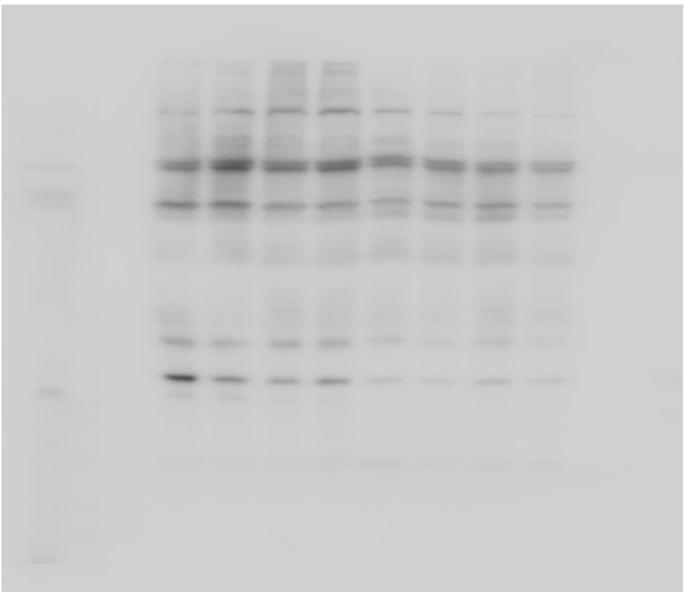

GAPDH

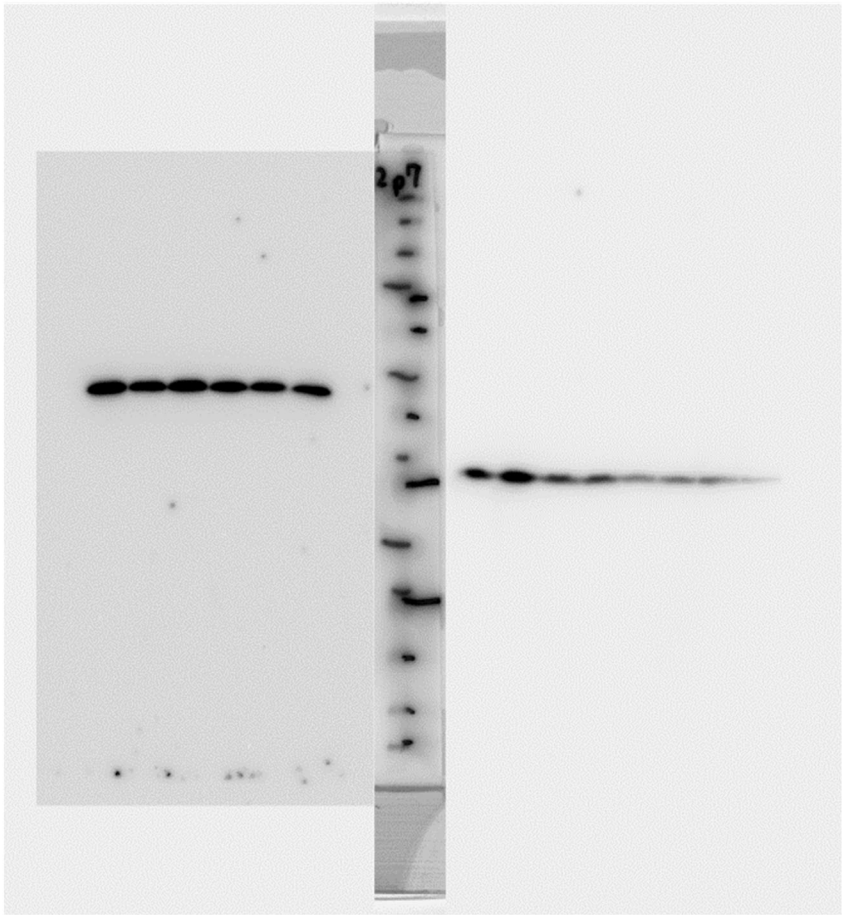

Supplement: Supplementary file 25 [file emmm0007-0078-sd25.pdf]

Fig. 4A

long fragment

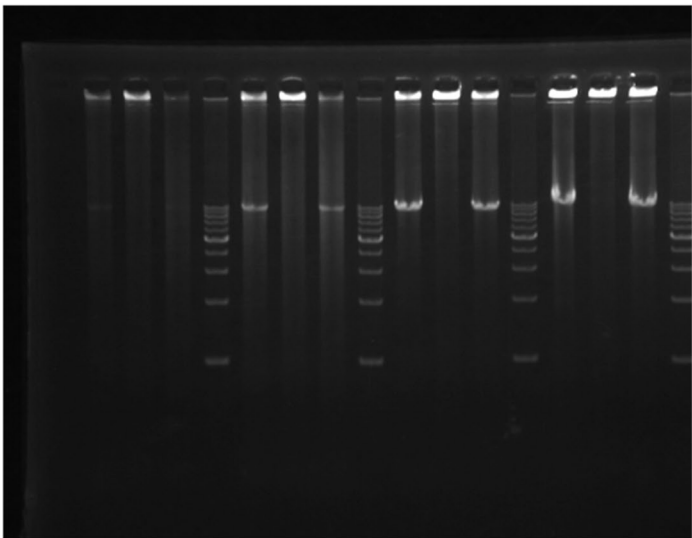

short fragment

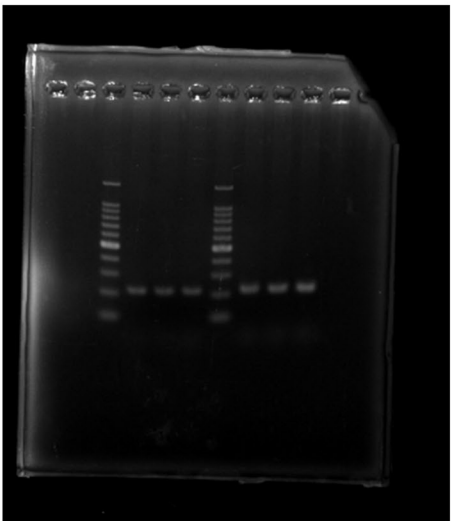

Fig. 4C

1C2

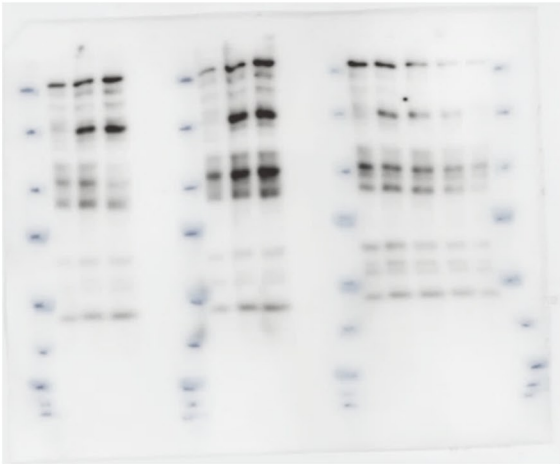

GAPDH

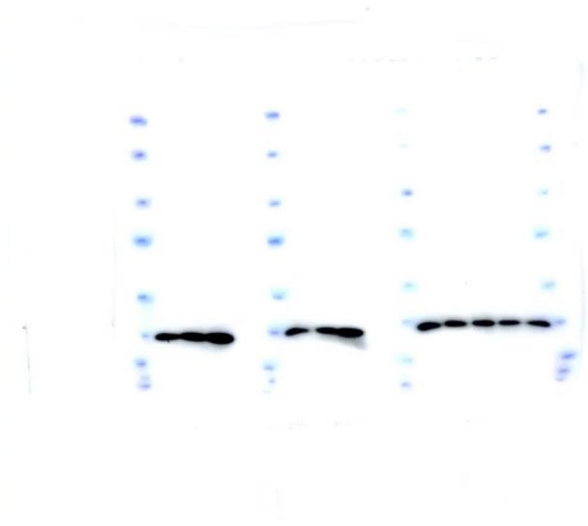

HMGB1

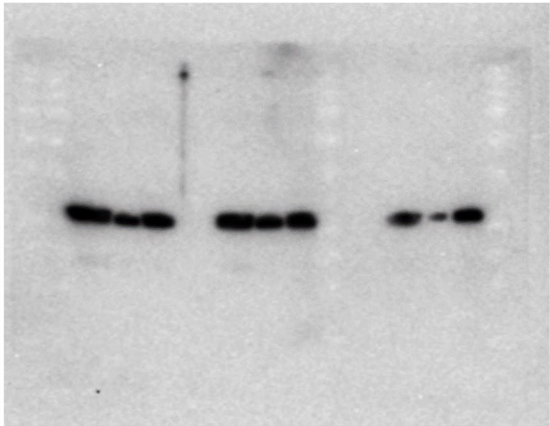

Supplement: Supplementary file 26 [file emmm0007-0078-sd26.pdf]

Fig. 5A

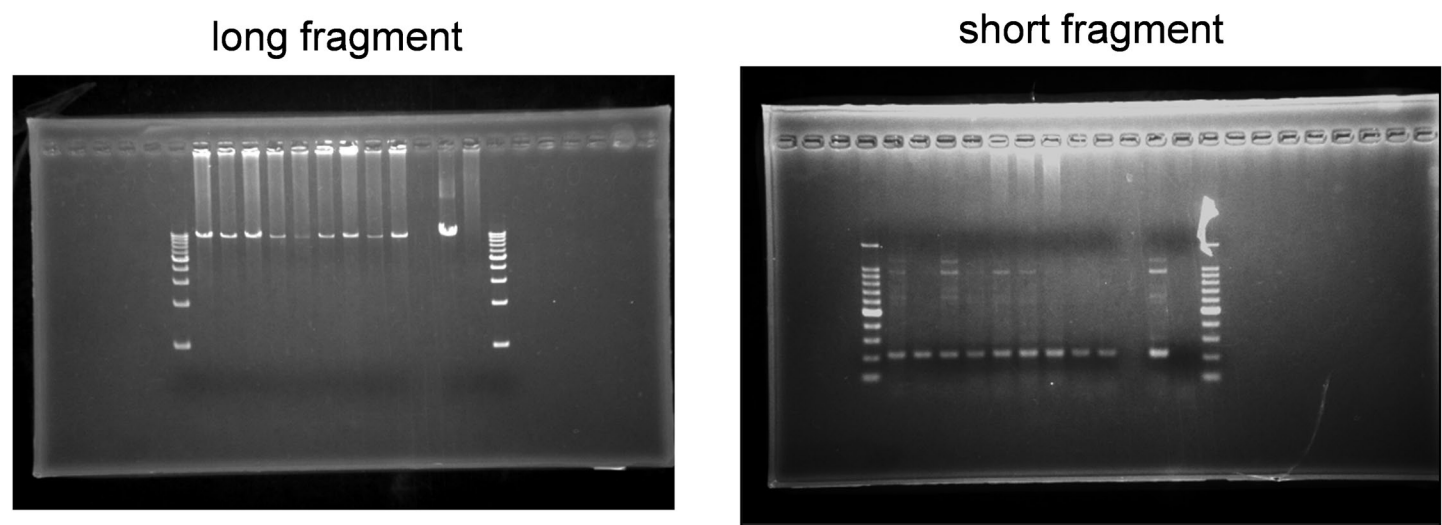

Fig. 5E

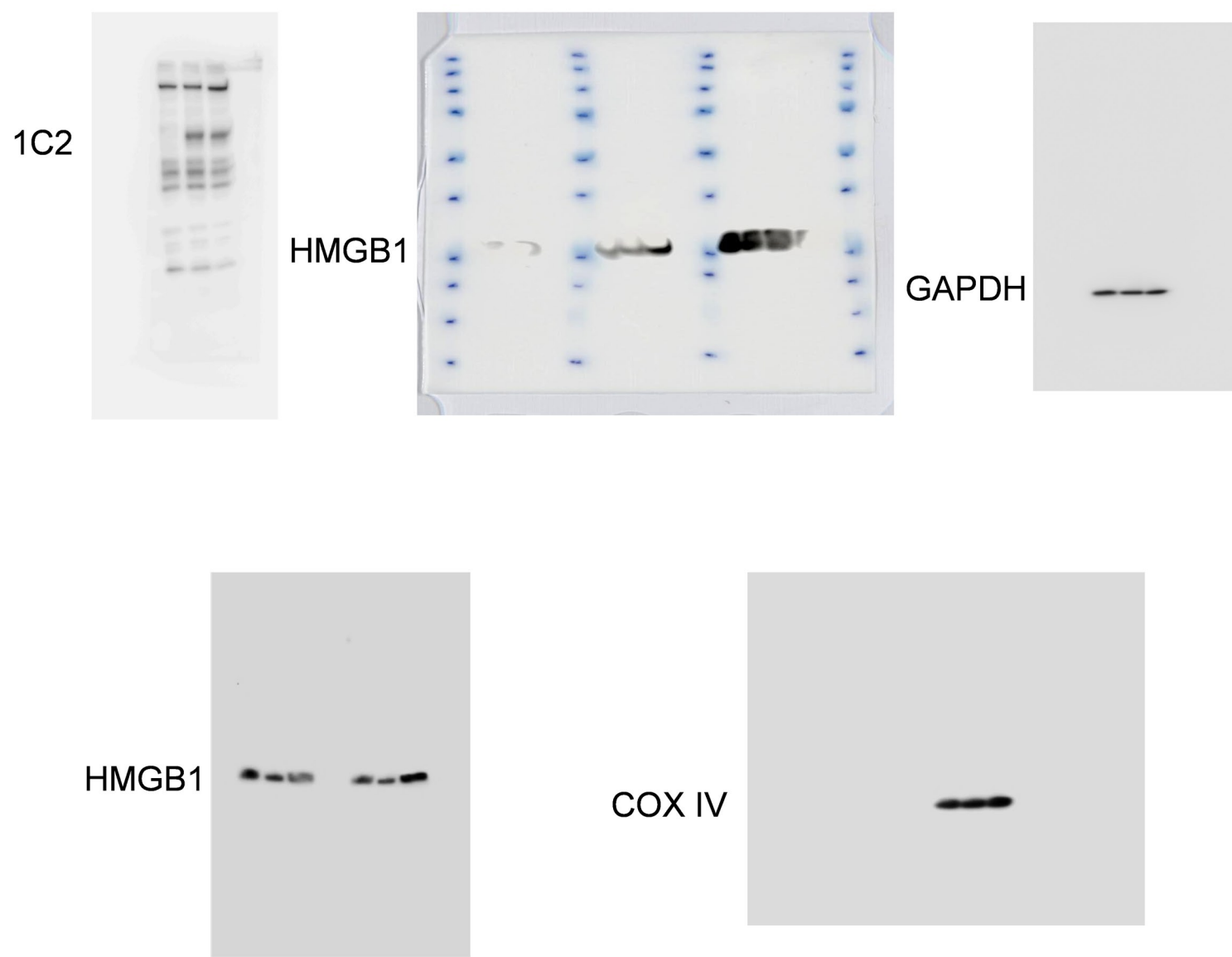

Fig. 5E

HP1 $\alpha$

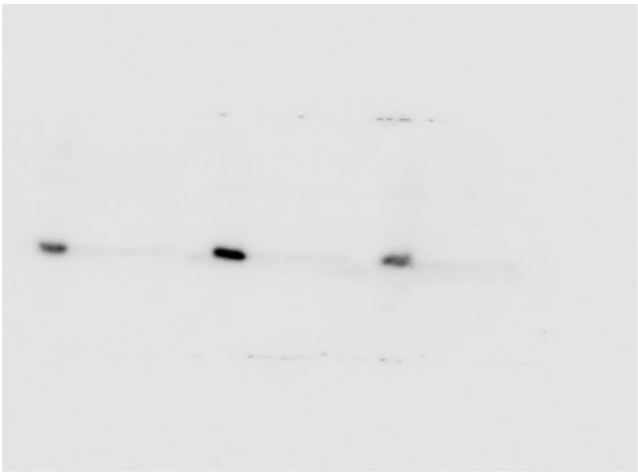

$\alpha$ -Tubulin

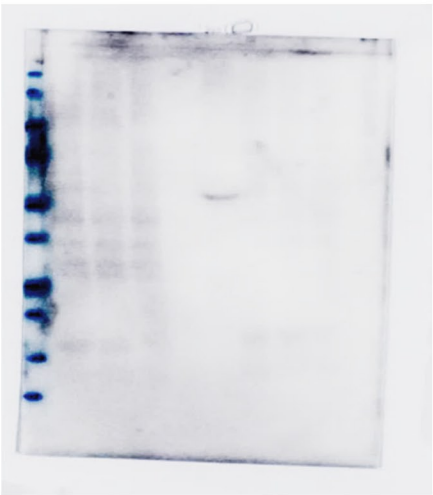

Supplement: Supplementary file 27 [file emmm0007-0078-sd27.pdf]

Fig. 6B

Atxn-1 (H-21)

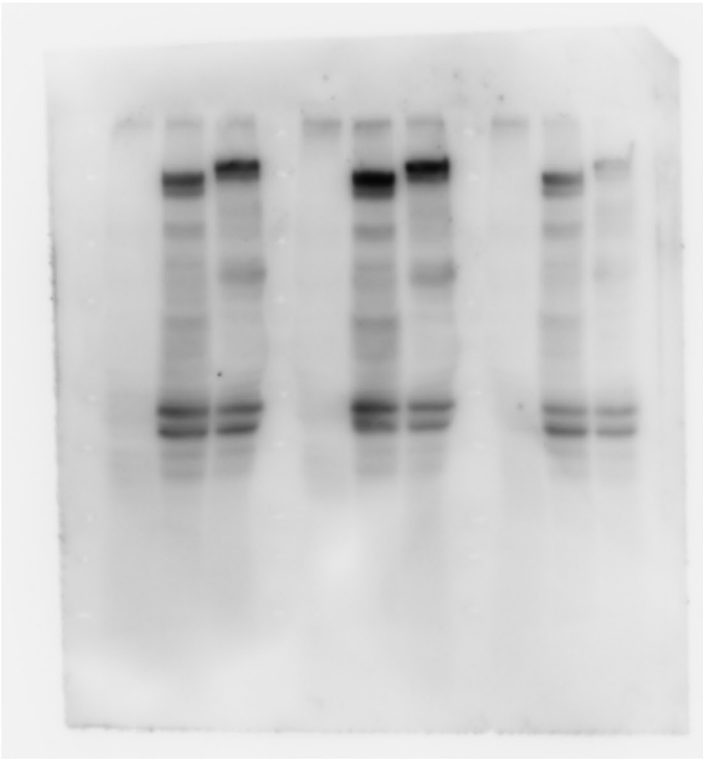

GAPDH

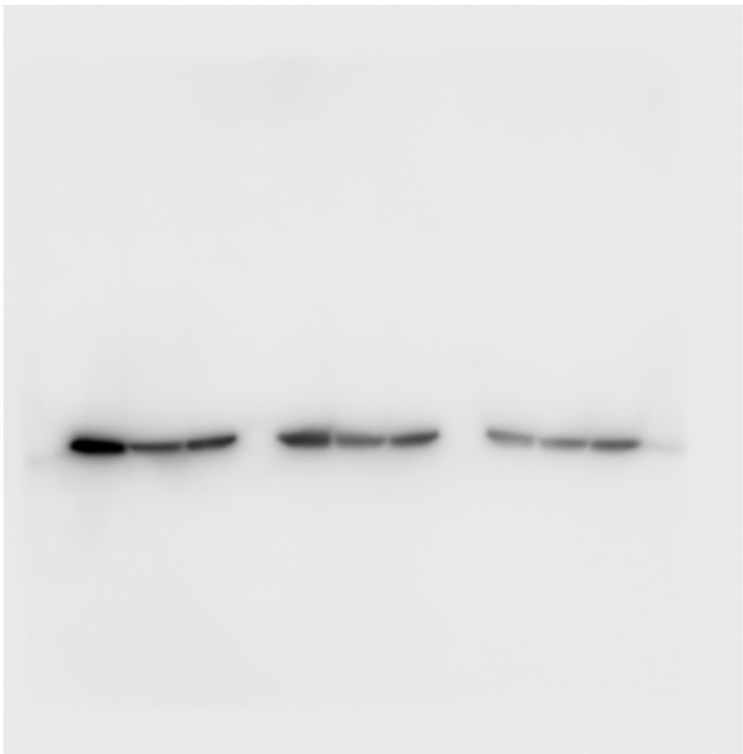

Supplement: Supplementary file 28 [file emmm0007-0078-sd28.pdf]

Fig. 7A

long fragment

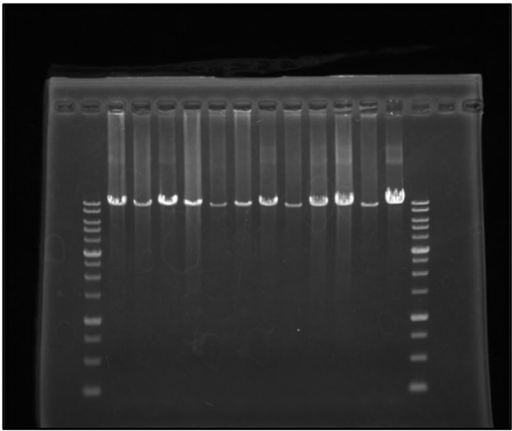

short fragment

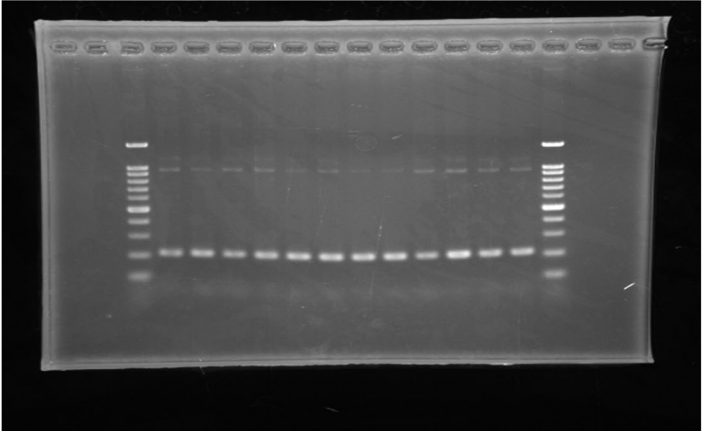

Fig. 7B

long fragment

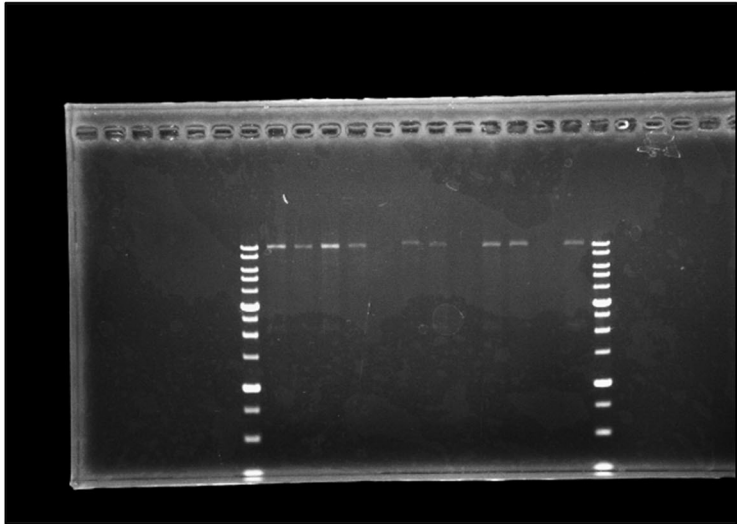

short fragment

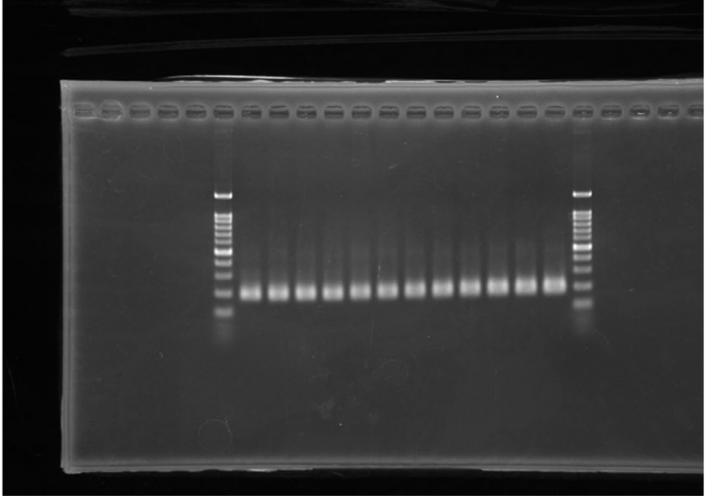

Fig. 7G

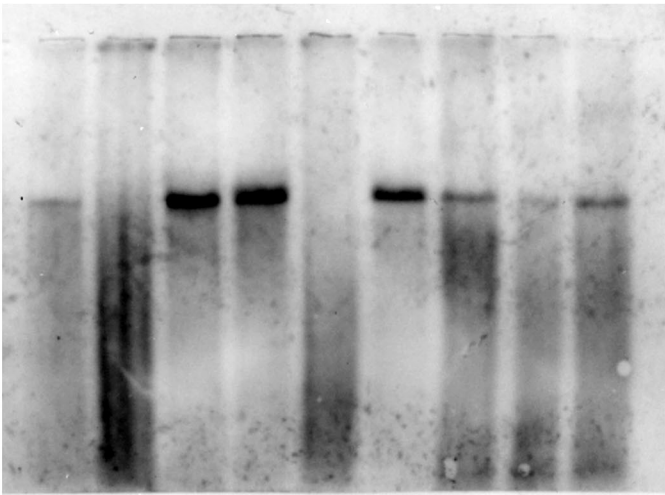

Supplement: Supplementary file 29 [file emmm0007-0078-sd29.pdf]

Fig. 8E

HMGB1

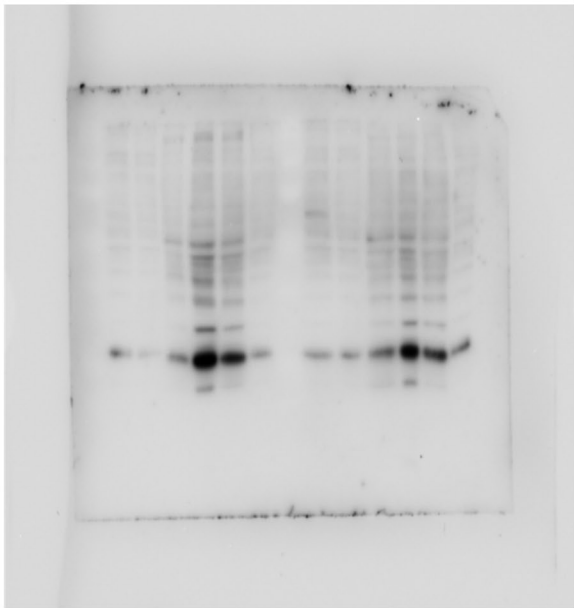

Fig. 8I

$\gamma$ H2AX

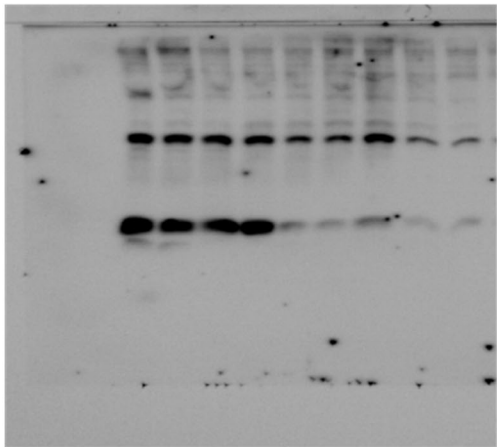

GAPDH

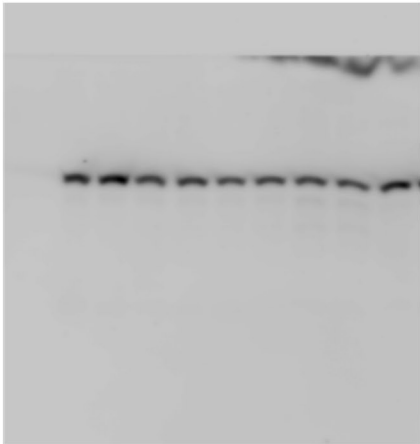

Fig. 8K

long fragment

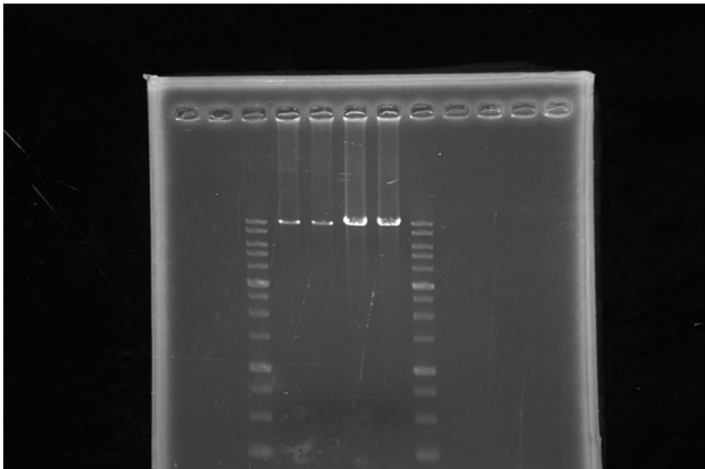

short fragment

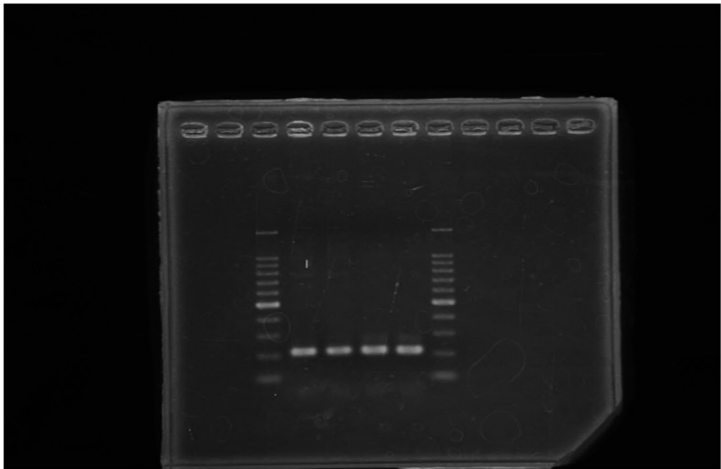

Supplement: Supplementary file 30 [file emmm0007-0078-sd30.pdf]
