# Supplementary material for: HMGB1 facilitates repair of mitochondrial DNA damage and extends the lifespan of mutant ataxin-1 knock-in mice
Source: EMBO Mol Med. 2014 Dec 15;7(1):78–101. doi: 10.15252/emmm.201404392 (PMC4309669; doi:10.15252/emmm.201404392)
Supplement: Supplementary file 14 [file emmm0007-0078-sd14.docx]

**Supplementary Figure and Table Legends**

**Supplementary Figure S1**

***Generation and phenotype analysis of HMGB1 transgenic mice***

(A) The plasmid for the generation of the HMGB1-transgenic mouse. The upstream region (1.9 kb) of the rat neuron specific enolase (NSE) gene was subcloned into the pIRES-hrGFPII (Stratagene) vector as an enhancer/promoter. Rat HMGB1 cDNA was tagged with 3×FLAG. After the internal ribosome entry site (IRES), a humanized recombinant GFP gene was inserted. HMGB1 is highly conserved between the 2 species: mouse and rat HMGB1 are 99.4% identical in their amino acid sequences.

(B) The genomic sequence of transgenic mice was checked by PCR using the primer sets indicated in Supplementary Fig. S1A. Bands of the expected size were amplified from the DNA extracted from the brain tissues of background C57BL/6 mice (WT) and HMGB1 transgenic mice (Tg).

(C) Western blot analysis of whole-brain tissues from background C57BL/6 mice (WT) and HMGB1-transgenic mice (Tg). Flag-tagged HMGB1 (arrow) was expressed in Tg mice (2 weeks) but at a low level. Endogenous proteins are indicated by arrowheads.

(D) Brain tissues (cerebral cortex, hippocampus, hypothalamus, pons, and medulla) from HMGB1-Tg mice (9 weeks) were stained with an anti-FLAG antibody. Although the level of exogenous HMGB1 was low in the western blot of the whole-brain sample (Supplementary Fig. S1C), the exogenous HMGB1 protein was easily detected in NeuN-positive neurons by an anti-FLAG antibody.

(E) Transgenic expression of HMGB1-FLAG was detected with an anti-FLAG antibody. Purkinje cells (arrows) in the cerebellum at 9 weeks were strongly stained with the anti-FLAG antibody.

(F) Similar regions were co-stained with an anti-NeuN or anti-calbindin antibody. A relatively high expression level of HMGB1-FLAG in Purkinje cells and their dendrites was confirmed. NeuN expression was lower in Purkinje cells than in granule cells.

(G) At 9 weeks, no side effects of HMGB1 expression were observed with respect to the thickness of the molecular cell layer and the number of Purkinje cells. Statistical analysis involved Student’s *t* test. WT: non-Tg littermates of HMGB1-Tg mice.

(H) Transgenic co-expression of Ku70 was not so effective as HMGB1 was against the motor dysfunction of mutant-Atxn1-KI mice. One-way ANOVA followed by *post hoc* Tukey’s HSD (honestly significant difference) test revealed that co-expression of HMB1 reversed the pathological phenotype compared to Atxn1-KI or Atxn1-KI-Ku70 mice.

**Supplementary Figure S2**

***HMGB1 does not induce an inflammatory response in the cerebellum***

(A) Mouse cerebellar tissues were immunostained with antibodies against various inflammation markers, such as CD4 (a helper T cell marker), CD8 (a cytotoxic T cell marker), CD11c (a dendritic cell marker), and microglial response factor 1 (a microglial marker). Meanwhile, the activation of these molecules was observed in the brains of positive control mice that were injected peritoneally with 1 mg per kg of body weight of lipopolysaccharide from *Escherichia coli* (Sigma-Aldrich) dissolved in PBS. Brains were dissected 24 h after the injection. ML: molecular layer, GCL: granule cell layer, WM: white matter. Transgenic or AAV-mediated expression of HMGB1 did not induce an inflammatory response. These analyses detected no inflammatory reactions in the brains of HMGB1-overexpressing mice.

(B) Quantitative PCR analysis of inflammatory genes, *Cox-2* and *Il-1β*, in cerebellar tissues of WT, Atxn1-KI, Atxn1-KI;HMGB1-Tg, Atxn1-KI+AAV-GFP, Atxn1-KI+AAV-HMGB1-GFP, and WT+LPS mice at 9 weeks of age. The data were normalized to WT and relative expression levels are shown. According to these data, there was no inflammatory reactions in the brain, in line with Supplementary Figure S2A.

**Supplementary Figure S3**

***HMGB1 reverses nuclear DNA damage in double-transgenic mice***

(A) Nuclear DNA damage in Purkinje cells was examined by immunohistochemical analysis of γH2AX and 53BP1 as markers of DNA damage. All slides from the 3 genotypes of mice were stained simultaneously under identical conditions.

(B) Quantitative analysis of γH2AX signals from the immunohistochemical analysis, where the background signals were subtracted. Student’s *t* test was used for analysis of the differences.

(C) Quantitative analysis of 53BP1 signals from the immunohistochemical analysis, where the background signals were subtracted. Statistical analysis involved Student’s *t* test.

(D) Western blot analysis of nuclear DNA damage using cerebellar tissues from the 3 genotypes of mice.

(E) Quantitative analysis of γH2AX and 53BP1 signals using western blot analyses. Student’s *t* test was used to assess the differences.

(F) Co-staining of γH2AX and HMGB1 was suggestive of a negative relationship between the signal intensity of γH2AX and that of HMGB1 in Purkinje cells.

(G) The quantified signals of γH2AX and HMGB1 per single Purkinje cell were compared (n = 30). Judging by Pearson’s correlation coefficient (r) and the p value, a negative relationship was clearly observed.

**Supplementary Figure S4**

***Mitochondrial HMGB1 contributes to repair of mitochondrial DNA damage and its dynamics but is not involved in mitophagy***

(A) Purkinje cells from the 3 genotypes of mice were co-stained with antibodies to HMGB1 and Cox IV. The overlap between the HMGB1 and Cox IV signals was noticeably reduced in Atxn1-KI mice in comparison with the non-Tg littermates. The overlapping HMGB1 signals were adjusted for total HMGB1 signals; the values are shown in the graph on the right. The percentage of the HMGB1 signal overlapping with the Cox IV signal was remarkably decreased in mutant-Atxn1-KI mice but restored in double-transgenic mice (right graph). The data are presented as mean ± SD; *p < 0.05 in Student’s *t* test, ^#^p<0.05 in one-way ANOVA followed by *post hoc* Turkey’s HSD test.

(B) HMGB1 expression in mitochondria of the 3 mouse genotypes was examined using immunoelectron microscopy. Quantitative analysis of gold/silver particle-positive mitochondria/total mitochondria pointed to downregulation of HMGB1 in mitochondria of Atxn1-KI mice and recovery in Atxn1-86Q-HMGB1 mice.

(C) A negative control experiment with immunoelectron microscopy.

(D) Electron-microscopic analysis of Purkinje cells in the 3 genotypes of transgenic mice. Double membrane vacuoles were not observed in the cell body of Purkinje cells, whereas synaptic regions contained a small number of such vacuoles (arrows).

(E) Quantitative analysis of cytoplasmic autophagosomes (double membrane vacuoles with degraded content) in Purkinje cells did not show any differences among background C57BL/6 mice, Atxn1-KI mice, and the double-transgenic mice. Statistical analysis involved the Bonferroni-Dunn test and Student’s *t* test.

(F) Quantitative analysis of autophagosomes (double membrane vacuoles with degraded content) in synaptic regions. No differences were found among the 3 genotypes of mice. Statistical analysis involved the Bonferroni-Dunn test and Student’s *t* test.

(G) The interaction between Beclin-1 and HMGB1 did not differ in the cerebellar tissues of the 3 groups of mice.

(H) Mitochondrial fission and fusion were downregulated in HeLa cells expressing mutant Atxn1, but this effect was reversed by co-expression of HMGB1. Statistical analysis involved the Bonferroni-Dunn test.

(I) The chloramphenicol (CAP) resistance assay was performed on HeLa cells transfected with 2 types of HMGB1-siRNA. The number of resistant colonies was increased by a HMGB1 knockdown in comparison with control-siRNA. The number of resistant colonies was quantified (lower graph). Statistical analysis involved Student’s *t* test.

(J) Mitochondrial fission and fusion frequencies were quantified (see also Supplementary Video). Statistical analysis involved Student’s *t* test.

(K) Western blot analysis confirmed the knockdown of the HMGB1 protein by shRNA in Supplementary Fig. S4I.

**Supplementary Figure S5**

***Analysis of gene expression profiles of laser-dissected Purkinje cells reveals HMGB1-induced reversion of abnormal gene expression in Atxn1-KI mice***

(A) Laser dissection of Purkinje cells. The middle panel shows the areas marked for laser dissection, and the right panel shows an image after the dissection.

(B) Gene chip analysis of 31,845 genes in the 3 genotypes of transgenic mice identified significantly changed expression of genes in Purkinje cells. The genes in KI/WT and KI-HMGB1/KI are shown in the Venn diagram (top: p < 0.05 in Student’s *t* test, bottom: p < 0.05 in the Tukey-Kramer test). The gene names and a comparison of their expression among the 3 genotypes of mice are shown in Supplementary Table S1.

(C) Significantly changed expression of genes in the comparison of the different genotypes (Atx1-KI versus WT mice; Atx1-KI-Hmgb1 versus Atx1-KI mice) or genes that were rescued in Atx1-KI-Hmgb1 compared to Atx1-KI (shown in the Venn diagram of Supplementary Fig. S5B) were categorized by protein function using the PANTHER classification system (Large-scale gene function analysis with the PANTHER classification system; Mi *et al.,* 2013). Fisher’s exact test was used to detect statistical differences in their proportions of functional groups from all genes of wild-type mice. Functional categories that were significantly upregulated or downregulated (p < 0.01 in Fisher’s exact test) are indicated by red and blue asterisks in pie charts or by red and blue arrows in the list, respectively.

**Supplementary Figure S6**

***Expression changes of representative genes in the 3 genotypes of mice***

(A) Expression profiles of representative genes that may contribute to the HMGB1-related SCA1 pathology based on their expression patterns in the mice of 3 genotypes. The genes are categorized by function or genome. Statistical analysis involved Student’s *t* test, and significant changes (p < 0.05) between WT and Atxn1-Ki mice or Atxn1-KI and Atxn1-KI;HMGB1 mice are marked with asterisks. In parallel, we performed one-way ANOVA followed by *post hoc* Turkey’s HSD (honestly significant difference) test for comparison of the 3 genotypes. The genes with significant expression changes among the 3 genotypes are highlighted in mazenda.

(B) Protein expression levels of ATM and phospho-ATM were examined in the groups mice of the 3 genotypes. ATM and phospho-ATM were upregulated in Atxn1-KI mice and normalized in Atxn1-KI;HMGB1 mice.

(C) The expression profiles of significantly changed autophagy-related genes are shown. Two genes showed an HMGB1-dependent pattern. Statistical analysis involved Student’s *t* test. Asterisks indicate significant changes (p < 0.05). In parallel, we performed one-way ANOVA followed by *post hoc* Turkey’s HSD (honestly significant difference) test for comparison of three genotypes. The genes that underwent significant expression changes among the 3 genotypes are marked with mazenda background.

**Supplementary Figure S7**

***HMGB1-induced improvements in dendritic and dendritic spine morphology of Purkinje cells***

(A) AAV-EGFP was injected into the cerebellar surface through a hole in the skull made by a dental drill. Cerebellar tissue was sliced (100 μm) and visualized using a 2-photon microscopy system FV1000MPE2 (Olympus, Japan). The images were reconstructed and analysed by IMARIS 7.2.2 (Bitplane, Switzerland). Images are shown in 2 orientations.

(B) The dendrite area, dendrite total length, and branch point number of dendrites were analysed by IMARIS. The data are shown as mean ± SD. Statistical analysis involved Student’s *t* test. The results showed abnormal morphology of dendrites of Purkinje cells in Atxn1-KI mice and recovery in Atxn1-KI;HMGB1 mice.

(C) The AAV-EGFP vector was injected into the cerebellar surface, and the dendritic spines of Purkinje cells were monitored using a 2-photon microscopy system, FV1000MPE2. (D) Protrusion (spine) length, maximum diameter, volume, density, and subtypes were analysed by IMARIS. The data are shown as mean ± SD. Statistical analysis involved Student’s *t* test. The results showed abnormal morphology of spines of Purkinje cells in Atxn1-KI mice and recovery in Atxn1-KI;HMGB1 mice. However, the ratio of spine subtypes was not changed.

**Supplementary Figure S8**

***Improvements in motor function and the lifespan of mutant-Atxn1-KI mice after infection with AAV-HMGB1***

(A) Triple transduction methods for producing recombinant AAV vectors are shown. The plasmids contain an expression cassette between the inverted terminal repeats (ITR) of the AAV3 genome. The AAV2 *rep*/AAV1 *vp*-expressing plasmid contains the AAV2 *rep* and AAV1 *vp* genes, which are required for replication and capsid formation. The pHelper plasmid carries the E2A, E4, and VA RNA genes of the adenoviral genome. HEK 293 cells were cotransfected with the vector plasmid, AAV2 *rep*/AAV1 *vp*-expressing plasmid, and pHelper plasmid. AAV2 rep protein recognizes AAV3 ITRs and produces recombinant AAV1/3 vectors carrying expression cassettes between ITRs of the AAV3 genome encapsulated by the AAV1 vp protein. Abbreviations: CMV Pr, human cytomegalovirus immediate-early promoter (CMV promoter) and the human growth hormone first intron; rHMGB1, rat high-mobility group box 1; SV40 poly(A), simian virus 40 polyadenylation signal sequence; WPRE, woodchuck hepatitis virus posttranscriptional regulatory element.

(B) Injection of AAV-GFP and AAV-HMGB1-GFP into the cerebellar surface (at 5 weeks) efficiently transfected cerebellar tissues with AAV. Fluorescence was detected directly by illumination under a fluorescence stereoscopic microscope 4 weeks after the injection. The number of injected mice was 12 for AAV-GFP and 7 for AAV-HMGB1-GFP.

(C) The Rotarod performance at 9 weeks of age (4 weeks after injection) and at 13 weeks of age (8 weeks after injection) revealed AAV-HMGB1-GFP-induced improvement of motor function in Atxn1-KI mice after the onset of symptoms. The data are shown as mean ± SD for days 1−3. Statistical analysis involved Student’s *t* test.

(D) Survival curves of Atxn1-KI mice (blue line), AAV-HMGB1-GFP-injected Atxn1-KI mice (yellow line), AAV-GFP-injected Atxn1-KI mice (grey line), and wild type (green line).

**Supplementary Figure S9**

***Which function HMGB1 performs depends on its subcellular localization***

HMGB1 is essential for DNA damage repair and transcription in the nucleus (Müller *et al.,* 2001; Travers, 2003). When translocated to the cytosol, HMGB1 sequesters Beclin1 from Bcl2 to activate autophagy (Tang *et al.,* 2010). In addition to these previously known functions of HMGB1, the present study revealed that HMGB1 promotes DNA damage repair in mitochondria and affects mitochondrial transcription. Given that autophagy and mitochondrial DNA damage repair mutually support mitochondrial quality control, impairment of both functions of HMGB1 is very harmful for the cell. According to our present results, the autophagy mechanism may not be very active or may not be responsive to the mutant Atxn1 protein in Purkinje cells. Damaged cells (especially necrotic cells) release HMGB1 into the extracellular space and induce inflammatory responses. AAV-delivered HMGB1 (v-HMGB1, green) rescued nuclear and mitochondrial functions without inducing inflammation. In addition to the direct effects of HMGB1 on mitochondrial DNA damage and autophagy, exogenous HMGB1 normalizes the transcription of genes related to mitochondrial/nuclear DNA damage repair and autophagy; these effects indirectly lead to the recovery of these cellular functions.

**Supplementary Table S1**

***Gene expression changes detected by means of microarray analysis***

The list of genes whose expression was changed between wild type C57BL/6 background mice and Atxn1-KI mice (sheet 1: Student’s test, sheet 2: Tukey’s test) or between Atxn1-KI mice and Atxn1-KI;HMGB1 mice (sheet 3: Student’s *t* test, sheet 4: Tukey’s test). The list of rescued genes in the intersection of the Venn diagram in Supplementary Fig. S5B (sheet 5: Student’s *t* test, sheet 6: Tukey’s test).

**Supplementary Table S2**

***Results from the PANTHER analysis***

Fisher’s exact test revealed that the rescued genes according to Student’s test (Supplementary Fig. 5B, top: Venn’s diagram) belonged to specific PANTHER groups: “metabolic” and “primary metabolic” (sheet 1). The list of genes that belong to the “metabolic” and “primary metabolic” groups according to PANTHER analysis (sheets 2 and 3). Fisher’s exact test revealed that the rescued genes according to the Tukey-Kramer test (Supplementary Fig. 5B, lower part of the Venn diagram) belonged to specific PANTHER groups, “metabolic,” “primary metabolic,” and “lipid metabolic process” (sheet 4). The list of genes that belong to “metabolic,” “primary metabolic,” and “lipid metabolic process” groups according to the PANTHER analysis (sheets 5, 6, and 7).

**Supplementary Table S3**

***Results from analysis of enrichment in gene ontology (GO) annotations***

Analysis of enrichment in GO annotations was performed to determine which GO categories were enriched significantly among the affected genes in Atxn1-KI mice compared to wild-type C57BL/6 background mice (sheet 1), the affected genes in Atxn1-KI;HMGB1 mice compared to Atxn1-KI mice (sheet 2), and normalized genes in Atxn1-KI;HMGB1 mice compared to Atxn1-KI mice (sheet 3) according to the Tukey-Kramer test. Fisher’s exact test revealed that the normalized genes (according to the Tukey-Kramer test) belong to specific GO categories: “negative regulation of endocytosis,” “negative regulation of transport,” and “regulation of vesicle-mediated transport.” The list of genes that belong to “negative regulation of endocytosis,” “negative regulation of transport,” and “regulation of vesicle-mediated transport” categories is shown (sheet 4).

**Supplementary Table S4**

***P-values of all statistic analyses***

All the p-values in statistic analyses in Figures and Supplementary Figures are listed.

**Supplementary Videos**

***Mutant Atxn1 reduces the mitochondrial membrane potential and impairs mitochondrial fission and fusion***

Live cell imaging was performed on HeLa cells expressing DsRed (**Supplementary Video 1**), Atxn1-86Q-DsRed (**Supplementary Video 2**) or co-expressing Atxn1-86Q-DsRed and HMGB1-EGFP (**Supplementary Video 3**) while cultured at 37°C and 5% CO_2_. The images were acquired every 2 s for 10 min using a FluoView FV10i-w confocal microscope (Olympus). DsRed at 558 nm and MitoTracker Deep Red at 644 nm were discriminated by means of a Cy5 filter (635 nm). The numbers of mitochondrial fission and fusion events during 10 min in a visual field of 100 μm^2^ were quantified and are presented in Supplementary Fig. S4H. The expression levels of Atxn1 and HMGB1 were assessed by means of DsRed and EGFP signals as shown in Fig. 3A.
