# Supplementary material for: HMGB1 facilitates repair of mitochondrial DNA damage and extends the lifespan of mutant ataxin-1 knock-in mice
Source: EMBO Mol Med. 2014 Dec 15;7(1):78–101. doi: 10.15252/emmm.201404392 (PMC4309669; doi:10.15252/emmm.201404392)
Supplement: Supplementary file 22 [file emmm0007-0078-sd22.pdf]

EMBO Molecular Medicine

HMGB1 facilitates repair of mitochondrial DNA damage and extends the lifespan of mutant ataxin-1 knock-in mice

Hikaru Ito, Kyota Fujita, Kazuhiko Tagawa, Xigui Chen, Hidenori Homma, Toshikazu Sasabe, Jun Shimizu, Shigeomi Shimizu, Takuya Tamura, Shin-ichi Muramatsu and Hitoshi Okazawa

Corresponding author: Hitoshi Okazawa, Tokyo Medical and Dental University

Review timeline:

Submission date:	02 July 2014
Editorial Decision:	29 July 2014
Revision received:	12 October 2014
Editorial Decision:	30 October 2014
Revision received:	11 November 2014
Accepted:	19 November 2014

Transaction Report:

(Note: With the exception of the correction of typographical or spelling errors that could be a source of ambiguity, letters and reports are not edited. The original formatting of letters and referee reports may not be reflected in this compilation.)

Editor: Céline Carret

1st Editorial Decision 29 July 2014

Thank you for the submission of your manuscript to EMBO Molecular Medicine. We have now heard back from the two referees whom we asked to evaluate your manuscript. Although the referees find the study to be of potential interest, they also raise a number of concerns that must be addressed in the next version of your article.

As you will see, both referees found the manuscript of interest but also rather complicated to follow and suggest re-structuring and refocusing the article to improve readability and clarity. In addition, referee 1 requests better figures and referee 2 is concerned about the limited mechanistic insights *in vitro* but also, importantly, *in vivo*. This referee strongly recommends providing more experimental details to improve conclusiveness. As the report is clear and well organised, I would not repeat all issues here but would like to emphasise, that we strongly advise you to address all issues raised for a revision to be further considered.

Please note that it is EMBO Molecular Medicine policy to allow only a single round of revision and that, as acceptance or rejection of the manuscript will depend on another round of review, your responses should be as complete as possible.

EMBO Molecular Medicine has a "scooping protection" policy, whereby similar findings that are published by others during review or revision are not a criterion for rejection. Should you decide to submit a revised version, I do ask that you get in touch after three months if you have not completed it, to update us on the status.

Please also contact us as soon as possible if similar work is published elsewhere. If other work is

published we may not be able to extend the revision period beyond three months.

I look forward to seeing a revised form of your manuscript as soon as possible.

***** Reviewer's comments *****

Referee #1 (Comments on Novelty/Model System):

There is no immediate medical impact. However, the study contributes to the identification of potential treatment targets in SCA1. This is relevant.

Referee #1 (Remarks):

Spinocerebellar ataxia type 1 (SCA1) is a CAG repeat expansion mutation neurodegenerative disorder for which so far there is no effective treatment. The expression of the mutant protein, ATXN1, causes SCA1 probably by changing the interaction with pathways relevant for alternative splicing in the nucleus and several regulators of transcription. Ito and colleagues investigate the influence of high mobility group box protein 1 (HMGB1) on the effects of mutant ataxin 1 in a mouse model of this neurodegenerative disease. They build on previously published evidence in neurons expressing mutant ataxin1 that suggested a role of HMGB1 in maintaining the balance between splicing and transcription. The main aim seems to be to examine the different nuclear functions of HMGB1 and the cytoplasmic roles with a focus on mitochondrial quality control.
The authors report on the creation of a double-transgenic mouse (atxn1-154Q KI-HMGB1). They characterize the pathological phenotype and report on behavioural improvement and prolonged survival compared with the atxn1-154Q model. They then examine how HMGB1 might exert such beneficial effects. They describe corrected DNA damage repair systems and mitochondrial DNA repair. In addition, they find reduced synapse and autophagy gene expression that is improved in the double transgenic mice. Finally, they use adeno-associated virus HMBG1 gene transfer upon which nuclear and mitochondrial abnormalities and survival improve in atxn mutant mice. They conclude that targeting HMGB1 could be a viable approach for treating human spinocerebellar ataxia type 1.
The manuscript is rich in interesting data. In particular, the pronounced improvement of survival of the mice, both the atxn1-154Q KI-HMGB1 double transgenic and the AAV HMGB1 infected, suggests that indeed HMGB1 confers a substantial benefit for atxn mutant mice. Thus, the manuscript adds to the literature and potentially opens new avenues for treatment in a model of SCA1.
However, I got lost many times because of the sheer volume and complexity of the data. In my opinion the manuscript would benefit if it were less convoluted following on from a clear hypothesis. Maybe the authors could consider whether really all the data they present contribute to telling a convincing story. This may also help focus and streamline the discussion so it is easier for the reader to appreciate the authors' work. While the abstract gives a clear overview of the main findings I found the discussion difficult to follow at times.

Other points

- The hypotheses could be stated more clearly in the introduction. They could state more clearly that they went from their previous findings in cells to evaluating the influence on phenotype and survival of HMGB1 in a SCA1 mouse model.
- I understand the limitations of space. However, some of the figures, especially the supplementary figures, are so small that I needed a magnifying glass to appreciate them. Even then it was sometimes difficult to judge what was being shown. This is a pity since it may mean that I missed relevant information.
-Figure 2B. I find it very difficult to make out the immunogold signal and to reliably tell it apart from non-specific staining

Referee #2 (Comments on Novelty/Model System):

The experiments showing that HGMB1 overexpression improves SCA1 mouse phenotypes are convincing, however data showing that HMGB1 might act through mitochondrial DNA damage pathway are less convincing, since most conclusions are based on experiments performed using transfected HeLa cells and these experiments are not always conclusive.
The team has previously published a paper on the topic (Nat Cell Biol 2007) showing that HMGB1 is altered in SCA1 models and over expression of HMGB1 in drosophia has beneficial effects. SCA1 and more generally polyQ disorders are dramatic neurodegenerative diseases with no treatments available. The proposed strategy is therefore interesting regarding medical impact.

Referee #2 (Remarks):

The paper by Ito et al. investigates the effect of overexpressing HMGB1 in SCA1 mice (expressing polyQ-ataxin-1) and shows that HGMB1 overexpression using both HMGB1 transgenic mice and injection of AAV-HMGB1 vectors improves phenotypes of SCA1 mice, including motor function and survival. In addition the study describes an effect of HGMB1 on mitochondrial damage, suggesting that HMGB1 overexpression suppresses mitochondrial DNA damage induced by polyQ-ataxin-1 in SCA1 purkinje neurons, corresponding to cell-type affected in SCA1. The team previously showed that HMGB1 interacts with ataxin-1 and Htt and soluble HMGB1/2 is reduced in neurons expressing polyQ-ataxin-1 or polyQ-Htt (corresponding to mutated proteins in SCA1 and Huntington's diseases, respectively). They further showed that compensatory expression of HMGBs ameliorates polyQ-induced pathology in primary neurons and in Drosophila polyQ models, possibly through inhibition of genotoxic stress signals or transcriptional repression (Qi et al., Nat Cell Biol, 2007). Thus the present study extends this work. Generally, whereas the experiments showing that HGMB1 overexpression improves SCA1 mouse phenotypes are convincing, data showing that HMGB1 might act through mitochondrial DNA damage pathway are less convincing, since most conclusions are based on experiments performed using transfected HeLa cells. In vivo data are missing regarding this point.

Several concerns should be addressed.

- Generally, the manuscript should be shortened and more focused. The Results and Discussion sections are too long. Informative results and discussion should be kept. The structure of the paper could also be improved. For instance the results obtained in HGMB1 transgenic mice and in mice injected with HGMB1-AAV vector could be described in parallel, which would prevent repetitions. The results are often discussed in the Results section instead of the Discussion section. The manuscript should be edited. Pages should be numbered. Several quantifications and statistics are based on a number of unspecified measurements. The number of measurement should be specified systematically, including in sup figures (for example see Fig 5B, C, D)

- Fig. 1. The authors should specify how many HMGB1 transgenic mouse lines they have generated and used in the study. It seems they only used one. Do HMGB1 transgenic mice present some phenotypes?

- Fig 2B. Control are missing to show the staining is specific. What cells are shown in Fig. 2B? (purkinje neurons?). How did they quantify in Fig. S3B? Did the authors also perform IHF to show HGMB1 is in mitochondria of purkinje neurons? What tissues or cells were used in Fig. 2D?

- Fig 3D. SDH and COX (mistake: CCO instead of COX in legend figure?) stainings are not too convincing (mild effects, not quantitated).

- Fig 4. The complete gels should be shown. Could the authors use quantitative RT-PCR to quantify more accurately their results? Could the authors exclude that changes in PCR efficiency between WT/atxn1 KI-HMGB1 and atxn1 KI do not reflect mtDNA conformational changes rather than increase in mtDNA damage, since HMGB proteins affects DNA conformation. What types of DNA damage are expected due to decreased HGMB1, SSB, DDB...? Could the authors investigate this?

Is mitochondrial genome copy number different between WT, Atxn1 KI and Atxn1-HMGB1 mice? This should be checked.

Regarding high throughput sequencing in Fig. 4D. How many times was the experiment replicated? If the results are based on one experiment only, could we exclude technical artifacts resulting from variations in sonication or in sample quality and preparation? The data could be presented using box plots, which would be easier to read. As presented in Fig. 4D, it's not clear that read length is shifted to the shorter fraction. What is the time of reads?
Why is average and mitochondrial read length longer in atxn1 mice compared to WT or Atxn1-HMGB1 mice (Fig. 4E)?
Is the mutation frequency statistically different between atxn1 mice and WT or atxn1-HMGB1 mice

- Fig. 5. Why did the author use L929 cells for X-ray irradiation? What types of damage do they induce. Why did the only check for 8-OHdG (methods regarding experiment in Fig. 5D are missing)?
How many times was the experiment replicated in Fig. 5B? Regarding statistical analyses, it is sufficient to keep the one-way anova test followed by post hoc test analysis (student's t test analysis should be removed). Same comments with respect to Fig. 5C.

It would be wise to compare the effect of decreased HMGB1 in atxn1 KI mice on mtDNA and nuclear DNA to evaluate respective contributions to SCA1 phenotype.

- Fig. 6A. Expression of HMGB1 and atxn1 is highly variable between transfected cells. How representative are the pictures shown?
Fig6B. Many bands are detected following HGMB1 ChIP. The PCR does not appear to be very specific. alternatively could the additional bands correspond to broken fragments bound with HGMB1? Overall, the experiment is not conclusive.Is it possible to sonicate mtDNA and perform a more quantitative PCR? How many times was the experiment replicated?

- Sup Fig. S4. Statistical analyses of microarray data should be corrected for multiple testing. Genes significantly deregulated after this correction should be considered.
Why did the author use only PANTHER for pathway enrichment analysis (and not GO for example). Functional enrichment analyses should be re-done using genes deregulated after correction for multiple testing and additional methods (GO...).

-The part "HMGB1 rescues the expression of mito DNA repair genes " should be shortened to few sentences. In addition, rescue is not specific to mito genes: HMGB1 does not rescue all mito genes and rescue of non mito genes also occurs. Moreover, changes go in both direction (increased and decreased) and the consequence cannot be predicted.
Conclusions should therefore be tone down, this part is too speculative and vague. Too much discussion in the results part (ex: "we found recently that mutant atxn1 directly interacts with Rpa1 and BRCA1 involved in DNA double strand break repair by homologous recombination. Therefore... Meanwhile..").
Fxn is not a DNA repair protein.

- Same comments regarding the next part "HMGB1 rescues synapse-related and autophagy-related gene expression". It should be shortened, more focused and less speculative or toned down.
For example: "the decreased expression of these genes could underlie the decreased thickness of the molecular layer....". The authors focus on the expression of 3 genes only, thus "underlie" should be changed for "contribute". Also, levels of corresponding proteins (Cbl1, Dab1 and Tln2) could be determined.

Fig. 7 and AAV.
The sentence: "we investigated a novel gene therapy for SCA1 using HMGB1" should be modified. We cannot talk about therapy at that point (given side effects associated with HMGB1 overexpression: cancer, inflammation).
Details of the constructs should be specified in Methods, not in results (it's not clear why they mix AAV1 and AAV3 subtypes). Is specific targeting of purkinje neurons expected. This should be commented.
The injection mode should be justified in Methods. The sentence "...we used direct injection into the

posterior fossa.... because of an expected side effect that we discuss in the Discussion" should be removed.

Western-blots using HMGB1 antibody could be performed to verify that the increase of HMGB1 mRNA in atxn-1 mice overexpressing HMGB1 associates with increased HMGB1 protein.

Fig. 7K. The quality of the experiment is not optimal. How did the authors quantified data from this experiment?

- Experiments aimed to verify inflammation in mice overexpressing HGMB1 could be improved, especially since the authors claim that the strategy could be used for gene therapy. Is it possible to dose inflammatory molecules or check expression of inflammatory genes?

- There is no reference to Fig S7 in the manuscript.

- The absence of effect of HMGB1 overexpression on autophagy should be tone down, since the authors do not see autophagic vacuoles in atxn1 mice (Fig S3), nor do they see interaction between beclin-1 and HMGB1 (they should be cautious with negative results).

1st Revision - authors' response 12 October 2014

Referee #1 (Comments on Novelty/Model System):

There is no immediate medical impact. However, the study contributes to the identification of potential treatment targets in SCA1. This is relevant.

Referee #1 (Remarks):

Spinocerebellar ataxia type 1 (SCA1) is a CAG repeat expansion mutation neurodegenerative disorder for which so far there is no effective treatment. The expression of the mutant protein, ATXN1, causes SCA1 probably by changing the interaction with pathways relevant for alternative splicing in the nucleus and several regulators of transcription. Ito and colleagues investigate the influence of high mobility group box protein 1 (HMGB1) on the effects of mutant ataxin 1 in a mouse model of this neurodegenerative disease. They build on previously published evidence in neurons expressing mutant ataxin1 that suggested a role of HMGB1 in maintaining the balance between splicing and transcription. The main aim seems to be to examine the different nuclear functions of HMGB1 and the cytoplasmic roles with a focus on mitochondrial quality control.

The authors report on the creation of a double-transgenic mouse (atxn1-154Q KI-HMGB1). They characterize the pathological phenotype and report on behavioural improvement and prolonged survival compared with the atxn1-154Q model. They then examine how HMGB1 might exert such beneficial effects. They describe corrected DNA damage repair systems and mitochondrial DNA repair. In addition, they find reduced synapse and autophagy gene expression that is improved in the double transgenic mice. Finally, they use adeno-associated virus HMBG1 gene transfer upon which nuclear and mitochondrial abnormalities and survival improve in atxn mutant mice. They conclude that targeting HMGB1 could be a viable approach for treating human spinocerebellar ataxia type 1.

The manuscript is rich in interesting data. In particular, the pronounced improvement of survival of the mice, both the atxn1-154Q KI-HMGB1 double transgenic and the AAV HMGB1 infected, suggests that indeed HMGB1 confers a substantial benefit for atxn mutant mice. Thus, the manuscript adds to the literature and potentially opens new avenues for treatment in a model of SCA1.

>>> Thank you very much for kind evaluation of our work.

However, I got lost many times because of the sheer volume and complexity of the data. In my opinion the manuscript would benefit if it were less convoluted following on from a clear hypothesis. Maybe the authors could consider whether really all the data they present contribute to telling a convincing story. This may also help focus and streamline the discussion so it is easier for the reader to appreciate the authors' work. While the abstract gives a clear overview of the main findings I found the discussion difficult to follow at times.

>>> Thank you very much again for the kind suggestion to shape up the manuscript. We deleted previous Fig 6A from this version. We considered to volume down other figures, but since the second reviewer requested some additional experiments, at this moment we tried to prevent increasing the volume by this revision. Naturally, we also followed the suggestion from the first reviewer, and tried to focus and clarify the line of discussion. We deleted non-essential discussion and substantially shorten Discussion.

Other points

- The hypotheses could be stated more clearly in the introduction. They could state more clearly that they went from their previous findings in cells to evaluating the influence on phenotype and survival of HMGB1 in a SCA1 mouse model.

>>> We changed the order of sentences and tried to make more focused introduction following the advice of the reviewer.

- I understand the limitations of space. However, some of the figures, especially the supplementary figures, are so small that I needed a magnifying glass to appreciate them. Even then it was sometimes difficult to judge what was being shown. This is a pity since it may mean that I missed relevant information.

>>> We enlarged font sizes and tried to make explanation easy to understand.

-Figure 2B. I find it very difficult to make out the immunogold signal and to reliably tell it apart from non-specific staining

>>> We performed control experiments with normal IgG

Referee #2 (Comments on Novelty/Model System):

The experiments showing that HGMB1 overexpression improves SCA1 mouse phenotypes are convincing, however data showing that HMGB1 might act through mitochondrial DNA damage pathway are less convincing, since most conclusions are based on experiments performed using transfected HeLa cells and these experiments are not always conclusive.

The team has previously published a paper on the topic (Nat Cell Biol 2007) showing that HMGB1 is altered in SCA1 models and over expression of HMGB1 in drosophila has beneficial effects.

SCA1 and more generally polyQ disorders are dramatic neurodegenerative diseases with no treatments available. The proposed strategy is therefore interesting regarding medical impact.

Referee #2 (Remarks):

The paper by Ito et al. investigates the effect of overexpressing HMGB1 in SCA1 mice (expressing polyQ-ataxin-1) and shows that HGMB1 overexpression using both HMGB1 transgenic mice and injection of AAV-HMGB1 vectors improves phenotypes of SCA1 mice, including motor function and survival. In addition the study describes an effect of HGMB1 on mitochondrial damage, suggesting that HMGB1 overexpression suppresses mitochondrial DNA damage induced by polyQ-ataxin-1 in SCA1 purkinje neurons, corresponding to cell-type affected in SCA1. The team previously showed that HMGB1 interacts with ataxin-1 and Htt and soluble HMGB1/2 is reduced in neurons expressing polyQ-ataxin-1 or polyQ-Htt (corresponding to mutated proteins in SCA1 and Huntington's diseases, respectively). They further showed that compensatory expression of HMGBs ameliorates polyQ-induced pathology in primary neurons and in Drosophila polyQ models, possibly through inhibition of genotoxic stress signals or transcriptional repression (Qi et al., Nat Cell Biol, 2007). Thus the present study extends this work. Generally, whereas the experiments showing that HGMB1 overexpression improves SCA1 mouse phenotypes are convincing, data showing that HMGB1 might act through mitochondrial DNA damage pathway are less convincing, since most conclusions are based on experiments performed using transfected HeLa cells. In vivo data are missing regarding this point.

>>> Thank you very much for kind evaluation and critical suggestion. In response to the comment, we newly performed in vivo experiment, in which we observed DNA damage repair of mitochondrial genome and nuclear genome after irradiation (New Fig 6). Old Fig 4 and 5 were combined to new Fig 4.

Several concerns should be addressed.

- *Generally, the manuscript should be shortened and more focused. The Results and Discussion sections are too long. Informative results and discussion should be kept. The structure of the paper could also be improved.*

>>> We deleted a large volume of result and discussion sections that were non-essential.

For instance the results obtained in HGMB1 transgenic mice and in mice injected with HGMB1-AAV vector could be described in parallel, which would prevent repetitions.

>>> We appreciate this suggestion from the reviewer. Following the advice we actually tried to describe transgenic data and viral vector data in parallel. However, we noticed that it made the structure of figure more complex because pathological and mechanistic analyses were based on KI and Tg mice, while final output of this paper, suggestion for a possible therapeutics, is made with viral vector. Therefore, after carefully considering the two forms of manuscript, we decided to follow mostly the previous way of presentation. However, we tried our best to delete unnecessary repetitions following the advice of the reviewer.

The results are often discussed in the Results section instead of the Discussion section. The manuscript should be edited.

>>> Following the advice, we moved these discussions in “Results” to the last paragraph of page 19 in “Discussion” (page 19-20).

Pages should be numbered.

>>> We added page numbers.

Several quantifications and statistics are based on a number of unspecified measurements. The number of measurement should be specified systematically, including in sup figures (for example see Fig 5B, C, D).

>>> We systematically revealed the numbers in Fig and Sup Fig.

- Fig. 1. The authors should specify how many HMGB1 transgenic mouse lines they have generated and used in the study. It seems they only used one. Do HMGB1 transgenic mice present some phenotypes?

>>> It is exactly true that we only used one line. HMGB1 Tg mice did not show any phenotype in behavioural and pathological examinations.

- Fig 2B. Controls are missing to show the staining is specific. What cells are shown in Fig. 2B? (purkinje neurons?). How did they quantify in Fig. S3B? Did the authors also perform IHF to show HGMB1 is in mitochondria of purkinje neurons? What tissues or cells were used in Fig. 2D?

>>> We added control in Sup Fig S4C. Previous presentation was inappropriate because we showed non-Purkinje cells, and in this revision we showed only Purkinje cells in left upper panel of Fig 2B. In Sup Fig S3B (new Sup Fig 4E), we counted all mitochondria in five Purkinje cells and calculated the ratios. In Fig 2D, whole brain tissues were used (written in the figure).

- Fig 3D. SDH and COX (mistake: CCO instead of COX in legend figure?) stainings are not too convincing (mild effects, not quantitated).

>>> We showed quantification graphs.

- Fig 4. The complete gels should be shown. Could the authors use quantitative RT-PCR to quantify more accurately their results? Could the authors exclude that changes in PCR efficiency between WT/atxn1 KI-HMGB1 and atxn1 KI do not reflect mtDNA conformational changes rather than increase in mtDNA damage, since HMGB proteins affects DNA conformation.

>>> We showed complete gels in Fig 4. As the length of DNA used for qPCR is limited, it is technically impossible to quantify 10kb band in qPCR. Sample preparation includes Proteinase K, NP, Tween-20 treatments which definitely exclude HMGB1 proteins. Also PCR reaction condition could exclude conformational issue.

What types of DNA damage are expected due to decreased HGMB1, SSB, DDB...? Could the authors investigate this?

>>> Basically the assays in Fig 4 reflect DDSB(DDB). All types of DNA damage finally leads to DDSB.

We would not go into additional experiments, because it is the basic science issue beyond the scope of this work, and additional experiments will elongate this paper longer and more complex, which had been already extremely long and difficult to follow (Editor, would you please give us advice about it?).

Is mitochondrial genome copy number different between WT, Atxn1 KI and Atxn1-HMGB1 mice? This should be checked.

>>> We performed Southern blot of mitochondrial genome (Fig 7G), which beautifully showed the decrease of intact mitochondrial genome. While the total amount of signals including the DNA smear are relatively near.

Regarding high throughput sequencing in Fig. 4D. How many times was the experiment replicated? If the results are based on one experiment only, could we exclude technical artifacts resulting from variations in sonication or in sample quality and preparation?

>>> The experiment was repeated with three independently different samples.

The data could be presented using box plots, which would be easier to read. As presented in Fig. 4D, it's not clear that read length is shifted to the shorter fraction.

>>> Following the advice, we changed the presentation in Fig 4D.

What is the time of reads?

>>> Time of reads is the same as read count. We changed it in this version. In sequencing with ion PGM, multiple steps affect the read count. Experimental read counts are smaller than expected number by the company, the read counts become smaller when mapped to nuclear and mitochondria genome database (large mutations might be excluded in this step, if it occurs), and the read counts become further smaller by quality check. So the most important parameters in Fig 4E are average read length and mutation ratio.

Why is average and mitochondrial read length longer in atxn1 mice compared to WT or Atxn1-HMGB1 mice (Fig. 4E)?

>>> Average length of read is shorter in KI mice. On the other hand, "mitochondrial read length" does not have to be shorter. The description "mitochondrial read length" was confusing, and it should be changed to "total read length" (which is integrated read length of multiple short fragments). Total read length is dependent on multiple factors. For sequencing with Ion PGM, after preparation of DNA, adaptor sequence was attached. Adaptor-linked DNA was amplified by PCR in emulsion to several million copies, and then they were used for ion PGM sequencing. Therefore, shorter "average length of read" could be longer "total read length".

Is the mutation frequency statistically different between atxn1 mice and WT or atxn1-HMGB1 mice

>>> We showed the statistical difference in mutation frequency in Fig 4E.

- *Fig. 5. Why did the author use L929 cells for X-ray irradiation? What types of damage do they induce. Why did the only check for 8-OHdG (methods regarding experiment in Fig. 5D are missing)?*

>>> As you know, IR induces direct and indirect DNA damages. To confirm the extents of DNA damage in three transfection groups were similar, the 8-OHdG quantification, which evaluates indirect DNA damage sensitively, was performed. Regarding the usage of L929 cells, we tried several lines of cells including Hela cells to amplify a longer DNA fragment (10kb) for DNA damage assay and we constantly succeeded only in L929 cells. The types of DNA damage evaluated by the DNA damage assay was mostly DDSB, which is the final outcome of direct and indirect DNA damages.

How many times was the experiment replicated in Fig. 5B? Regarding statistical analyses, it is sufficient to keep the one-way anova test followed by post hoc test analysis (student's t test analysis should be removed). Same comments with respect to Fig. 5C.

>>> N=4. We removed Student's t-test as requested in Fig 5B and 5C.

It would be wise to compare the effect of decreased HMGB1 in atxn1 KI mice on mtDNA and nuclear DNA to evaluate respective contributions to SCA1 phenotype.

>>> We performed the comparison experiments with in vivo IR (Fig 6).

- *Fig. 6A. Expression of HMGB1 and atxn1 is highly variable between transfected cells. How representative are the pictures shown?*

>>> We agree the quality of this experiment was not high. Since we are requested to delete non-essential data from this paper by the first reviewer, we decided not to use Fig. 6A.

Fig6B. Many bands are detected following HGMB1 ChIP. The PCR does not appear to be very specific. alternatively could the additional bands correspond to broken fragments bound with HGMB1? Overall, the experiment is not conclusive. Is it possible to sonicate mtDNA and perform a more quantitative PCR? How many times was the experiment replicated?

>>> We repeated the experiment and obtained much better result (new Fig 6A middle panel).

- *Sup Fig. S4. Statistical analyses of microarray data should be corrected for multiple testing. Genes significantly deregulated after this correction should be considered.*

>>> In Sup Fig 4, we performed multiple testing (Tukey-Kramer test) and changed the numbers in Venn Gram, although we believe the suggestion is too automatic from the aspect of statistics. We evaluated the %recovery of changed genes, and for the purpose two groups comparison is suitable. We presented both cases in Sup Fig 4B and C. We deleted the lower list in Sup Fig 4C because it was redundant with Sup Table 2.

Why did the author use only PANTHER for pathway enrichment analysis (and not GO for example). Functional enrichment analyses should be re-done using genes deregulated after correction for multiple testing and additional methods (GO...).

>>> We performed GO analysis and the conclusion was basically the same.

© EMBO

-The part "HMGB1 rescues the expression of mito DNA repair genes " should be shortened to few sentences. In addition, rescue is not specific to mito genes: HMGB1 does not rescue all mito genes and rescue of non mito genes also occurs. Moreover, changes go in both direction (increased and decreased) and the consequence cannot be predicted.

Conclusions should therefore be tone down, this part is too speculative and vague. Too much discussion in the results part (ex: "we found recently that mutant atxn1 directly interacts with Rpa1 and BRCA1 involved in DNA double strand break repair by homologous recombination. Therefore... Meanwhile..").

Fxn is not a DNA repair protein.

>>> We shortened the part as requested. The comment about non-mitochondrial repair genes is exactly right. Regarding the orientation of changes, most of the repair genes are well known to trigger DNA damage signal and to promote death in some situation (although the details of such switching functions are not exactly known). Therefore, it does have some meaning in either direction if the abnormal changes are normalized.

>>> However, taking the advice, we tone down and shorten the section substantially. We also changed the title of mitochondria repair genes from "rescue" to "improve", and changed the title of synapse-related genes from "rescue" to "affect".

- Same comments regarding the next part "HMGB1 rescues synapse-related and autophagy-related gene expression". It should be shortened, more focused and less speculative or toned down.

For example: "the decreased expression of these genes could underlie the decreased thickness of the molecular layer....". The authors focus on the expression of 3 genes only, thus "underlie" should be changed for "contribute". Also, levels of corresponding proteins (Cbl1, Dab1 and Tln2) could be determined.

>>> We substantially shortened the section, and deleted unnecessary speculations. We did not go into the protein level because this paper basically handles gene expression and not subsequent biological processes, and because another reviewer requested us to delete marginal data from this paper. Instead, we substantially tone-downed the possible causative meaning of these genes.

>>> We changed the title of synapse-related genes from "rescue" to "affect". We stated that the meaning of change in autophagy genes is unclear. Moreover, the section about synapse-related molecule in "Discussion" was also completely deleted.

Fig. 7 and AAV.

The sentence: "we investigated a novel gene therapy for SCA1 using HMGB1" should be modified. We cannot talk about therapy at that point (given side effects associated with HMGB1 overexpression: cancer, inflammation).

>>> We changed the sentence and description.

Details of the constructs should be specified in Methods, not in results (it's not clear why they mix AAV1 and AAV3 subtypes). Is specific targeting of purkinje neurons expected. This should be commented.

>>> The details of construction of AAV vector had been described in Method section "AAV vector construction" of previous version. There have been technical efforts to version up AAV vectors by switching the components, which actually led to better vectors. We commented about relatively specific expression in the Method.

The injection mode should be justified in Methods. The sentence "...we used direct injection into the posterior fossa.... because of an expected side effect that we discuss in the Discussion" should be removed.

>>> We removed the sentence as recommended.

Western-blots using HMGB1 antibody could be performed to verify that the increase of HMGB1 mRNA in atxn-1 mice overexpressing HMGB1 associates with increased HMGB1 protein.

>>> We added the data in new Fig 7E.

Fig. 7K. The quality of the experiment is not optimal. How did the authors quantified data from this experiment?

>>> We performed the experiment again, obtained better data and quantified (Fig 7K).

- Experiments aimed to verify inflammation in mice overexpressing HGMB1 could be improved, especially since the authors claim that the strategy could be used for gene therapy. Is it possible to dose inflammatory molecules or check expression of inflammatory genes?

>>> We performed qPCR for inflammatory genes with cerebellar tissues, and showed the results in new Sup Fig 2B.

- There is no reference to Fig S7 in the manuscript.

>>> Previous Fig S7 was renamed as new Fig S9. It was referred in multiple paragraphs in Discussion.

- The absence of effect of HMGB1 overexpression on autophagy should be tone down, since the authors do not see autophagic vacuoles in atxn1 mice (Fig S3), nor do they see interaction between beclin-1 and HMGB1 (they should be cautious with negative results).

>>> We toned down as suggested (page 8, last paragraph). In Discussion, we also referred the recover of the two autophagy genes in microarray analysis suggesting a possible involvement of autophagy (page 20).

2nd Editorial Decision 30 October 2014

Thank you for the submission of your revised manuscript to EMBO Molecular Medicine. We have now received the enclosed reports from the referees that were asked to re-assess it. As you will see the reviewers are now globally supportive and I am pleased to inform you that we will be able to accept your manuscript pending the following final amendments:

Please carefully address the last set of comments from both referees. As you will see, they both recommend a thorough proof-reading, shortening sentences and the results section (ref.1) and overall improve the structure of the paper. In addition, please make sure that all figures and sub

panels are called for in the results section, not in the discussion.
Please submit your revised manuscript within two weeks. I look forward to seeing a revised form of your manuscript as soon as possible.

***** Reviewer's comments *****

Referee #1 (Comments on Novelty/Model System):

The immediate medical impact is low. However, the results of this study may contribute to identifying targets for treatment of SCA1. This is relevant.

Referee #1 (Remarks):

In response to this reviewer's comments the authors have improved their manuscript. The introduction now states the aims of this paper more clearly, and the discussion has been pruned. However, I still think that the results section is far too long. In addition, the structure of the manuscrupt could still be improved to make for a simpler read.
The most convincing results are those of improved survival and phenotype of mice with restoration of HMBI expression. The author have added in vivo data on mtDNA damage and DNA damage to boost the HeLa cell data.

Minor points

-The discussion is full of reference to figures and supplementary figures. If necessary such reference, in my opinion, should take place in the results section
-There are many typos throughout the manuscript. While overall the English is acceptable, at least in some instances, the English would also benefit from some editing.

Referee #2 (Remarks):

- Figure 7 is added to the new manuscript but not described in the results section. Figure 5F is also not described. Figure S3H seems to be missing.
-Figure 4A: what is the fragment around 1000bp?
-p13, lines 12-16, it is unclear why there is some discrepancy between the 2 studies
- p13, the paragraph starting with "We further analyzed..." could be shorten, since the results do no show functional enrichments.
-p13: sub-title should be "HMGB1 improves the expression of SOME mitochondrial genes. Same comments p14. Subtitle should be :HMGB1 affects expression of SOME synapse-related genes. The effect cannot be generalized to all of mito or synapse-related genes, since these functions are not enriched in KI or in HMGB1 KI mice.
- Some editing has to be done. For instance, p.10, 2nd paragraph, check the 3rd sentence ("As expected....").

2nd Revision - authors' response 11 November 2014

Referee #1 (Comments on Novelty/Model System):

The immediate medical impact is low. However, the results of this study may contribute to identifying targets for treatment of SCA1. This is relevant.

Referee #1 (Remarks):

In response to this reviewer's comments the authors have improved their manuscript. The introduction now states the aims of this paper more clearly, and the discussion has been pruned. However, I still think that the results section is far too long. In addition, the structure of the manuscript could still be improved to make for a simpler read.

>> Following the comment, we substantially shortened the Result section. We reduced 2 pages from Result section by compacting expressions. But we had to add a new section for Figure 7 (which was lost in the previous version, as the reviewer pointed out) and move a part from Discussion to Result section. Taken together, 1 page was reduced from Result.

The most convincing results are those of improved survival and phenotype of mice with restoration of HMBI expression. The author have added in vivo data on mtDNA damage and DNA damage to boost the HeLa cell data.

>> Thank you very much.

Minor points

-The discussion is full of reference to figures and supplementary figures. If necessary such reference, in my opinion, should take place in the results section

>> We moved a part of Discussion to Result section. Also we shortened the Discussion section.

-There are many typos throughout the manuscript. While overall the English is acceptable, at least in some instances, the English would also benefit from some editing.

>> We corrected the typos and also received editing by a professional English editor.

Referee #2 (Remarks):

- *Figure 7 is added to the new manuscript but not described in the results section.*

>> We described the result of Figure 7 in Result section.

-Figure 5F is also not described. Figure S3H seems to be missing.

>> We described about Figure 5F briefly in Result section. Fig S3H has been moved to Fig S4I.

-Figure 4A: what is the fragment around 1000bp?

© EMBO

>> We consider that it is one of the non-specific bands, and not related to the long band. However, since we obtained a better result by repeating the PCR experiment from the same samples, we replaced the figure and the source data with new ones. We upload the new Fig 4 and the new source data.

-p13, lines 12-16, it is unclear why there is some discrepancy between the 2 studies

>> PATHER and GO have different genes in their categories with homologous terms. It is the primary reason. Also both methods do not retrieve absolute conclusions. Instead they just suggest preliminary expectations.

- p13, the paragraph starting with "We further analysed..." could be shorten, since the results do no show functional enrichments.

>> We changed the part following the recommendation.

-p13: sub-title should be "HMGB1 improves the expression of SOME mitochondrial genes. Same comments p14. Subtitle should be :HMGB1 affects expression of SOME synapse-related genes. The effect cannot be generalized to all of mito or synapse-related genes, since these functions are not enriched in KI or in HMGB1 KI mice.

>> We changed subtitles following the recommendation.

- Some editing has to be done. For instance, p.10, 2nd paragraph, check the 3rd sentence ("As expected....").
