# Supplementary material for: HMGB1 facilitates repair of mitochondrial DNA damage and extends the lifespan of mutant ataxin-1 knock-in mice
Source: EMBO Mol Med. 2014 Dec 15;7(1):78–101. doi: 10.15252/emmm.201404392 (PMC4309669; doi:10.15252/emmm.201404392)
Supplement: Supplementary file 24 [file emmm0007-0078-sd24.pdf]

Supplementary source data: original scans of gels

Fig. 2A

Nuclear cytosolic

Mitochondrial

HMGB1

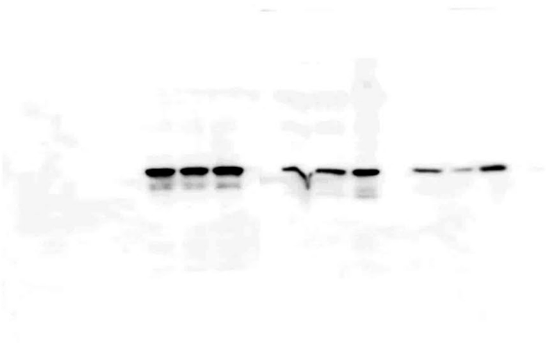

Nuclear cytosolic

HP1α

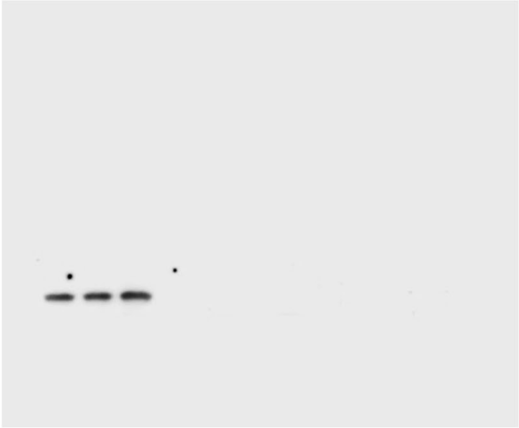

α-tubulin

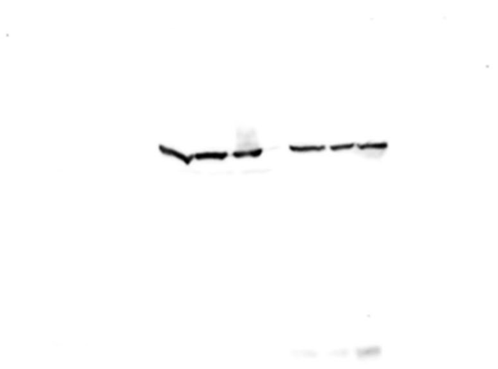

Mitochondrial

Mitochondrial with marker

α-tubulin

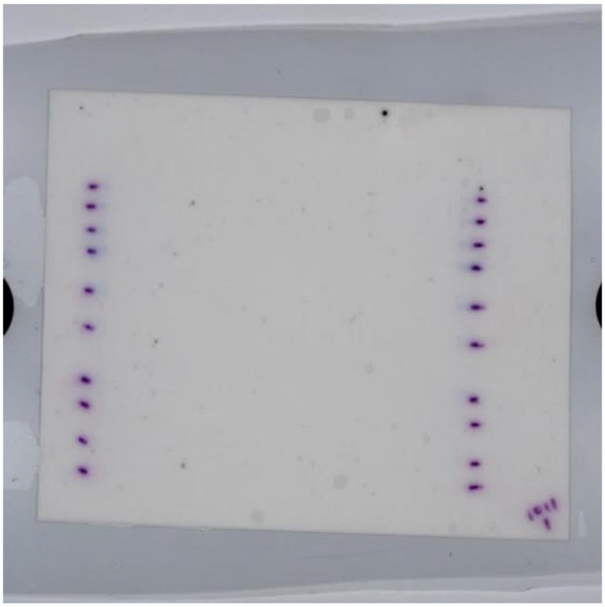

Supplementary source data: original scans of gels

Fig. 2A

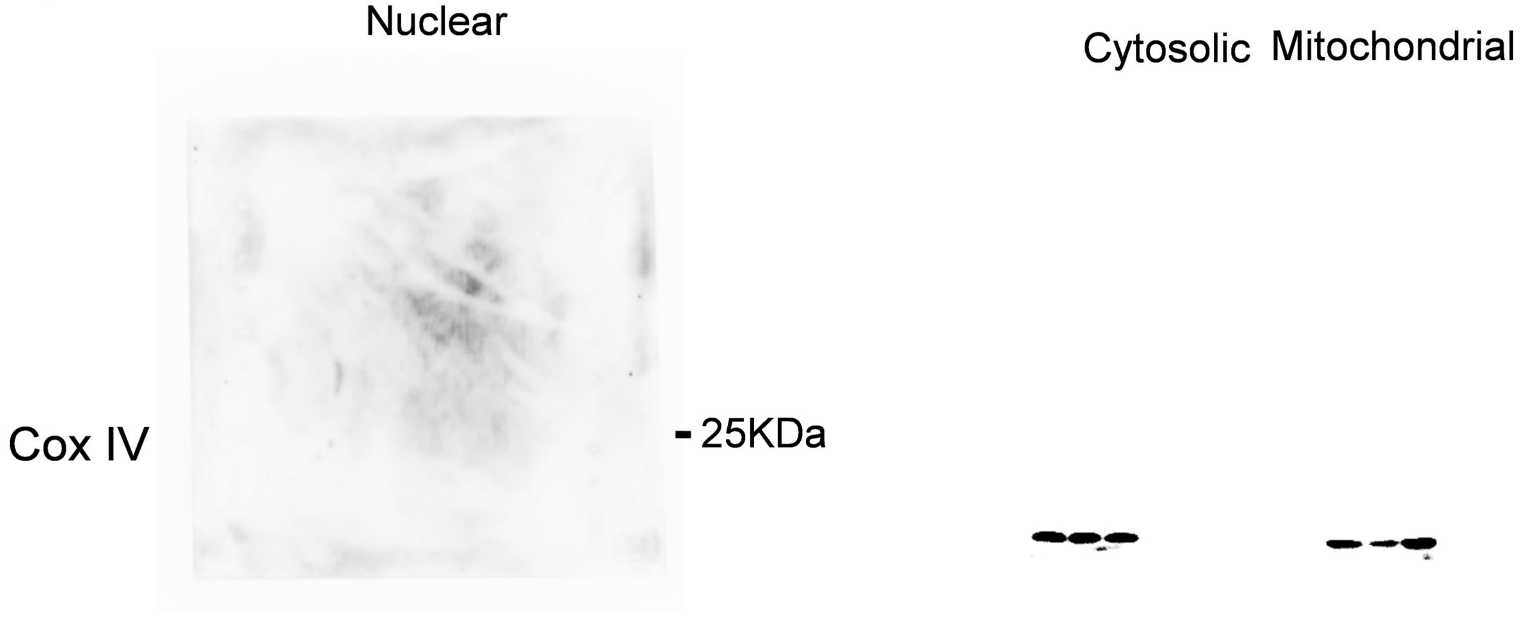

Fig. 2C

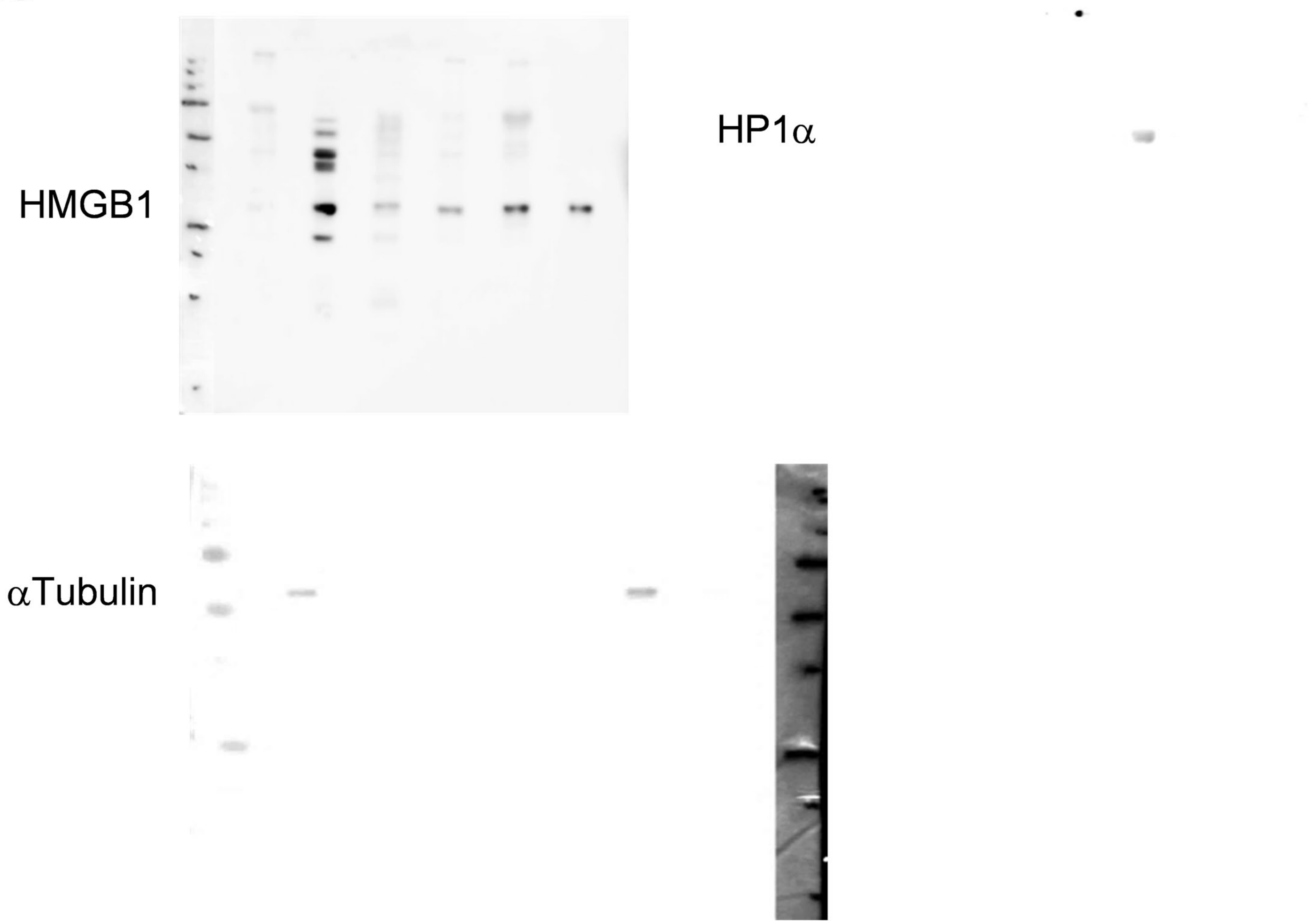

Supplementary source data: original scans of gels

Fig. 2C

Cox IV (upper)

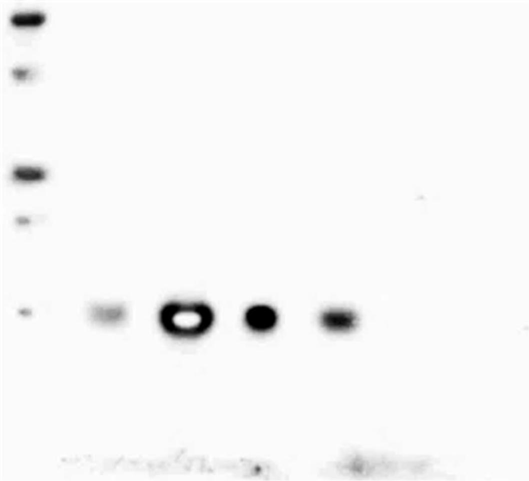

Cox IV (lower)

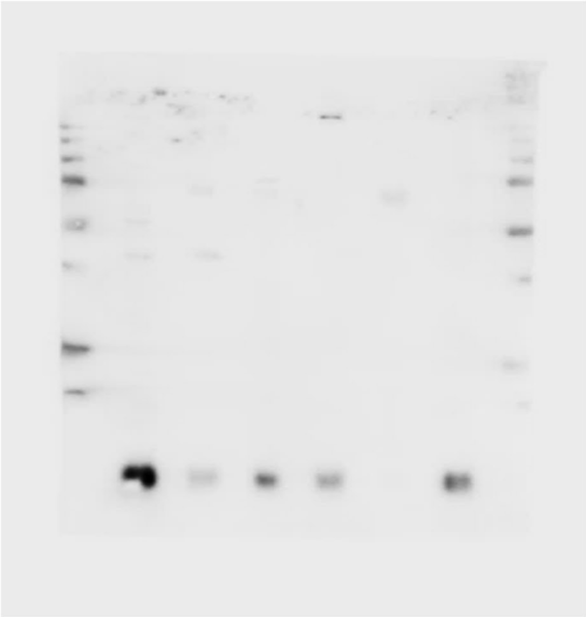

Fig. 2D

HMGB1

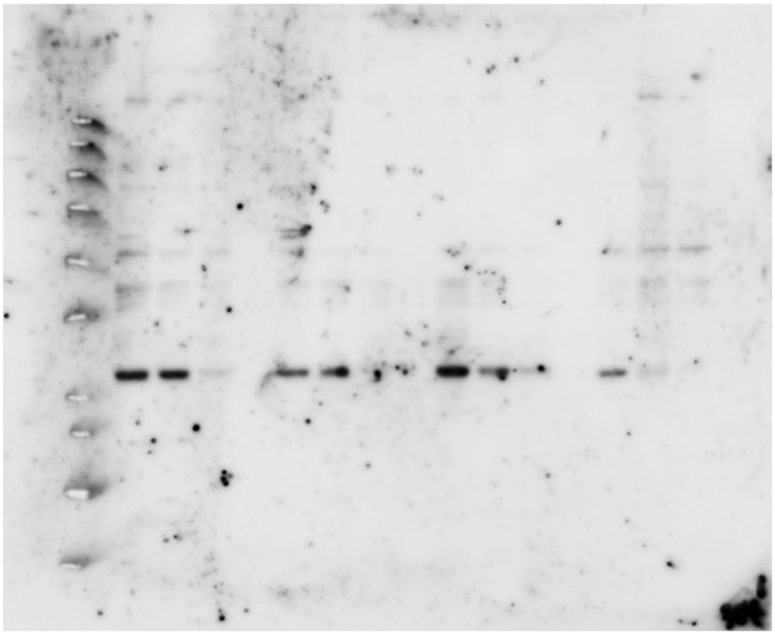

TOM20

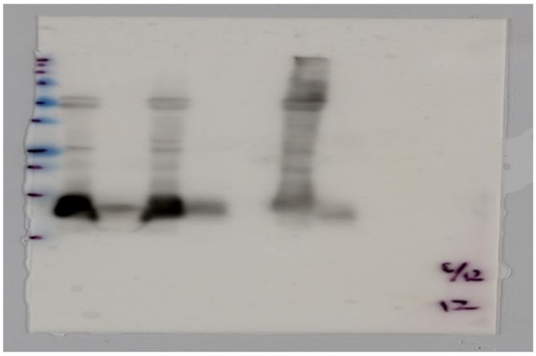

Cytochrome C

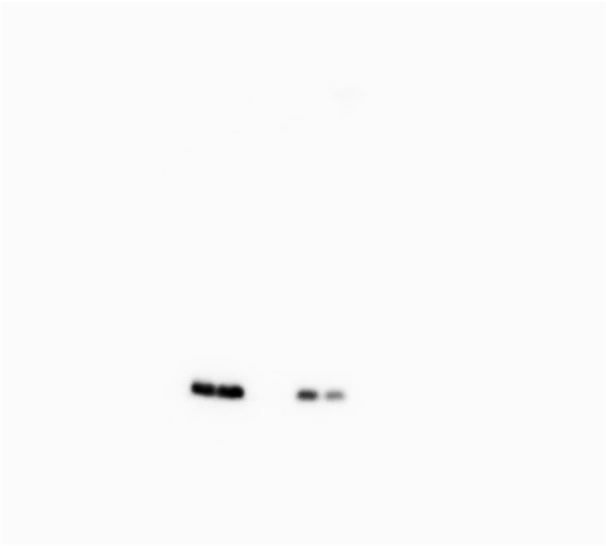

Supplementary source data: original scans of gels

Fig. 2D

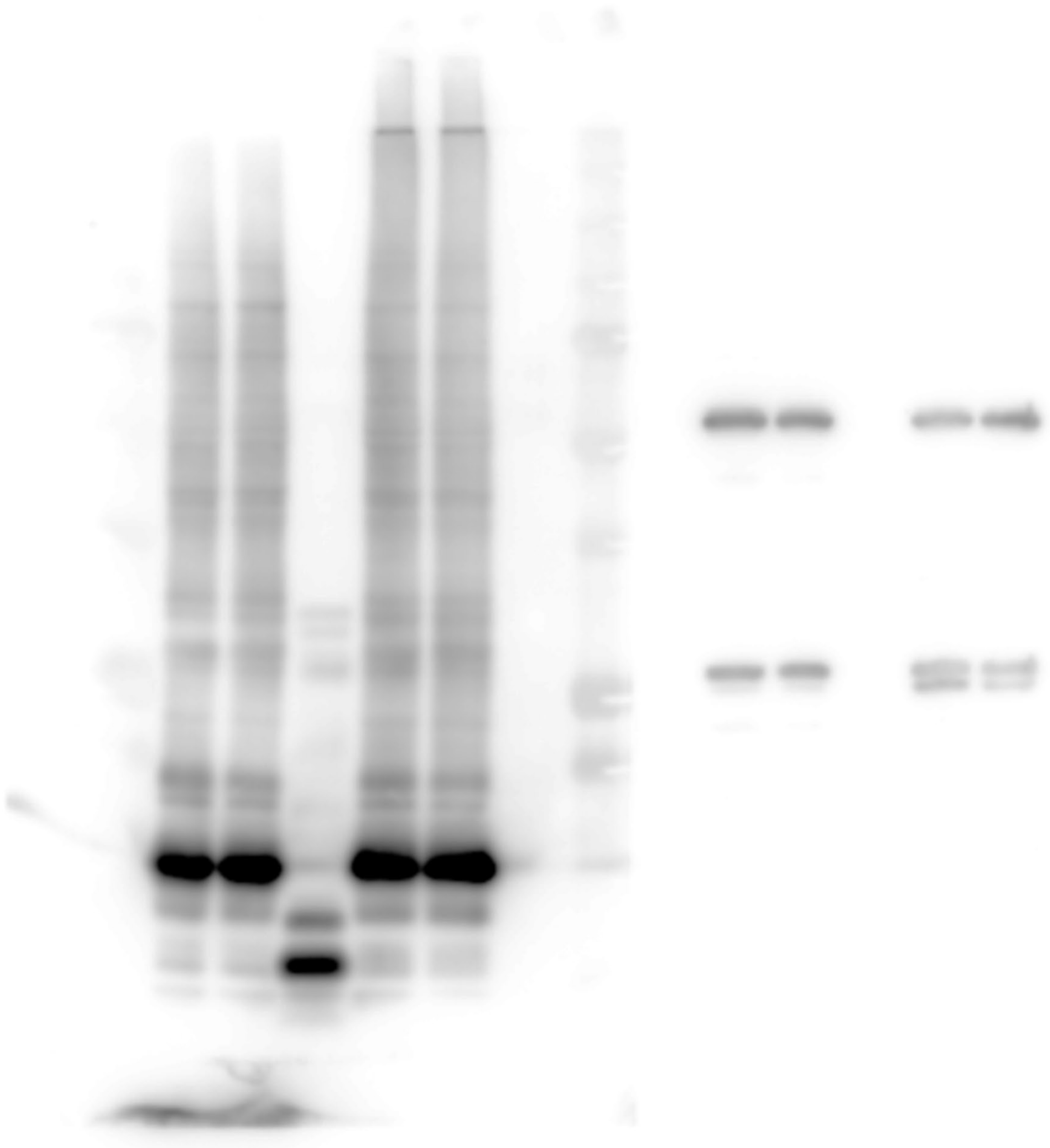

Cox IV TFAM
